# Comparative antibody and cell-mediated immune responses, reactogenicity, and efficacy of homologous and heterologous boosting with CoronaVac and BNT162b2 (Cobovax): an open-label, randomised trial

**DOI:** 10.1016/S2666-5247(23)00216-1

**Published:** 2023-08-04

**Authors:** Nancy H L Leung, Samuel M S Cheng, Carolyn A Cohen, Mario Martín-Sánchez, Niki Y M Au, Leo L H Luk, Leo C H Tsang, Kelvin K H Kwan, Sara Chaothai, Lison W C Fung, Alan W L Cheung, Karl C K Chan, John K C Li, Yvonne Y Ng, Prathanporn Kaewpreedee, Janice Z Jia, Dennis K M Ip, Leo L M Poon, Gabriel M Leung, J S Malik Peiris, Sophie A Valkenburg, Benjamin J Cowling

**Affiliations:** **WHO Collaborating Centre for Infectious Disease Epidemiology and Control** (N H L Leung PhD, S M S Cheng MPhil, M Martín-Sánchez MD, N Y M Au MPH, L L H Luk MSc, L C H Tsang BSc, K K H Kwan BSc, S Chaothai BSc, L W C Fung MMedSc, K C K Chan BSc, J K C Li BSc, Y Y Ng MPH, D K M Ip MD, Prof L L M Poon DPhil, Prof G M Leung MD, Prof J S M Peiris FRCP, Prof B J Cowling PhD) **and HKU-Pasteur Research Pole** (C A Cohen BSc, A W L Cheung MPhil, P Kaewpreedee PhD, J Z Jia MSc, Prof L L M Poon, Prof J S M Peiris, S A Valkenburg PhD), **School of Public Health, Li Ka Shing Faculty of Medicine, The University of Hong Kong, Hong Kong Special Administrative Region, China; Takemi Program in International Health, Harvard T H Chan School of Public Health, Harvard University, Boston, MA, USA** (N H L Leung); **Laboratory of Data Discovery for Health** (N H L Leung, Prof G M Leung, Prof B J Cowling), **and Centre for Immunology and Infection** (Prof J S M Peiris, Prof L L M Poon), **Hong Kong Science and Technology Park, Hong Kong Special Administrative Region, China; Department of Microbiology and Immunology, Peter Doherty Institute of Infection and Immunity, The University of Melbourne, Melbourne, VIC, Australia** (S A Valkenburg)

## Abstract

**Background:**

Few trials have compared homologous and heterologous third doses of COVID-19 vaccination with inactivated vaccines and mRNA vaccines. The aim of this study was to assess immune responses, safety, and efficacy against SARS-CoV-2 infection following homologous or heterologous third-dose COVID-19 vaccination with either one dose of CoronaVac (Sinovac Biotech; inactivated vaccine) or BNT162b2 (Fosun Pharma–BioNTech; mRNA vaccine).

**Methods:**

This is an ongoing, randomised, allocation-concealed, open-label, comparator-controlled trial in adults aged 18 years or older enrolled from the community in Hong Kong, who had received two doses of CoronaVac or BNT162b2 at least 6 months earlier. Participants were randomly assigned, using a computer-generated sequence, in a 1:1 ratio with allocation concealment to receive a (third) dose of CoronaVac or BNT162b2 (ancestral virus strain), stratified by types of previous COVID-19 vaccination (homologous two doses of CoronaVac or BNT162b2). Participants were unmasked to group allocation after vaccination. The primary endpoint was serum neutralising antibodies against the ancestral virus at day 28 after vaccination in each group, measured as plaque reduction neutralisation test (PRNT_50_) geometric mean titre (GMT). Surrogate virus neutralisation test (sVNT) mean inhibition percentage and PRNT_50_ titres against omicron BA.1 and BA.2 subvariants were also measured. Secondary endpoints included geometric mean fold rise (GMFR) in antibody titres; incidence of solicited local and systemic adverse events; IFNγ^+^ CD4^+^ and IFNγ^+^ CD8^+^ T-cell responses at days 7 and 28; and incidence of COVID-19. Within-group comparisons of boost in immunogenicity from baseline and between-group comparisons were done according to intervention received (ie, per protocol) by paired and unpaired *t* test, respectively, and cumulative incidence of infection was compared using Kaplan-Meier curves and a proportional hazards model to estimate hazard ratio. The trial is registered with ClinicalTrials.gov, NCT05057169.

**Findings:**

We enrolled participants from Nov 12, 2021, to Jan 27, 2022. We vaccinated 219 participants who previously received two doses of CoronaVac, including 101 randomly assigned to receive CoronaVac (CC-C) and 118 randomly assigned to receive BNT162b2 (CC-B) as their third dose; and 232 participants who previously received two doses of BNT162b2, including 118 randomly assigned to receive CoronaVac (BB-C) and 114 randomly assigned to receive BNT162b2 (BB-B) as their third dose. The PRNT_50_ GMTs on day 28 against ancestral virus were 109, 905, 92, and 816; against omicron BA.1 were 9, 75, 8, and 86; and against omicron BA.2 were 6, 80, 6, and 67 in the CC-C, CC-B, BB-C, and BB-B groups, respectively. Mean sVNT inhibition percentages on day 28 against ancestral virus were 83%, 96%, 87%, and 96%; against omicron BA.1 were 15%, 58%, 19%, and 69%; and against omicron BA.2 were 43%, 85%, 50%, and 90%, in the CC-C, CC-B, BB-C, and BB-B groups, respectively. Participants who had previously received two doses of CoronaVac and a BNT162b2 third dose had a GMFR of 12 (p<0·0001) compared with those who received a CoronaVac third dose; similarly, those who had received two doses of BNT162b2 and a BNT162b2 third dose had a GMFR of 8 (p<0·0001). No differences in CD4^+^ and CD8^+^ T-cell responses were observed between groups. We did not identify any vaccination-related hospitalisation within 1 month after vaccination. We identified 58 infections when omicron BA.2 was predominantly circulating, with cumulative incidence of 15·3% and 15·4% in the CC-C and CC-B groups, respectively (p=0·93), and 16·7% and 14·0% in the BB-C and BB-B groups, respectively (p=0·56).

**Interpretation:**

Similar levels of incidence of, presumably, omicron BA.2 infections were observed in each group despite very weak antibody responses to BA.2 in the recipients of a CoronaVac third dose. Further research is warranted to identify appropriate correlates of protection for inactivated COVID-19 vaccines.

**Funding:**

Health and Medical Research Fund, Hong Kong.

## Introduction

An unprecedented global effort has led to the rapid development and deployment of COVID-19 vaccines.^1^ Most COVID-19 vaccines have been developed as two doses of the same vaccine technology platform; ie, a homologous two-dose regimen.^1^ The emergence of variants of concern and observed decrease in immune responses within months after vaccination^2^ have led to the implementation of third-dose (so-called booster) vaccination programmes. Although some booster campaigns have encouraged individuals to receive a homologous third dose, a booster dose with a different vaccine platform—ie, heterologous vaccination—could be more feasible or preferable in some locations.^3,4^ It is also possible that combining vaccine doses using different vaccine platforms by heterologous prime-boost strategies might enhance the immune response. Heterologous vaccination has been investigated by several clinical trials with mixed results depending on the initial platform and sequence of vaccination.^3,5,6^

The COVID-19 vaccination programme in Hong Kong (Special Administrative Region, China) initially offered either two doses of CoronaVac (Sinovac Biotech) 28 days apart or two doses of BNT162b2 (Fosun Pharma–BioNTech in mainland China, Hong Kong, Macau, and Taiwan; Pfizer–BioNTech in other regions) 21 days apart, with broad eligibility criteria and availability. Subsequently, adults were recommended to receive a third dose of either vaccine (ie, allowing crossover) as soon as 90 days after the second dose.

Safety, immunogenicity, and efficacy are the main aspects evaluated before vaccines are licensed. Few trials^3^ have compared the potential advantages of homologous or heterologous third doses of inactivated and mRNA vaccine. We conducted a randomised trial of COVID-19 booster vaccinations (Cobovax study) in late 2021 and early 2022 to compare the immunogenicity and reactogenicity with CoronaVac or BNT162b2 in individuals who had previously received two doses of those vaccines.

In addition, Hong Kong had a large wave of infections in early 2022. Most of the population in Hong Kong was unexposed to SARS-CoV-2 infection by any strain until the end of 2021 due to stringent travel restrictions and community-wide non-pharmaceutical interventions. This lack of exposure allowed us to evaluate the comparative efficacy of these different vaccine combinations against infection with the prevalent omicron BA.2 subvariant in a population in whom any previous immunity was from vaccination only.

## Methods

### Study design

This study was an open-label, randomised trial of third-dose COVID-19 vaccine (either BNT162b2 or CoronaVac) conducted in the community in Hong Kong. The study protocol was approved by the institutional review board of the University of Hong Kong (ethics approval number UW 21–492), and is available in appendix 2.

### Participants

Community-dwelling adults were eligible to participate in this study if they were Hong Kong residents, aged 18 years or older at enrolment, and had previously received a complete primary series of either BNT162b2 or CoronaVac, with the second dose received at least 180 days earlier. Exclusion criteria included a history of confirmed SARS-CoV-2 infection; a delay of 43 days or more between the first two vaccine doses; contraindication for COVID-19 vaccination, such as severe allergies, use of medication that could impair the immune system in the past 6 months (except topical steroids or short-term oral steroids), use of immunoglobulins, or any blood products within 90 days of enrolment; and being pregnant or breastfeeding, or planning to become pregnant (appendix 2 p 3). We extended invitation through mass promotion efforts in the community, including advertisements in media channels and mass mailing, bulk university emails, and invitation or referrals to members of existing cohorts. Interested individuals first completed an online questionnaire on the study website for initial eligibility assessment, with eligibility re-confirmed and demographic data collected through interview by the field investigation team during the study visit at enrolment. All participants gave written informed consent for participation in the trial.

### Randomisation and masking

The field investigation team (research assistants and study nurses) performed final participant eligibility screening before enrolling participants during the vaccination visit. The intervention (ie, third-dose vaccination with CoronaVac or BNT162b2) was randomly preassigned to each individual who passed initial eligibility assessment by the online questionnaire system REDCap. The randomisation was based on computer-generated sequences of random numbers produced by NHLL using R software under a block randomisation structure, with block sizes of 2, 4, and 6. Two separate random sequences were generated, one for participants who had previously received two doses of CoronaVac and another for BNT162b2; ie, the four study groups included participants who previously received two doses of CoronaVac and were randomly assigned to receive a third dose of CoronaVac (hereafter CC-C) or BNT162b2 (hereafter CC-B), and participants who previously received two doses of BNT162b2 and were randomly assigned to receive a third dose of CoronaVac (hereafter BB-C) or BNT162b2 (hereafter BB-B). For logistical reasons, individuals were randomly assigned to the intervention at the time vaccination appointments were made; ie, eligible individuals were provided a list of vaccination appointment dates for the same assigned intervention to choose from. There were dropouts after the vaccination appointment was made and before vaccination. However, both the participants and front-line study staff liaising with participants were not aware of the assigned intervention until after vaccination (ie, allocation was concealed) because the vaccination was administered by separate nurses for whom allocation was not concealed: the appointment dates were randomised by computer, and the study principal investigator informed the nurses of the assigned intervention for a vaccination appointment date only a few days beforehand. Nurses with knowledge of group allocation had no further involvement in the trial after administering the intervention. Participants and front-line study staff were unmasked to the type of vaccine received after vaccination through the mandatory government vaccination record. The staff conducting laboratory tests for immunogenicity data were masked to the third-dose allocation until after the laboratory tests were completed. Data on adverse reactions were self-reported by participants with knowledge of group allocation. Data on COVID-19 status based on self-administered rapid antigen tests were self-reported by participants with knowledge of group allocation.

### Procedures

Participants received the assigned intervention (ie, intramuscular administration of one dose of CoronaVac or BNT162b2), during the enrolment and vaccination visit (day 0) at the community vaccination centre. CoronaVac is a Vero cell-based, aluminium hydroxide-adjuvanted, β-propiolactone-inactivated monovalent vaccine, and each 0·5 mL dose includes 600 SU of inactivated SARS-CoV-2. BNT162b2 is a nucleoside-modified mRNA monovalent vaccine encoding the trimerised SARS-CoV-2 spike glycoprotein formulated in lipid nanoparticles, and each 0·3 mL dose contains 30 μg of mRNA. Both vaccines were of the ancestral virus formulation. We collected 20 mL blood samples immediately before vaccination and scheduled follow-up blood draws at 28 days, 182 days, and 365 days after vaccination. In a voluntary subset of participants (appendix 2 p 4), we collected additional 10 mL whole blood samples at days 0, 7, and 28 for analysis of cell-mediated immune responses. After vaccination, participants were requested to record daily, for 7 days or until the last symptom disappeared, whichever was later, any adverse events or symptoms and any medical encounters via an online e-diary. From March 7, 2022, all participants were invited to participate in active surveillance to identify COVID-19 using rapid antigen tests (INDICAID, Phase Scientific; Hong Kong, Special Administrative Region, China) at home every 4 days. In addition, participants were asked to report any PCR-positive result or symptomatic illness. We also ascertained whether any participants had COVID-19 identified by rapid antigen test or PCR after receipt of the third dose, but before the start of active surveillance. The active surveillance is continuing, with information collected up to May 31, 2022 included in the present analyses.

Details of serological testing, including our in-house ELISA for the receptor binding domain (RBD) of the spike protein, a surrogate virus neutralisation test (sVNT; GenScript, Piscataway, NJ, USA) and a plaque reduction neutralisation test (PRNT), have been described in our earlier studies.^4,7,8^ We tested all paired day 0 and day 28 sera with ELISA against ancestral virus, and sVNT against ancestral virus and omicron BA.1 and BA.2 subvariants (appendix 2 p 8). We randomly selected subsets of 20 participants from each study group for testing by PRNT against ancestral virus and omicron BA.1 and BA.2 subvariants on sera,^7^ and separate subsets for cell-mediated immune responses, including cytokine production of IFNγ (in both CD4^+^ and CD8^+^ T cells) and IL-4 (in CD4^+^ T cells only), memory phenotypes (CD45RA and CCR7), and cytokine polyfunctional quality (ie, multiple cytokine production of TNFα and IL-2) by intracellular cytokine staining on peripheral blood mononuclear cells on stimulation by overlapping peptide pool representing the SARS-CoV-2 structural proteins (ie, spike, nucleocapsid, envelope, and membrane).^9^ To explore the relationship between post-vaccination neutralising antibody titre and subsequent risk of infection, we also tested all day 28 sera by PRNT against omicron BA.2 subvariant (the circulating strain). For each serum sample we established the PRNT_50_ titre (ie, the highest serum dilution that neutralises ≥50% of input plaques) and used this titre for immunogenicity comparisons. In addition, in a subset of samples we also established the PRNT_80_ and PRNT_90_ titres (ie, the highest serum dilutions neutralising ≥80% and ≥90% of input plaques, respectively) for future analyses of correlate of protection and protective level. WHO control serum provided by the National Institute for Biological Standards and Control 20/136 gave PRNT_50_ titres of 320 and 320 against the ancestral virus, and 20 and 40 against the omicron variant in two titrations.^7^ Additional information is provided in appendix 2 (pp 3–7).

### Outcomes

The primary outcome measure was vaccine (humoral) immunogenicity at 28 days after the third dose of either BNT162b2 or CoronaVac, measured as geometric mean titre (GMT) of SARS-CoV-2 serum neutralising antibodies against the vaccine strain (ancestral virus) using a plaque reduction neutralisation test (PRNT_50_). The secondary outcome measures included GMT of SARS-CoV-2 serum neutralising antibodies at additional post-vaccination timepoints (days 182 and 365); the geometric mean fold rise (GMFR) of SARS-CoV-2 serum neutralising antibody titres from baseline (day 0) to post-vaccination timepoints (days 28, 182, and 365); vaccine-specific IFNγ^+^ CD4^+^ and IFNγ^+^ CD8^+^ T-cell responses at days 7 and 28; incidence of solicited local and systemic adverse events; incidence of hospitalisations; and incidence of COVID-19 within the year after receiving the third vaccine dose. Antibody responses against other variants of concern will also be assessed as they become available. Results of secondary outcomes listed above that are not reported in this article will be reported in future when data for subsequent timepoints (days 182 and 365) become available.

### Statistical analysis

Based on our preliminary data, assuming a GMT of 27 with an SD of log_2_(GMT) of 0·9, a sample size of 80 individuals per group would provide 80% power to detect a difference in log_2_(GMT) of 0·4 or greater at day 28 from baseline in each study group at the 5% significance level. We aimed for at least 100 individuals in each study group to allow for the possibility of withdrawal from the study before the day 28 assessment.

For vaccine immunogenicity (primary outcome), we included data from all participants who received the assigned vaccination and provided days 0 and 28 sera samples in our trial (ie, per-protocol analysis). For adverse events, we included all available data. For SARS-CoV-2 infection, we included data from participants who agreed to participate in active surveillance and had reported at least once self-administered rapid antigen test result from the systematic monitoring, and had also reported information on any infection that occurred before active surveillance started.

For SARS-CoV-2 serum neutralising antibody PRNT_50_ titres, we imputed titres of less than 10 with the value 5 and titres of 1280 or more with the value 2560, and then log_2_ transformed to estimate the GMT. A similar procedure was done for PRNT_50_, PRNT_80_, and PRNT_90_ titres. We also estimated the arithmetic mean of SARS-CoV-2 sVNT neutralisation percentages and ELISA spike-RBD IgG concentration (proxy by optical density [OD_450_]). For cell-mediated response, we defined the threshold specific to that response as the lowest positive value from all samples collected from all days (CD4^+^ IFNγ^+^ T cells 0·001%; CD8^+^ IFNγ^+^ T cells 0·00017%; and CD4^+^ IL4^+^ T cells 0·000042%). A sample with a value above the threshold was classified as a responder. We imputed the values for the non-responders with the value of the respective threshold for plotting and calculating the mean response. We transformed the peptide-specific CD4^+^ IFNγ^+^ T-cell responses, CD8^+^ IFNγ^+^ T-cell responses, and CD4^+^ IL4^+^ T-cell responses to log_10_ scale to estimate geometric means. We compared antibody levels and T-cell responses between timepoints from the same individuals using a paired *t* test, and between study groups using an unpaired *t* test, with p values of 0·05 or less considered significant. In post-hoc sensitivity analysis, we performed linear regression on log_2_ PRNT_50_ titre with adjustment of covariates that were imbalanced at baseline. In comparing the percentage of each compartment of memory phenotypes or cytokine polyfunctional quality in IFNγ^+^ CD4^+^ T cells or IFNγ^+^ CD8^+^ T cells between timepoints or between study groups, 0% was assumed for individuals without IFNγ-producing CD4^+^ or CD8^+^ T cells.

We conducted complete case analyses for reactogenicity and incidence of SARS-CoV-2 infection. Rates of solicited local and systemic adverse events or symptoms were compared between study groups using Pearson’s χ^2^ test or Fisher’s exact test, and p values of 0·05 or less were considered significant. Cumulative incidence of SARS-CoV-2 infections between study groups were compared using Kaplan-Meier curves and proportional hazards models to estimate the hazard ratio for a BNT162b2 third dose compared with a CoronaVac third dose with 95% CI. The model was specified on a calendar time axis with follow-up starting from the start of Hong Kong’s fifth wave on Jan 1, 2022, or on the date of third dose if later, and ending at the earliest of the time of infection, the time of receiving a fourth dose, the time of withdrawing from the study, or the censoring date on May 31, 2022. If a participant reported more than one positive result, the earliest date of identification was considered the time of infection. All statistical analyses were done with R, version 4.3.0.

A data monitoring committee was deemed unnecessary because both vaccines had obtained emergency use approval in the community. The study is registered in ClinicalTrials.gov (NCT05057169).

### Role of the funding source

The funder of the study had no role in study design, data collection, data analysis, data interpretation, or writing of the report.

## Results

**Figure 1:**
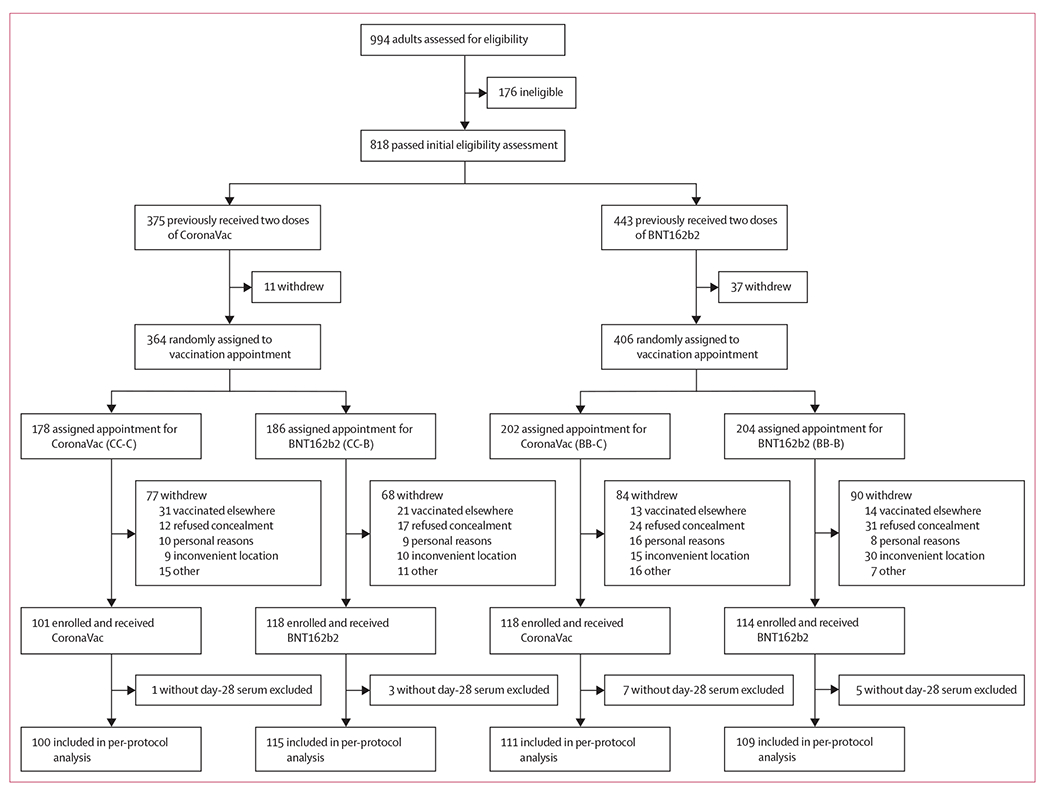
Trial profile A more detailed flow chart with reasons for exclusion at each stage is provided in appendix 2 (p 31).

From Nov 12, 2021, to Jan 27, 2022, we screened 994 individuals, and 818 (82%) passed the initial eligibility assessment (figure 1; appendix 2 p 31). We randomly assigned 364 individuals to the CC-C (n=178) and CC-B (n=186) groups, and 406 individuals to the BB-C (n=202) and BB-B (n=204) groups. After final confirmation of eligibility, we collected baseline (day 0) blood samples from 451 participants and then administered third doses, including to 101 (57%) of 178 participants in the CC-C group, 118 (63%) of 186 in the CC-B group, 118 (58%) of 202 in the BB-C group, and 114 (56%) of 204 in the BB-B group. Participant characteristics were generally comparable between the CC-C and CC-B groups, and between the BB-C and BB-B groups (table 1). Participants in the CC-C group (mean age 55 years [SD 12]) were slightly older than those in the CC-B group (51 years [11]; p=0·0050), and more participants in the BB-B group (19 [17%] of 114) had hypertension than in the BB-C group (seven [6%] of 118; p=0·029). For all 451 vaccinated participants, most reported receiving the second dose 6–8 months earlier (table 1). A subset of 156 (35%) of 451 participants agreed to provide samples of peripheral blood mononuclear cells on days 0, 7, and 28 to evaluate cell-mediated immune responses.

We collected information on post-vaccination reactions in 424 (94%) of 451 participants, including 193 (46%) of 424 who reported ever feeling unwell after vaccination, with significantly more frequent reactions in recipients of a third dose of BNT162b2 that mostly subsided within 7 days (table 2; appendix 2 p 34). Fever of 38·0°C or higher was reported by 11 (10%) of 109 participants in the BB-B group and 12 (11%) of 112 participants in the CC-B group. No participants in the BB-C and CC-C groups reported fever of 38·0°C or higher (table 2). Among 440 participants who reported information on medical care and hospitalisation, ten reported seeking ambulatory care within 1 month of the third dose, including four (one in the CC-C group and three in the CC-B group) for discomfort associated with vaccination (appendix 2 p 13). One participant in the BB-B group reported hospitalisation within 1 month after vaccination for unrelated reasons (appendix 2 p 13).

**Figure 2:**
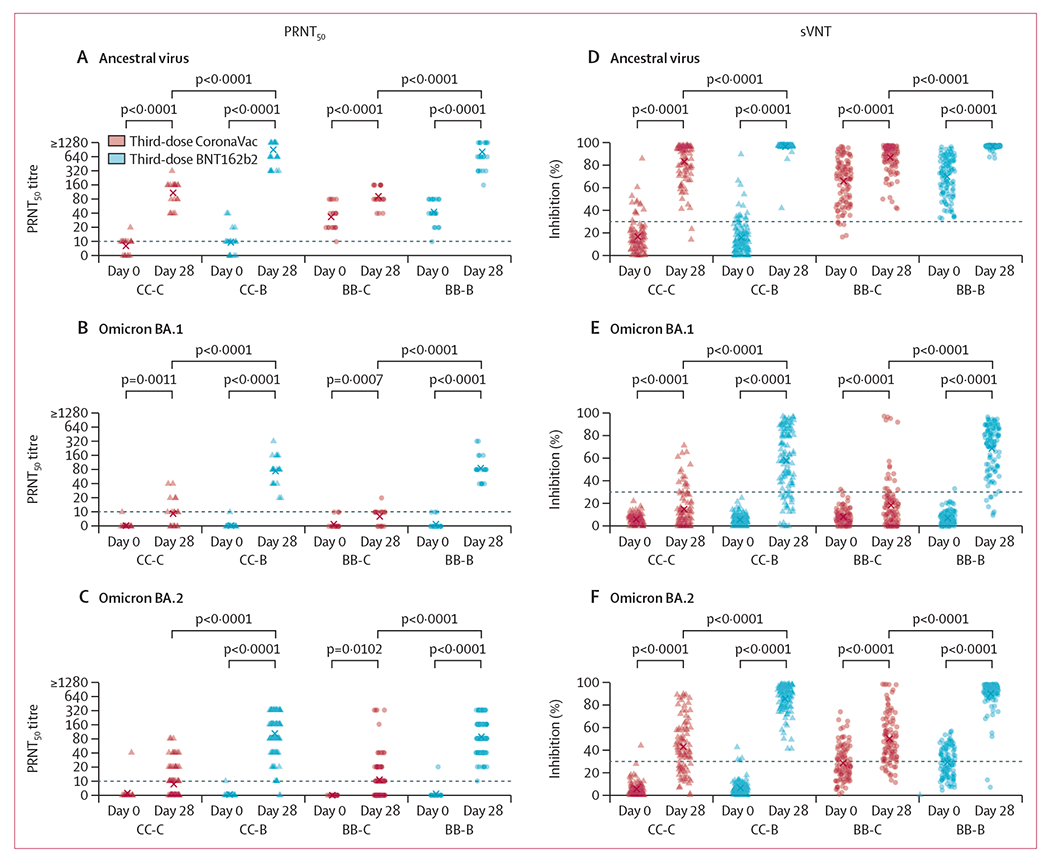
Serum neutralising antibodies measured by live virus PRNT (A–C) or by sVNT (D–F) against ancestral SARS-CoV-2, omicron BA.1, and omicron BA.2, respectively, at baseline and 28 days after randomised third-dose CoronaVac or BNT162b2 vaccination PRNT titres were evaluated with endpoint at 50% inhibition (PRNT_50_). The four study groups included participants who previously received two doses of CoronaVac and were randomly assigned to receive a third dose of CoronaVac (CC-C) or BNT162b2 (CC-B), and participants who previously received two doses of BNT162b2 and were randomly assigned to receive a third dose of CoronaVac (BB-C) or BNT162b2 (BB-B). Dotted lines represent seropositivity (titre ≥10) in the in-house PRNT and the manufacturer’s threshold for seropositivity of at least 30% in the sVNT assays. Data on sVNT against ancestral virus, omicron BA.1 and BA.2 variants on sera collected at baseline and day 28 post vaccination from all participants (with paired sera) were included (appendix 2 p 14). Data on PRNT against omicron BA.2 variant on sera collected on day 28 post vaccination from all participants (appendix 2 p 23), and on sera collected at baseline obtained from a random subset of 20 participants selected from each study group (appendix 2 p 14), were included. Data on PRNT against ancestral virus and omicron BA.1 variant on sera collected at baseline and day 28 from a random subset of 20 participants selected from each study group were included (appendix 2 p 14). PRNT=plaque reduction neutralisation test. sVNT=surrogate virus neutralisation test.

We measured sera neutralising antibodies from third-dose vaccination in 435 participants who provided paired day 0 and day 28 sera after vaccination (appendix 2 p 8). Third-dose vaccination significantly increased neutralising antibodies, measured as PRNT_50_ titres or sVNT inhibition percentage, against the ancestral virus, omicron BA.1, and omicron BA.2 on day 28 from baseline in all four study groups, regardless of third-dose vaccine type (figure 2; appendix 2 pp 14–15, 35). For plaque reduction neutralising antibodies in a random subset of 20 participants from each group (appendix 2 p 10), the PRNT_50_ GMTs at baseline against ancestral virus were 8, 10, 34, and 43; against omicron BA.1 were 5, 5, 6, and 6; and against omicron BA.2 were 6, 5, 5, and 5, in the CC-C, CC-B, BB-C, and BB-B groups, respectively. PRNT_50_ GMTs on day 28 against ancestral virus were 109, 905, 92, and 816; against omicron BA.1 were 9, 75, 8, and 86; and against omicron BA.2 were 6, 80, 6, and 67, in the CC-C, CC-B, BB-C, and BB-B groups, respectively (appendix 2 pp 14, 36). Thus, third-dose vaccination increased PRNT_50_ titres against ancestral virus by 14-fold, 94-fold, 3-fold, and 19-fold; against omicron BA.1 by 2 fold, 14-fold, 2-fold, and 16-fold; and against omicron BA.2 by 1-fold, 16-fold, 1-fold, and 13-fold in the CC-C, CC-B, BB-C, and BB-B groups, respectively (appendix 2 p 36). For surrogate neutralising antibodies in all participants, the mean sVNT inhibition percentages at baseline before third-dose vaccination (day 0) against ancestral virus were 17%, 16%, 66%, and 70%; against omicron BA.1 were 6%, 6%, 8%, and 7%; and against omicron BA.2 were 5%, 6%, 28%, and 29%, in the CC-C, CC-B, BB-C, and BB-B groups, respectively. Mean sVNT inhibition percentages on day 28 against ancestral virus were 83%, 96%, 87%, and 96%; against omicron BA.1 were 15%, 58%, 19%, and 69%; and against omicron BA.2 were 43%, 85%, 50%, and 90%, in the CC-C, CC-B, BB-C, and BB-B groups, respectively (figure 2; appendix 2 p 14). Thus, third-dose vaccination increased sVNT inhibition percentage against ancestral virus by 5·0-fold, 6·1-fold, 1·3-fold, and 1·4-fold; against omicron BA.1 by 2.5-fold, 10·2-fold, 2·2-fold, and 9·6-fold; and against omicron BA.2 by 8·0-fold, 13·6-fold, 1·8-fold, and 3·1-fold, in the CC-C, CC-B, BB-C, and BB-B groups, respectively (appendix 2 p 14 ). In both assays, antibody responses to a BNT162b2 third dose were substantially and significantly greater than responses to a CoronaVac third dose regardless of previous two-dose vaccine type (figure 2; appendix 2 pp 19–20, 35). The PRNT_50_ GMT titres for neutralising antibodies against omicron BA.2 on day 28 when testing was done in all participants was 9 in the CC-C group, 102 in the CC-B group, 11 in the BB-C group, and 87 in the BB-B group. Participants who had previously received two doses of CoronaVac and a BNT162b2 third dose had a GMFR of 12 compared with those who received a CoronaVac third dose (p<0·0001); similarly, participants who previously received two doses of BNT162b2 and BNT162b2 as their third dose had a GMFR of 8 (p<0·0001; figure 2; appendix 2 p 23). The differences in PRNT_50_ titres after a CoronaVac versus BNT162b2 third dose remained significant after adjusting for age alone (p<0·0001), hypertension alone (p<0·0001), or both (p<0·0001; appendix 2 p 24). Correlations between PRNT_50_ titres and sVNT inhibition percentage ranged from 0·48 to 0·73 (appendix 2 p 37). Similar significant differences between study groups were observed when comparing binding antibodies with ancestral virus by ELISA (appendix 2 pp 19–20, 38), or when comparing neutralising antibodies evaluated at higher neutralisation inhibition thresholds (PRNT_80_ or PRNT_90_ titres; appendix 2 pp 19–20, 23, 39).

**Figure 3:**
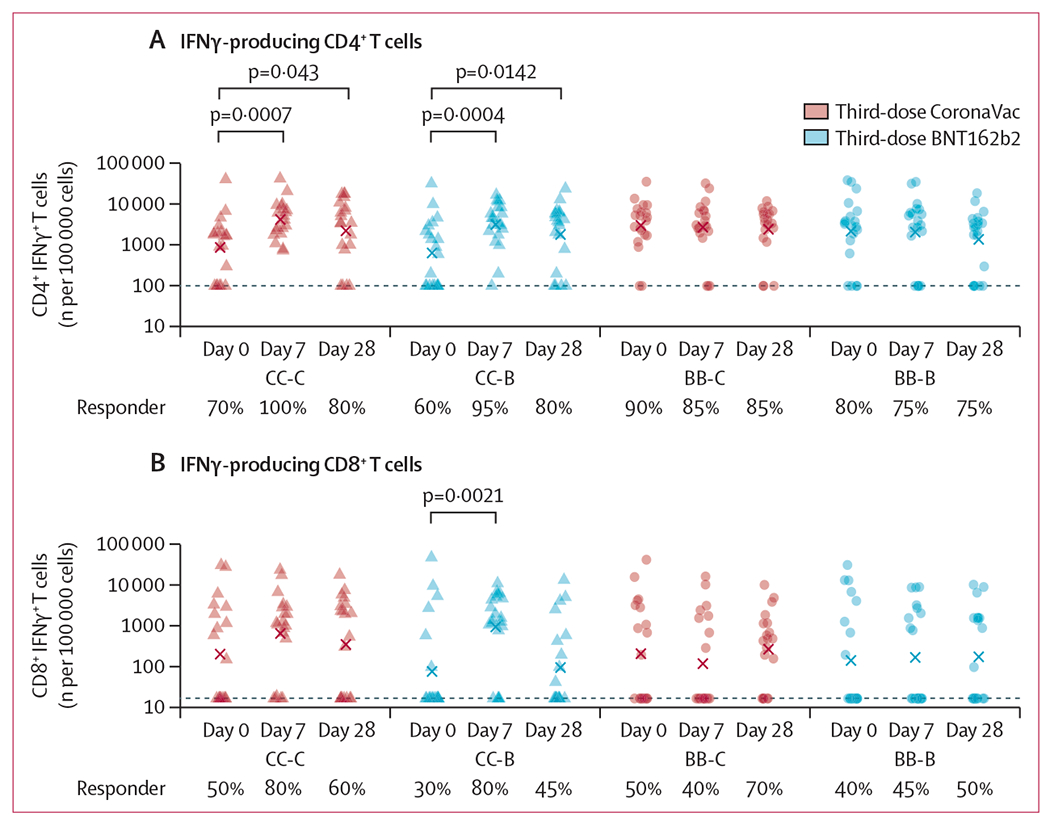
IFNγ-producing CD4^+^ (A) and CD8^+^ (B) T-cell responses against structural peptides of ancestral SARS-CoV-2 at baseline, and 7 days and 28 days after third-dose vaccination with CoronaVac or BNT162b2 The four study groups included participants who previously received two doses of CoronaVac and were randomly assigned to receive a third dose of CoronaVac (CC-C) or BNT162b2 (CC-B), and participants who previously received two doses of BNT162b2 and were randomly assigned to receive a third dose of CoronaVac (BB-C) or BNT162b2 (BB-B). Data were available from a random subset of 20 participants selected from each study group (appendix 2 p 16). Dotted lines represent the threshold specific to that response, which we defined as the lowest positive value from all samples collected from all days (0, 7, and 28) and participants with values above these thresholds are considered as responders: 0·001% (equivalent to 100 per 100 000 cells) for CD4^+^ IFNγ^+^ T cells and 0·00017% (equivalent to 17 per 100 000 cells) for CD8^+^ IFNγ^+^ T cells. The proportions of participants who were responders were listed below each timepoint and can be found in appendix 2 (p 25).

In a random subset of 20 participants from each group (appendix 2 p 10), third-dose vaccination significantly increased the proportion of IFNγ^+^ CD4^+^ T cells in both the CC-C and CC-B groups on day 7, which remained significantly elevated on day 28, but not in the BB-C and BB-B groups (figure 3A; appendix 2 pp 16, 36). At least 30–50% of participants in all groups had IFNγ^+^ CD8^+^ T cells at baseline, but these were only significantly boosted in the CC-B group on day 7 and contracted by day 28 (figure 3B; appendix 2 pp 25, 36). IL4^+^ CD4^+^ T cells were not significantly boosted by third doses in any group (appendix 2 pp 36, 40). There were no significant differences in the magnitude of CD4^+^ and CD8^+^ T-cell responses nor proportion of responders between the CC-C and CC-B groups, nor between the BB-C and BB-B groups by 28 days after vaccination (appendix 2 pp 21, 25). The gating strategy is shown in appendix 2 (p 41), as are representative plots for responders and non-responders (pp 42–43). We also evaluated the multiple cytokine production (TNFα and IL-2) and memory phenotypes of IFNγ-producing CD4^+^ or CD8^+^ T cells (appendix 2 pp 16–18, 21–22, 44). We observed a bias towards single cytokine responses (IFNγ^+^) as the majority of the IFNγ-producing CD4^+^ or CD8^+^ T-cell responses at baseline across all groups, and noted expansions of double cytokine responses (IFNγ^+^ with TNFα^+^ or IL-2^+^) for CD4^+^ T cells on third-dose vaccination in the CC-C and CC-B groups. T-effector memory responses predominated at baseline across all groups, which again were further expanded on third-dose vaccination in the CC-C and CC-B groups (appendix 2 pp 16–18, 21–22, 44). Across the two (non-randomised) strata, participants who previously received two doses of CoronaVac (ie, the CC-C and CC-B groups) received the randomly assigned third dose a mean of 231 days (SD 26) after the second dose, whereas participants who previously received two doses of BNT162b2 (ie, the BB-C and BB-B groups) received the randomly assigned third dose a mean of 224 days (31) after the second dose (ie, 12 days difference; appendix 2 p 26). The former also had lower antibody response at baseline (day 0) shortly before third-dose vaccination than the latter (appendix 2 p 27). After third-dose vaccination, the proportions of former participants who were recorded as T-cell responders on day 28 (CD4^+^ 80%, CD8^+^ 45–60%; figure 3; appendix 2 p 25) were similar to the proportions of latter participants who were recorded as T-cell responders at baseline (CD4^+^ 80–90%, CD8^+^ 40·50%; figure 3; appendix 2 p 25).

**Figure 4:**
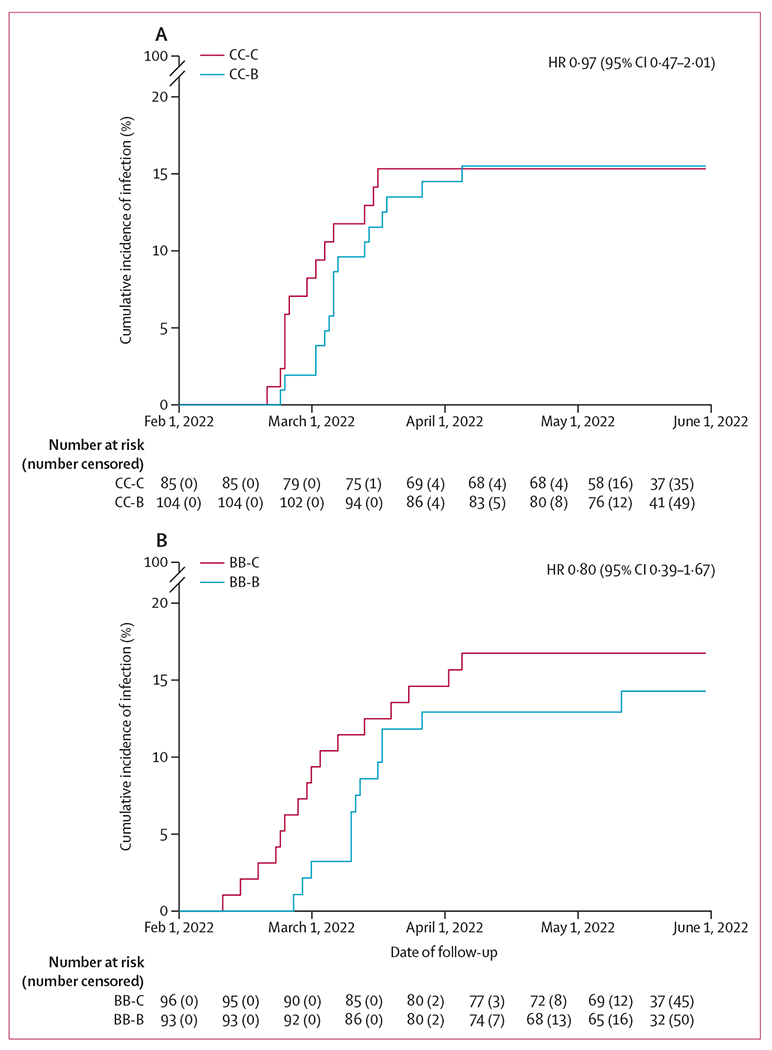
Cumulative incidence of SARS-CoV-2 infection after third-dose CoronaVac or BNT162b2 vaccination in adults who previously received two doses of CoronaVac (A) or BNT162b2 (B) within 4–6 months after vaccination and when the omicron BA.2 subvariant was locally circulating COVID-19 was identified by rapid antigen test or PCR between third-dose vaccination (administered from Nov 12, 2021, to Jan 27, 2022) until May 31, 2022. The four study groups included participants who previously received two doses of CoronaVac and were randomly assigned to receive a third dose of CoronaVac (CC-C) or BNT162b2 (CC-B), and participants who previously received two doses of BNT162b2 and were randomly assigned to receive a third dose of CoronaVac (BB-C) or BNT162b2 (BB-B). Data from 378 (84%) of 451 participants who agreed to participate in active surveillance for respiratory illness were included. The date of a positive test for the first SARS-CoV-2 infection identified in these participants was Feb 11, 2022. HR=hazard ratio.

Data on SARS-CoV-2 infection from 378 (84%) of 451 participants were analysed, including 85 (84%) of 101 in the CC-C group, 104 (88%) of 118 in the CC-B group, 96 (81%) of 118 in the BB-C group, and 93 (82%) of 114 in the BB-B group. After receiving third-dose vaccination, and before active surveillance started, 42 infections were identified either by rapid antigen test or PCR, including in ten participants in the CC-C group, 11 in the CC-B group, 11 in the BB-C group, and ten in the BB-B group. During active surveillance from March 11 to May 31, 2022, participants reported a rapid antigen test result, on average, every 4·5 days for a median of 69 days (IQR 60–76). 24 infections were identified either by systematic rapid antigen test or self-reported PCR, including three participants in the CC-C group, eight in the CC-B group, five in the BB-C group, and eight in the BB-B group. A daily symptom diary was initiated for 53 illness episodes from 52 participants, from which 22 infections were identified by rapid antigen test or PCR, including three participants in the CC-C group, nine in the CC-B group, three in the BB-C group, and seven in the BB-B group. Together, we identified 58 SARS-CoV-2 infections by May 31, 2022 (appendix 2 pp 28–29), within 4–6 months of receipt of third-dose vaccination and during the period when the omicron BA.2 subvariant was locally circulating, including 13 (15%) of 85 participants in the CC-C group, 16 (15%) of 104 in the CC-B group, 16 (17%) of 96 in the BB-C group, and 13 (14%) of 93 in the BB-B group, with no statistically significant difference detected between CC-C and CC-B groups (p=0·9325) nor between the BB-C and BB-B groups (p=0·56; figure 4). Comparing the BB-B and BB-C groups, the hazard ratio for infection following a BNT162b2 third dose compared with a CoronaVac third dose was 0·80 (95% CI 0·39–1·67) corresponding to a relative vaccine efficacy against infection of 20%, while the hazard ratio for the CC-B group versus the CC-C group was 0·97 (95% CI 0·47–2·01) corresponding to a relative vaccine efficacy of 3%. A list of all infections, method of identification, and verification is given in appendix 2 (p 28).

## Discussion

Here, we have conducted a comprehensive assessment of the reactogenicity, antibody response, T-cell responses and risk of omicron BA.2 infection in a randomised trial of homologous and heterologous boosting with BNT162b2 and CoronaVac, both of which used ancestral virus as the vaccine strain. Our population had minimal COVID-19 history before all three doses of vaccination,^10^ and absence of known previous SARS-CoV-2 infection was one of the inclusion criteria. Regardless of previous (inactivated or mRNA) vaccine type, more frequent post-vaccination reactions were observed in recipients of third-dose BNT162b2, although most subsided within the week after vaccination; and no vaccine-related hospitalisations were identified within 1 month for all study groups. In all participants in whom we assessed the primary outcome of neutralising antibody response, regardless of homologous or heterologous vaccine combinations, a third vaccine dose significantly increased neutralising antibody against the ancestral virus and the omicron BA.1 and BA.2 variants from baseline; and as third-dose booster, mRNA vaccines have substantially greater neutralising antibody response compared with inactivated vaccines regardless of previous vaccine type. In a subset of participants in whom we explored the secondary outcome of cell-mediated response, CD4^+^ helper T-cell responses were only boosted and maintained by third-dose CoronaVac or BNT162b2 in participants who previously received two doses of CoronaVac; there were no significant differences in both CD4^+^ and CD8^+^ T-cell responses between all study groups post third-dose vaccination. Although this study was not designed as an efficacy trial, was not powered to assess differences in infection rates between groups, and significant differences in neutralising antibody response between groups were observed (including against the predominant circulating strain omicron BA.2), the incidence of SARS-CoV-2 infection was similar between groups 4–6 months after vaccination.

Neutralising antibodies were shown to be correlates of protection when comparing mRNA and adenovirus vectored vaccines, and in comparisons between vaccinated and unvaccinated individuals.^11–14^ By contrast, our results comparing individuals vaccinated with third-dose CoronaVac or BNT162b2 showed that, at least in our study, neutralising antibodies against the circulating omicron variant did not correlate with protection against infection. This finding is biologically plausible—antibody alone might not explain the protection provided by inactivated vaccines, as T-cell responses and potentially non-neutralising antibodies to internal virus genes, such as the nucleocapsid,^15^ might also play a role, especially against strains such as omicron, which have high escape rate from neutralising antibody elicited by the ancestral virus. For T-cell response, previous studies using vaccinated transgenic mice with humanised ACE2 receptor showed that CD4^+^ and CD8^+^ T cells protected against severe disease by ancestral strain^16^ and CD4^+^ T cells conferred heterologous protection against the beta variant,^17^ and experiments using human samples suggested T-cell cross-reactivity against omicron.^18,19^ Our study provides a head-to-head comparison of T-cell response between homologous and heterologous vaccination, and shows that both homologous and heterologous third doses could boost T-cell responses in people with two previous doses of CoronaVac. We also show that a possible ceiling effect exists in those with two previous doses of BNT162b2 within 6–8 months after the second dose, although sample size was small and further confirmation is warranted. For vaccine effectiveness, a separate observational cohort study from our group in Hong Kong during a similar period reported similar vaccine effectiveness against both asymptomatic and symptomatic infection by the omicron BA.2 subvariant between CC-C and BB-B homologous vaccination,^20^ and we also noted similar vaccine effectiveness against severe disease on the basis of individual-level case data supplemented with census information and vaccine coverage data.^21^ Our present findings in a randomised setting suggest similar vaccine efficacy against infection between homologous and heterologous vaccination of mRNA and inactivated COVID-19 vaccines.

Our study has several limitations. First, due to logistical challenges, we were only able to randomise at the time vaccination appointment was made, but not at vaccination, and nearly half of randomly assigned individuals dropped out before vaccination. However, the rates of dropout were similar across study groups, and individuals dropping out were not aware of their allocated vaccine type. Comparisons between study groups did not identify significant differences in most baseline characteristics measured, nor any significant differences in antibody and cell-mediated response measured at baseline. Second, there were baseline age differences between the CC-C and CC-B groups, and differences in the proportion of participants with hypertension between the BB-C and BB-B groups, but the differences in neutralising antibodies after third-dose vaccination of CoronaVac versus BNT162b2 remained statistically significant after adjusting for age and hypertension status. Third, we did not include an unvaccinated control group, nor a two-dose comparison group, to evaluate the additional benefits of third dose over existing two doses. Fourth, we have only studied neutralising antibodies and T-cell response, and other branches of immunity, such as non-neutralising antibodies,^22,23^ might also contribute to protection and explain the discrepancy in our immunological and efficacy data. Finally, our study population consists of adults in whom first immune priming was from either mRNA or inactivated vaccination, which is different to many parts of the world, where the first immune priming was from natural infection. Differences in priming might lead to differences in subsequent immune response,^24^ vaccine effectiveness,^25^ and duration of protection.^26^

Most populations received a primary series with mRNA or inactivated COVID-19 vaccines; thus, our findings have wide relevance to booster vaccination policies around the world. Differences in neutralising antibody response are interpreted as indicative of superior efficacy against infection, and have been used as correlate of protection in immunobridging studies for COVID-19 vaccines. The observed discrepancy between neutralising antibody response and vaccine efficacy against infection in our trial might indicate the challenge of using neutralising antibody as the only correlate or mediator of protection against infection,^27,28^ at least in comparisons between vaccination strategies of mRNA and inactivated vaccines, among individuals previously vaccinated. The potential differences between vaccine types in their relationship between correlates of protection and protection might not always have been accounted for in immunobridging studies.^29^

In conclusion, our results suggest there is immune benefit in both administering a homologous or heterologous third dose 6 months after two doses of inactivated or mRNA vaccination, especially in adults who initially received two doses of an inactivated vaccine, with similar levels of protection against infection provided by all four combinations. Mass vaccination programmes that offer both inactivated and mRNA vaccines will allow flexibility in vaccine deployment and encourage vaccine uptake. Our finding that neutralising antibody might not be the dominant correlate of protection for inactivated vaccines, especially against SARS-CoV-2 variants, such as omicron, that have significant capacity to evade neutralising antibody, needs to be considered in the development of variant-proof COVID-19 vaccines or vaccines broadly protective against sarbecoviruses currently in development.^30^

## Supplementary Material

1

2

## Figures and Tables

**Table 1:** Baseline characteristics

	All enrolled and received third-dose vaccination (N=451)	CC-C (n=101)	CC-B (n=118)	p value	BB-C (n=118)	BB-B (n=114)	p value
Sex							
Female	221 (49%)	47 (47%)	64 (54%)	0·13	51 (43%)	59 (52%)	0·50
Male	230 (51%)	54 (53%)	54 (46%)	1·0	67 (57%)	55 (48%)	0·32

Age, years	53 (11)	55 (12)	51 (11)	0·0050	53 (11)	52 (12)	0·55

Ethnicity	NA	NA	NA	0·36	NA	NA	0·28
Chinese	443 (98%)	100 (99%)	117 (99%)	NA	116 (98%)	111 (97%)	NA
White	3 (1%)	1 (1%)	0	NA	2 (2%)	0	NA
Japanese	2 (<1%)	0	1 (1%)	NA	0	1 (1%)	NA
Latin American	1 (<1%)	0	0	NA	0	1 (1%)	NA
Pakistani	1 (<1%)	0	0	NA	0	1 (1%)	NA

Weight group (for Asian populations)	NA	NA	NA	0·97	NA	NA	0·59
Underweight, BMI <18·5	23 (5%)	6 (6%)	6 (5%)	NA	4 (3%)	7 (6%)	NA
Normal, BMI 18·5–22·9	187 (41%)	45 (45%)	52 (44%)	NA	50 (42%)	40 (35%)	NA
Overweight, BMI 23·0–24·9	99 (22%)	20 (20%)	22 (19%)	NA	28 (24%)	29 (25%)	NA
Obese, BMI ≥25·0	142 (31%)	30 (30%)	38 (32%)	NA	36 (31%)	38 (33%)	NA

Chronic medical conditions							
Any	104 (23%)	21 (21%)	21 (18%)	>0·99	30 (25%)	32 (28%)	0·90
Lung disease, including COPD and asthma	3 (1%)	2 (2%)	0	0·50	0	1 (1%)	>0·99
Heart disease	5 (1%)	2 (2%)	1 (1%)	>0·99	2 (2%)	0	0·50
Hypertension	45 (10%)	9 (9%)	10 (8%)	>0·99	7 (6%)	19 (17%)	0·029
Diabetes	13 (3%)	6 (6%)	2 (2%)	0·29	3 (3%)	2 (2%)	>0·99
Hypercholesterolaemia	28 (6%)	7 (7%)	3 (3%)	0·34	9 (8%)	9 (8%)	>0·99
Kidney disease	1 (<1%)	0	0	>0·99	1 (1%)	0	>0·99
Liver disease	7 (2%)	1 (1%)	2 (2%)	>0·99	2 (2%)	2 (2%)	>0·99
Cancer	6 (1%)	3 (3%)	2 (2%)	>0·99	0	1 (1%)	>0·99

Time between first and second dose of COVID-19 vaccination, days	26 (4)	29 (2)	29 (2)	0·59	22 (2)	22 (2)	0·63

Time between second and third (study) dose of COVID-19 vaccination, days	227 (29)	229 (26)	232 (27)	0·51	224 (30)	224 (31)	0·98

Smoking							
Ever	20 (4%)	8 (8%)	7 (6%)	>0·99	2 (2%)	3 (3%)	>0·99
Current	11 (2%)	4 (4%)	5 (4%)	>0·99	1 (1%)	1 (1%)	>0·99

Data are n (%) or mean (SD). The four study groups included participants who previously received two doses of CoronaVac and were randomly assigned to receive a homologous third dose of CoronaVac (CC-C) or a heterologous third dose of BNT162b2 (CC-B), and participants who previously received two doses of BNT162b2 and were randomly assigned to receive a heterologous third dose of CoronaVac (BB-C) or a homologous third dose of BNT162b2 (BB-B). NA=not applicable. COPD=chronic obstructive pulmonary disease.

**Table 2:** Solicited local and systemic reactions during the 7 days after third-dose CoronaVac or BNT162b2 vaccination

	CC-C (n=91)	CC-B (n=112)	p value	BB-C (n=112)	BB-B (n=109)	p value
Reported ever feeling unwell	24 (26%)	77 (69%)	<0·0001	25 (22%)	67 (61%)	<0·0001

Duration of adverse reactions, days	2·2 (1·8)	3·6 (2·1)	0·025	2·2 (1·4)	3·0 (1·6)	0·15

Severity score for interfering with usual activities	0·08 (0·18)	0·40 (0·44)	<0·0001	0·07 (0·17)	0·31 (0·34)	<0·0001

Body temperature when fever was reported, °C	··	38·2 (38·0–38·4)	··	··	38·4 (38·3–38·6)	··

Solicited local reactions						
Pain	8 (9%)	59 (53%)	<0·0001	10 (9%)	50 (46%)	<0·0001
Tenderness	8 (9%)	57 (51%)	<0·0001	5 (4%)	43 (39%)	<0·0001
Swelling or hardness	5 (5%)	30 (27%)	0·0001	1 (1%)	21 (19%)	<0·0001
Itchiness	2 (2%)	11 (10%)	0·027	0	9 (8%)	0·0015
Redness	1 (1%)	9 (8%)	0·025	0	6 (6%)	0·013

Solicited systemic reactions						
Headache	5 (5%)	32 (29%)	<0·0001	7 (6%)	28 (26%)	0·0001
Fatigue	4 (4%)	32 (29%)	<0·0001	4 (4%)	24 (22%)	<0·0001
Feverish	7 (8%)	28 (25%)	0·0012	1 (1%)	22 (20%)	<0·0001
Fever (≥38°C)	0	12 (11%)	0·0013	0	11 (10%)	0·0006
Chills	4 (4%)	18 (16%)	0·0078	1 (1%)	22 (20%)	<0·0001
Drowsiness	5 (5%)	24 (21%)	0·0013	2 (2%)	21 (19%)	<0·0001
Myalgia	3 (3%)	21 (19%)	0·0007	4 (4%)	17 (16%)	0·0023
Malaise	2 (2%)	13 (12%)	0·011	1 (1%)	15 (14%)	0·0002
Pain at the injection site	2 (2%)	10 (9%)	0·043	1 (1%)	13 (12%)	0·0008
Arthralgia	3 (3%)	8 (7%)	0·35	2 (2%)	13 (12%)	0·0027
Loss of appetite	0	9 (8%)	0·0047	2 (2%)	10 (9%)	0·015
Abdominal distention	0	4 (4%)	0·13	1 (1%)	8 (7%)	0·018
Nasal congestion	2 (2%)	7 (6%)	0·19	0	7 (6%)	0·0064
Chest discomfort	1 (1%)	7 (6%)	0·077	2 (2%)	7 (6%)	0·099
Dizziness	4 (4%)	12 (11%)	0·097	2 (2%)	7 (6%)	0·099
Runny nose	3 (3%)	13 (12%)	0·029	4 (4%)	6 (6%)	0·53
Sneezing	2 (2%)	7 (6%)	0·19	1 (1%)	6 (6%)	0·063
Phlegm	4 (4%)	4 (4%)	1·0	1 (1%)	6 (6%)	0·063
Cough	3 (3%)	12 (11%)	0·045	1 (1%)	5 (5%)	0·12
Sore throat	3 (3%)	10 (9%)	0·10	3 (3%)	5 (5%)	0·49
Nausea	1 (1%)	5 (4%)	0·23	4 (4%)	5 (5%)	0·75
Insomnia	2 (2%)	8 (7%)	0·19	1 (1%)	4 (4%)	0·21
Diarrhoea	0	6 (5%)	0·034	3 (3%)	3 (3%)	1·0
Body itching	2 (2%)	6 (5%)	0·30	1 (1%)	3 (3%)	0·36
Flushing of the face	2 (2%)	4 (4%)	0·69	0	3 (3%)	0·12
Chest pain	1 (1%)	2 (2%)	1·0	1 (1%)	3 (3%)	0·36
Abdominal pain	0	1 (1%)	1·0	2 (2%)	3 (3%)	0·68
Palpitations	0	9 (8%)	0·0047	3 (3%)	2 (2%)	1·0000
Skin rash	0	6 (5%)	0·034	0	2 (2%)	0·24
Constipation	2 (2%)	5 (4%)	0·46	1 (1%)	2 (2%)	0·62
Muscle spasms	0	0	··	0	2 (2%)	0·24
Ageusia	0	1 (1%)	1·0	0	1 (1%)	0·49
Enlarged lymph nodes	0	0	··	0	1 (1%)	0·49
Face swelling	0	0	··	0	1 (1%)	0·49
Arm or leg swelling	0	0	··	2 (2%)	1 (1%)	1·0
Vomiting	0	0	··	2 (2%)	1 (1%)	1·0
Facial drooping or weakness	0	0	··	0	1 (1%)	0·49
Nosebleeds	1 (1%)	2 (2%)	1·0	0	0	··
Dyspnoea	0	2 (2%)	0·50	0	0	··
Confusion	0	1 (1%)	1·0	0	0	··
Anosmia	0	0	··	0	0	··
Conjunctivitis	0	0	··	0	0	··
Loss of consciousness	0	0	··	0	0	··
Seizure	0	0	··	0	0	··

Data are n (%), mean (SD), or median (IQR). The four study groups included participants who previously received two doses of CoronaVac and were randomly assigned to receive a third dose of CoronaVac (CC-C) or BNT162b2 (CC-B), and participants who previously received two doses of BNT162b2 and were randomly assigned to receive a third dose of CoronaVac (BB-C) or BNT162b2 (BB-B). Data from 424 (94%) of 451 participants who reported adverse reactions for at least 7 days post vaccination were included. According to the stratified randomisation, comparison was made between CC-C and CC-B groups, and separately between BB-C and BB-B groups. No participants in the CC-C and BB-C groups reported fever.

## Data Availability

The individual participant data and data dictionary that underlie the results reported in this Article will be shared after de-identification (ie, text, tables, figures, and appendices). Anonymised individual participant data, the data dictionary, and other supporting clinical documents (eg, study protocol and questionnaires) will be made available with publication to collaborators at local or international universities or research institutes. Proposals for request to collaborate and data access should be directed to BJC (bowling@hku.hk) or NHLL (leungnan@hku.hk), and will be reviewed and approved by BJC and NHLL on the basis of scientific merit. Data requesters might be requested to sign a data access agreement before gaining access to the data.

## Research in context

### Evidence before this study

Many low-income and middle-income countries use inactivated COVID-19 vaccines in their existing vaccination programmes, and the use of these vaccines has been proposed in high-income countries; however, data comparing inactivated vaccine efficacy with other vaccine platforms are scarce. We searched PubMed and Web of Science for publications between Jan 1, 2019, and June 22, 2023, with the following search strategy and no restriction on language nor publication type: “((covid) OR (sars-cov-2)) AND (((inactivated vaccine) OR (coronavac) OR (sinovac) OR (BIBP) OR (BBIBP) OR (sinopharm)) AND ((booster) OR (third)) AND (((randomised) OR (randomized)) AND (trial)))”. Included within the 55 identified articles were six randomised trials on heterologous COVID-19 vaccination using inactivated vaccines and mRNA vaccines, including three that studied homologous versus heterologous third-dose (booster) vaccination and included live virus neutralising antibodies as primary or secondary outcomes. These three studies compared third-dose heterologous mRNA vaccination to homologous inactivated vaccination after two doses (primary series) of inactivated vaccines, and suggested third-dose heterologous mRNA vaccination led to higher neutralising antibody response. Only one of the studies also included an efficacy outcome (ie, infection), and showed a relative vaccine efficacy of 38% against symptomatic clinical cases with lower risk of infection in the heterologous mRNA vaccination group. None of the six identified randomised trials included comparisons between third-dose mRNA and inactivated vaccine after a primary series of mRNA vaccination.

### Added value of this study

We conducted a comprehensive comparative assessment of homologous versus heterologous COVID-19 vaccination using inactivated vaccines and mRNA vaccines, after both primary series of inactivated vaccines or mRNA vaccines, and simultaneously included safety (adverse events and hospitalisations), immunogenicity (live virus neutralising antibody response and cell-mediated response), and efficacy (breakthrough infections) outcomes. Our results suggest immune benefit, through antibody-mediated or cell-mediated response, or a combination of these responses, in both administering a homologous or heterologous third dose after two doses of inactivated or mRNA vaccination. All four combinations provide similar levels of protection against infection, even in individuals receiving homologous third-dose inactivated vaccine who have low levels of neutralising antibodies against the (mismatched) circulating strain. This finding suggests that neutralising antibody titres might not be the dominant correlate of protection for inactivated vaccines.

### Implications of all the available evidence

Our findings support the benefit of homologous and heterologous booster doses after a primary series of inactivated and mRNA vaccines. All available evidence on vaccine efficacy and effectiveness of inactivated vaccines against variants, compared with the weak neutralising antibody responses conferred by these vaccines, suggest that neutralising antibody levels might not be a sufficient proxy of vaccine efficacy (ie, correlate of protection) in immunogenicity trials or immunobridging studies for inactivated vaccines. Further research is needed to identify how different SARS-CoV-2 vaccine platforms might provide protection via different immune mechanisms. There is an urgent need to identify a correlate of protection for inactivated COVID-19 vaccines, or correlates that might provide information on breadth of protection for all vaccine platforms.
