# Supplementary material for: Comparative antibody and cell-mediated immune responses, reactogenicity, and efficacy of homologous and heterologous boosting with CoronaVac and BNT162b2 (Cobovax): an open-label, randomised trial
Source: Lancet Microbe. Author manuscript; Available in PMC 2023 Sep 27. (PMC10528748; doi:10.1016/S2666-5247(23)00216-1)
Supplement: 1 [file NIHMS1928583-supplement-1.pdf]

THE LANCET
Microbe

Supplementary appendix 1

This translation in Chinese was submitted by the authors and we reproduce it as supplied. It has not been peer reviewed. *The Lancet*'s editorial processes have only been applied to the original in English, which should serve as reference for this manuscript.

此简体中文译文由作者提交，我方按照提供的版本刊登。此译文并未经过同行审阅。医学期刊《柳叶刀》的编辑流程仅适用于英文原稿，英文原稿应作为此手稿的参考。

Supplement to: Leung NHL, Cheng SMS, Cohen CA, et al. Comparative antibody and cell-mediated immune responses, reactogenicity, and efficacy of homologous and heterologous boosting with CoronaVac and BNT162b2 (Cobovax): an open-label, randomised trial. *Lancet Microbe* 2023; published online Aug 4. https://doi.org/10.1016/S2666-5247(23)00216-1.

同源和異源接種克爾來福或復必泰新冠疫苗加強劑的抗體及細胞免疫原性、安全性和有效性對比(互苗研究計劃)：一項隨機、開放標籤臨床試驗

梁曉灡 [1,2,3], 鄭武雙 [1], Carolyn A. Cohen[4], Mario Martín-Sánchez[1], 區艷雯 [1], 陸朗希 [1], 曾志恒 [1], 關啟衡 [1], 黃琬茹 [1], 馮穎釗 [1], 張煒麟 [4], 陳澤佳 [1], 李嘉智 [1], 吳宛 [1], Prathanporn Kaewpreedee[4], 賈志榮 [4], 葉啟明 [1], 潘烈文 [1,4,5], 梁卓偉 [1,3], 裴偉士 [1,4,5], Sophie A. Valkenburg[4,6], 高本恩 [1,3]

1. 香港大學公共衛生學院世衛傳染病流行病學及控制合作中心 2. 美國哈佛大學哈佛陳曾熙公共衛生學院武見計劃(Takemi Program in International Health) 3. 香港科學園醫衛大數據深析實驗室 4. 香港大學公共衛生學院香港大學-巴斯德研究中心 5. 香港科學園免疫與感染研究中心 6. 澳洲墨爾本大學 Peter Doherty Institute of Infection and Immunity

摘要

背景： 就 2019 冠狀病毒病(COVID-19/新冠)，現今只有少數臨床試驗比較同類(同源)和混合(異源)滅活疫苗或 mRNA 疫苗加強劑接種方案。本研究旨在評估同源或異源接種第三劑克爾來福(CoronaVac；科興控股；滅活疫苗)或復必泰(BNT162b2；復星醫藥-BioNTech；mRNA 疫苗)新冠疫苗後的免疫原性、安全性和減低新冠病毒(SARS-CoV-2)感染效用。

方法： 本研究是一項進行中的隨機、隱蔽分組、開放標籤、活性對照試驗，在香港招募年齡 18 歲或以上、來自社區、並於至少 6 個月前已接種兩劑克爾來福(C)或復必泰(B)疫苗的成年人參與。參加者按早前已接種新冠疫苗類型(同源接種兩劑克爾來福或復必泰)分成兩組，分別按由電腦隨機生成順序以 1:1 比例及隱蔽分組被隨機分配接種第三劑克爾來福或復必泰疫苗(原始病毒株)。接種疫苗後，參加者解盲被分配疫苗名稱。主要終點包括接種疫苗後第 28 天血清中針對原始病毒株蝕斑減少 50%中和抗體幾何平均滴度($PRNT_{50}$ GMT)，另測量替代病毒中和抗體平均抑制率(sVNT mean inhibition percentage)、及針對變異株 Omicron 亞型 BA.1 和 BA.2 $PRNT_{50}$。次要終點包括抗體幾何平均增加倍數(GMFR)；局部和全身不良反應發生率；第 7 和 28 天 $IFN\gamma^+CD4^+$和 $IFN\gamma^+CD8^+$T 細胞反應；及 COVID-19 感染率。在符合方案組(per-protocol)，即按已接種第三劑疫苗分類，通過配對樣本 t 檢驗進行組內免疫原性與基線比較，通過獨立樣本 t 檢驗分析兩組間免疫原性差異，通過 Kaplan-Meier 曲線估算感染率及比例風險模型估算兩組間風險比(hazard ratio)。本試驗已在 clinicaltrials.gov 註冊(登記號：NCT05057169)。

結果： 2021 年 11 月 12 日至 2022 年 1 月 27 日期間招募參加者。已接種兩劑克爾來福疫苗組入組 219 名參加者，其中 101 名被隨機分配接種第三劑克爾來福疫苗(CC-C 組)，另 118 名分配接種復必泰疫苗(CC-B 組)；已接種兩劑復必泰疫苗組入組 232 名參加者，其中 118 名被隨機分配接種第三劑克爾來福疫苗(BB-C 組)，另 114 名分配接種復必泰疫苗(BB-B 組)。第 28 天 $PRNT_{50}$ GMT，對原始病毒株分別是 CC-C 組：109，CC-B 組：905，BB-C 組：92，BB-B 組：816；對 Omicron BA.1 變異株分別是 CC-C 組：9，CC-B 組：75，BB-C 組：8，BB-B 組：86；對 Omicron BA.2 變異株分別是 CC-C 組：6，CC-B 組：80，BB-C 組：6，BB-B 組：67。第 28 天 sVNT 平均抑制率，對原始病毒株分別是 CC-C 組：83%，CC-B 組：96%，BB-C 組：87%，BB-B 組：96%；對 Omicron BA.1 變異株分別是 CC-C 組：15%，CC-B 組：58%，BB-C 組：19%，BB-B 組：69%；對 Omicron BA.2 變異株分別是 CC-C 組：43%，CC-B 組：85%，BB-C 組：50%，BB-B 組：90%。已接種兩劑克爾來福疫苗組中，與第三劑克爾來福接種者相比，第三劑復必泰接種者 $PRNT_{50}$ GMFR 是 12($p<0.0001$)；同樣地已接種兩劑復必泰疫苗組中，第三劑復必泰接種者 $PRNT_{50}$ GMFR 是 8($p<0.0001$)。各組間 $CD4^+$ 和 $CD8^+$ T 細胞反應未見差異。在接種第三劑疫苗後一個月內未有疫苗相關住院報告。在 Omicron BA.2 變異株為社區主要傳播株期間，58 名參加者報告 COVID-19 感染，CC-C 組和 CC-B 組感染率分別是 15.3%和 15.4%($p=0.93$)，BB-C 組和 BB-B 組感染率分別是 16.7%和 14.0%($p=0.56$)。

闡釋：各組間 Omicron BA.2 變異株感染率相似，儘管克爾來福第三劑接種者對 Omicron BA.2 變異株的抗體反應非常微弱。需要更多研究尋找適合用於評估新冠滅活疫苗有效性的保護性免疫提代指標。

資助：香港醫療衛生研究基金
