# Supplementary material for: Comparative antibody and cell-mediated immune responses, reactogenicity, and efficacy of homologous and heterologous boosting with CoronaVac and BNT162b2 (Cobovax): an open-label, randomised trial
Source: Lancet Microbe. Author manuscript; Available in PMC 2023 Sep 27. (PMC10528748; doi:10.1016/S2666-5247(23)00216-1)
Supplement: 2 [file NIHMS1928583-supplement-2.pdf]

THE LANCET
Microbe

Supplementary appendix 2

This appendix formed part of the original submission and has been peer reviewed. We post it as supplied by the authors.

Supplement to: Leung NHL, Cheng SMS, Cohen CA, et al. Comparative antibody and cell-mediated immune responses, reactogenicity, and efficacy of homologous and heterologous boosting with CoronaVac and BNT162b2 (Cobovax): an open-label, randomised trial. *Lancet Microbe* 2023; published online Aug 4. https://doi.org/10.1016/S2666-5247(23)00216-1.

Supplementary Information

Comparative antibody and cell-mediated immune responses, reactogenicity and efficacy of homologous and heterologous boosting with CoronaVac and BNT162b2 (Cobovax): an open-label, randomized trial

Nancy H. L. Leung, Samuel M. S. Cheng, Carolyn A. Cohen, Mario Martín-Sánchez, Niki Y. M. Au, Leo L. H. Luk, Leo C. H. Tsang, Kelvin K. H. Kwan, Sara Chaothai, Lison W. C. Fung, Alan W. L. Cheung, Karl C. K. Chan, John K. C. Li, Yvonne Y. Ng, Prathanporn Kaewpreedee, Janice Z. Jia, Dennis K. M. Ip, Leo L. M. Poon, Gabriel M. Leung, J. S. Malik Peiris, Sophie A. Valkenburg, Benjamin J. Cowling

Table of Contents

Supplementary Methods 3

Inclusion and exclusion criteria 3

Participant enrolment and follow-up 3

Sample collection, transport and laboratory analysis 5

Additional references 7

Supplementary Tables 8

Table S1. Characteristics of 435 trial participants at baseline, whom paired Day 0 and 28 sera were tested by ELISA and surrogate virus neutralization test (sVNT). 8

Table S2. Characteristics of randomly selected trial participants at baseline, who were tested by plaque reduction neutralization test (PRNT) to evaluate neutralising antibodies, or who were tested by intracellular cytokine staining (ICS) to evaluate T cell response. 10

Table S3. Reasons for seeking ambulatory care and hospitalization after randomised third-dose CoronaVac or BNT162b2 vaccination 13

Table S4. Change in antibody and cell-mediated response from baseline after randomized third-dose CoronaVac or BNT162b2 vaccination 14

Table S5. Comparisons of antibody and cell-mediated response between randomised third-dose CoronaVac or BNT162b2 vaccination in a random subset of 20 participants from each study arm. 19

Table S6. Comparisons of neutralizing antibodies against Omicron BA.2 subvariant on Day 28 between randomised third-dose CoronaVac or BNT162b2 vaccination in all participants. 23

Table S7. Sensitivity analysis for the association between neutralizing antibodies 28 days post-vaccination and randomised third-dose CoronaVac or BNT162b2 vaccination. 24

Table S8. Proportions of participants who were responders of cell-mediated response at baseline (Day 0), Day 7 and Day 28 after randomised third-dose CoronaVac or BNT162b2 vaccination. 25

Table S9. (A) Participant characteristics and (B) antibody and cell-mediated responses of all vaccinated participants at baseline, stratified by prior two-dose CoronaVac or BNT162b2. 26

Table S10. List of trial participants with SARS-CoV-2 infection and their method of identification. 28

Supplementary Figures 30

Figure S1. A detailed flow chart of participant enrolment for this open-label randomised trial of third-dose CoronaVac or BNT162b2 (Cobovax study), including reasons for exclusion at each stage. 30

Figure S2. Solicited local and systemic reactions during the 7 days after randomised third-dose CoronaVac or BNT162b2 vaccination 34

Figure S3. Serum neutralising antibodies measured by live virus plaque reduction neutralization test (PRNT) against (A) ancestral SARS-CoV-2 virus, (B) Omicron BA.1 and (C) Omicron BA.2 variants, or by (D-F) surrogate virus neutralization test (sVNT) respectively, at baseline and 28 days after randomised third-dose CoronaVac or BNT162b2 vaccination, in the random subset of 20 participants from each study arm. *35*

Figure S4. Fold-change of boosting of (A) neutralising antibody response and (B) cell-mediated response from baseline after randomised third-dose CoronaVac or BNT162b2 vaccination, in random subsets of 20 participants from each study arm. *36*

Figure S5. Correlation between surrogate virus neutralization test (sVNT) and live virus plaque reduction neutralization test (PRNT), for serum neutralizing antibodies against ancestral SARS-CoV-2 virus, Omicron BA.1 and BA.2 virus 28 days after randomised third-dose CoronaVac or BNT162b2 vaccination. *37*

Figure S6. Serum antibodies against ancestral SARS-CoV-2 virus at baseline and Day 28 after randomised third-dose CoronaVac or BNT162b2 vaccination, measured by (A) ELISA, (B) surrogate virus neutralization test (sVNT) or (C) live virus plaque reduction neutralization test (PRNT). *38*

Figure S7. Serum neutralising antibodies against (A-C) ancestral SARS-CoV-2 virus, (D-F) Omicron BA.1 and (G-I) Omicron BA.2 variants at baseline (Day 0) and Day 28 after randomised third-dose CoronaVac or BNT162b2 vaccination, measured by live virus plaque reduction neutralization test (PRNT) with endpoints at 50% (PRNT50), 80% (PRNT80) and 90% (PRNT90) respectively. *39*

Figure S8. IL-4 producing $CD4^+$ T cells at baseline, Day 7 and Day 28 after randomised third-dose CoronaVac or BNT162b2 vaccination *40*

Figure S9. FACS gating strategy for intra cellular cytokine staining (ICS) *41*

Figure S10. Representative FACS plots of IFNγ production by $CD4^+$ and $CD8^+$ T cells, and IL4 production by $CD4^+$ T cells, at baseline (Day 0), Day 7 and Day 28 after randomised third-dose CoronaVac or BNT162b2 vaccination, of (A) responders and (B) non-responders to SARS-CoV-2 structural proteins stimulation, and of (C) buffy coat samples from healthy donors to control stimulations. *42*

Figure S11. Poly-cytokine production (TNFx and IL-2) and memory phenotype in IFNγ-producing (A) CD4+ and (B) CD8+ T cell responses against structural peptides of ancestral SARS-CoV-2 virus, at baseline, 7, and 28 days after randomised third-dose CoronaVac or BNT162b2 vaccination. *44*

Study Protocol **45**

Supplementary Methods

Inclusion and exclusion criteria

Potential participants were eligible for this study if they meet the following inclusion criteria and meet none of the exclusion criteria, assessed before vaccination:

Inclusion criteria

1. Aged 18 years or older at enrolment.
2. Have received two doses of BNT162b2 OR two doses of CoronaVac, with the most recent dose at least six months (180 days) prior to enrolment.
3. Currently resident and planning to remain resident in Hong Kong during the duration of the study, i.e. for 12 months after enrolment.
4. Willing to provide blood samples for all the required time points.
5. The individual or their caregiver have a home phone or cellular or mobile phone for communications purpose.
6. Capable of providing informed consent.

Exclusion criteria

1. A history of laboratory-confirmed or clinically confirmed COVID-19 infection.
2. Have received (at least one dose of) any COVID-19 vaccines other than BNT162b2 or CoronaVac. **[Note 1]**
3. The two vaccine doses in the primary series were 43 days or more apart.
4. Individuals who report any medical condition, or as determined by a clinician, not suitable to receive mRNA or inactivated COVID-19 vaccines, including but not limited to allergies to the active substance or other ingredients of the vaccine. **[Note 2]**
5. Have any medical conditions related to their immune system as determined by a clinician.
6. Use of medication that impairs immune system in the last 6 months, except topical steroids or short-term oral steroids (course lasting ≤ 14 days).
7. Have used immunoglobulins and/or any blood products within 90 days prior to enrolment (administration of the study vaccine).
8. Currently pregnant, or planning, lactation or intention to become pregnant in the coming 3 months. **[Note 3]**

Note: [1] COVID-19 vaccines other than BNT162b2 or CoronaVac include (but not limited to) mRNA-1273 (mRNA vaccine, Moderna), BIBP (inactivated vaccine, Sinopharm), AZD1222 (adenovirus vector-based vaccine, Oxford/AstraZeneca), Sputnik V (adenovirus vector-based vaccine, Gamaleya Research Institute), Ad26.COV2.S (adenovirus vector-based vaccine, Johnson & Johnson) and ZyCoV-D (DNA vaccine, Zydus Cadila). **[2]** mRNA COVID-19 vaccine ingredients include ALC-0315, ALC-0159, DSPC, cholesterol, potassium chloride, potassium dihydrogen phosphate, sodium chloride, disodium phosphate dihydrate, sucrose, water for injection. Inactivated COVID-19 vaccine ingredients include inactivated SARS-CoV-2 virus, aluminium hydroxide, disodium hydrogen phosphate dodecahydrate, sodium dihydrogen phosphate monohydrate and sodium chloride. **[3]** At enrolment we requested enrolled female participants to report to us if they become pregnant during the study, and these participants would be withdrawn upon the reporting.

Participant enrolment and follow-up

The flow of participant enrolment, i.e., various stages of eligibility assessment, randomization, vaccination and selection for laboratory testing is described in **Figure S1**. Invitations to

participate were extended to community-dwelling adults in Hong Kong through mass promotion efforts including advertisements in newspapers, transportation and social media platforms, mass mailing to residential estates, and invitation to and referrals from members of existing cohorts. Interested individuals completed an online application form or called the study hotline. After completing a standardized screening form to preliminarily screen for eligibility and classification into two groups based on prior receipt of two doses of CoronaVac or BNT162b2, the study was explained in detail and participants expressing willingness to participate were randomized to receive either CoronaVac or BNT162b2 (as the third dose) in a 1:1 ratio and assigned a vaccination appointment accordingly. Shortly before or on arrival at the vaccination appointment, we explained the study again, confirmed eligibility and obtained written informed consent, before administering the study vaccination.

At the vaccination appointment (Day 0), all eligible participants were invited to a designated community vaccination centre, which runs under the government's mass vaccination program and offers COVID-19 vaccination to the general public. Under the supervision of the research team, participants were asked to complete a standardised enrolment questionnaire which included information including demographics, medication use, vaccination history, medical conditions and other health-related information such as smoking status, general functional and health status, and socioeconomic status. We collected a 20ml blood sample from the participants, and then unblinded nurses from the community vaccination centres administered the allocated third-dose vaccine intramuscularly. Participants were asked to return on Day 28 for a blood draw and follow-up assessment. Further follow-up blood draws are scheduled at 182 days and 365 days after vaccination. All enrolled participants were also invited to provide additional 10ml whole blood samples at day 0, 7 and 28 for analysis of cell-mediated immune responses (CMI), but only a subset of approximately 35% of participants agreed ("CMI group"). At each encounter for a blood draw we offered a HKD100 (USD13) incentive in the form of a supermarket gift voucher.

After vaccination, participants were provided with vaccination cards and tympanic thermometers, observed for 15-30 minutes for immediate events, and then provided with an e-diary and requested to record daily on possible (delayed) adverse events or symptoms for at least 7 days, or until all adverse events disappeared. In the e-diary, participants were first asked whether they have felt unwell at any time during the past 24 hours. In those who felt unwell, they were asked for the presence of a list of provided (solicited) local and systemic adverse events, and any other (unsolicited) symptoms. Unwell participants were also asked to rate whether the reported adverse events have interfered with usual activities (mild - symptoms easily tolerated and did not interfere usual activities; moderate - symptoms interfered but did not prevent usual activities; severe - symptoms were severe and prevented usual activities), on the use of over-the-counter medication for symptom relief, absent from work or school, seeking medical care and hospitalisation. Participants were also asked for any medical care including ambulatory care and hospitalisation after the third-dose vaccination on Day 28, 182 and 365.

From March 2022, all participants were invited to participate in active surveillance to identify COVID-19 and influenza virus infection. Before initiation, consented participants were first asked about whether they had done any rapid antigen test (RAT) or virologic (PCR) test for COVID-19 or influenza before, and if yes whether there was any past COVID-19 or influenza virus infection defined as a positive test result identified by a RAT or virologic test. Then during active surveillance, all consented participants were provided with complementary COVID-19 RAT kits and asked to test themselves every 4 days ("systematic monitoring"). For

each COVID-19 RAT, participants were asked to report and upload a photo of the test result to the study team through an online questionnaire whether the test result was positive or not. Participants were also asked to report any other RAT or virologic (PCR) test results for COVID-19 or influenza, such as those identified via mandatory mass testing requested by the government or voluntary testing for work purposes; or if any symptoms appear. When a positive test result for COVID-19 or influenza was identified, or any symptoms had appeared regardless of the RAT result, participants were asked to complete an online symptom diary daily, to monitor disease severity, medication use and medical attention ("illness episode"). Starting from 4 days after the initiation of the symptom diary, participants were also asked to performed COVID-19 RAT daily until obtaining negative result for two consecutive days. Participants completed the symptom diary daily for at least 7 days, or until all symptoms disappeared with RAT negative for two consecutive days, whichever the later. All COVID-19 RAT positive result identified during systematic monitoring and illness episodes were verified with the uploaded photo whenever available. The active surveillance is continuing with information collected up to May 31 included in the present analyses.

Sample collection, transport and laboratory analysis
Blood samples were delivered to our study laboratory within 24h of collection, with clotted blood samples kept at 2-8°C and whole blood at room temperature. Sera were kept extracted from the clotted blood samples within 48 hours after collection, divided into 2-4 aliquots, and then stored at -80°C until subsequent serologic testing. Peripheral blood mononuclear cells (PBMCs) were isolated from the heparinized (whole) blood within 24 hours of collection using Ficoll-Paque and leucosep tubes, and cryopreserved in liquid nitrogen until further analysis. For the T cell assays, buffy coat samples from separate healthy blood donors collected in 2020 or earlier, i.e. uninfected and unvaccinated for COVID-19, were also used as internal experimental control. The collection of these buffy coat samples was approved by the Institutional Review Board of the Hong Kong University and Hong Kong Island West Cluster of Hospitals (UW16-254).

For testing of humoral immune responses, we performed an in-house enzyme-linked immunosorbent assay (ELISA) for the receptor binding domain (RBD) of the spike protein, a surrogate virus neutralisation test (sVNT) (GenScript) and a plaque reduction neutralisation test (PRNT), which have been described in our earlier studies.[5,9,10] We have demonstrated a good correlation (r=0.77) between $PRNT_{50}$ and sVNT neutralization percentage for ancestral virus in our earlier studies.[1] The ELISA has a dynamic range of between 0 to 5, although it was not designed as a quantitative assay. For ELISA a single 1:100 serum dilution and for sVNT a single 1:10 serum dilution was tested respectively. sVNT measured negative neutralization percentages were transformed to zero. For PRNT, a serial two-fold serum dilutions from 1:10 to 1:320 were tested initially, and in participants with an initial $PRNT_{50}$ titer of ≥320, additional PRNT using a serial two-fold serum dilutions from 1:40 to 1:1280 were performed. PRNT assays were carried out using ancestral SARS-CoV-2 BetaCoV/Hong Kong/VM20001061/2020 (GISAID EPI_ISL_412028) isolated in Vero-E6 cells (ATCC CRL-1586), the Pango lineage B.1.1.529 Omicron BA.1 subvariant designated hCoV-19/Hong Kong/VM21044713_WHP5047-S5/2021 (GISAID EPI_ISL_6716902) and BA.2 subvariant hCoV-19/Hong Kong/VM22000135_HKUVOC0588P2/2022 (GISAID EPI_ISL_9570707) isolated in Vero-E6 TMRSS2 cells (Vero E6 cells overexpressing TMPRSS2, kindly provided by Dr S Matsuyama and colleagues),[2] and the passage level 3 virus aliquots were used. Cells were maintained in DMEM medium supplemented with 10% FBS and 100 U/ml penicillin–streptomycin (all from ThermoFisher Scientific, Waltham, MA, USA). For each serum sample we determined the $PRNT_{50}$ titer (i.e. the highest serum dilution that neutralising ≥50% of input

plaques) and used this for immunogenicity comparisons, in addition in a subset of samples we also determined the $PRNT_{80}$ and $PRNT_{90}$ titers (i.e. the highest serum dilutions neutralising ≥80% and ≥90% of input plaques respectively) for future analyses of correlate of protection and protective level. PRNT titers were taken as the reciprocal of the serum dilution and were interval-censored, e.g. a sample that was able to neutralise virus at a 1:20 dilution but not at a 1:40 dilution was reported as 20 to indicate that the titer was ≥20 and <40.

A subset of 20 participants from each study arm were randomly selected for testing of cell-mediated immune responses. All testing was done in two experiments with each experiment including 10 participants randomly selected from each study arm, and samples collected from different time points from the same participant were tested in parallel in the same experiment. Cryopreserved PBMCs collected on Day 0, 7 and 28 were thawed into RPMI (Invitrogen, San Diego, CA, USA) with Benzonase (Merck, Raway, NJ, USA) and re-stimulated with an overlapping peptide pool representing the SARS-CoV-2 structural proteins (spike, nucleocapsid, envelope and membrane) (300nM) or negative control DMSO (Sigma, Kawasaki, Japan) (1% in RPMI) in two independent experiments. Stimulation mixes included costimulatory molecules CD28 (Biolegend, San Diego, CA, USA) and CD49d (Biolegend, San Diego, CA, USA), and IL-2 (Roche, Basel, Switzerland). Cells were stimulated for a total of 28 hours at 37°C. Golgi Plug (BD, Franklin Lakes, NJ, USA) containing Brefeldin A (1% in PBS), and Golgi Stop (BD, Franklin Lakes, NJ, USA) containing Monensin (0.67% in PBS) was added at 24 hours during stimulation for further 4-hour incubation. For each experiment, buffy coat samples were also included and stimulated by PMA (10 ng/mL, Sigma, Kawasaki, Japan) /Ionomycin (200 ng/mL, Sigma, Kawasaki, Japan) for 6 hours at 37°C in the presence of Brefeldin A and Monensin, as experimental control for antibody staining. The amino acid sequence of the peptide pools was based on βCoV/Hong Kong/VM20001061/2020 strain (GISAID ID: EPI_ISL_412028, Genscript, Piscataway, NJ, USA). Cells were stained with Zombie-NIR (all antibodies from Biolegend, San Diego, CA, USA and clone used) followed by anti-human CD3-PE/Dazzle 594 (UCHT1), CD4-BV605 (RPA-T4), CD8-AlexaFluor700 (SK1), CCR7-PerCP/Cy5.5 (G043H7), PD-1-BV421 (NAT105), CD45RA-APC (HI100), and a dump channel containing CD19-BV510 (HIB19), CD56-BV510 (5.1H11) and CD14-BV510 (M5E2). Following cell permeabilization with Cytofix/cytoperm (BD, Franklin Lakes, NJ, USA), intracellular staining with anti-IFNγ-FITC (4S.B3), IL4-PE (MP4-25D2) and TNFα-BV711 (MAb11), IL2-PECy7 (MQ1-17H12), was carried out before acquisition of samples. Stained cells were acquired via flow cytometry (AttuneNxT, Invitrogen, San Diego, CA, USA) and analysed by FlowJo v10.8.1. At least 14000 $CD4^{+}$ and 3000 $CD8^{+}$ events were analysed per stimulation. All samples met the cell viability cut-off of at least 30% and thus have been included in the analyses. Gating strategy was shown in **Figure S8**. For each sample, the (paired) IFNγ background response from DMSO stimulation was subtracted from the IFNγ peptide response from SARS-CoV-2 peptides stimulation to give the peptide-specific IFNγ response. We defined the threshold specific to that response as the lowest positive value from all samples collected from all days (0/ 7/ 28) (value for CD4+ IFNγ+ T cells: 0.001%, equivalent to 100 per 100,000 cells; CD8+ IFNγ+ T cells: 0.00017%, equivalent to 17 per 100,000 cells; CD4+ IL4+ T cells: 0.000042%, equivalent to 4.2 per 100,000 cells). A sample with a value larger than this threshold was classified as having a positive response, i.e. a "responder", otherwise was classified as "non-responder". We imputed the values for the non-responders with the value of the respective threshold for plotting and calculating the mean response. (Similar procedure was done for the IL4 response.) Furthermore, CD4+ and CD8+ T cell memory phenotypes (CD45RA/CCR7) and cytokine polyfunctional quality (TNFα/IL2) were assessed by gating on IFNγ+ cells for samples which were classified as CD4+ IFNγ+ T cell or CD8+ IFNγ+ T cell

responders with non-responders excluded. Representative FACS plots for responder, non-responders and controls were shown in **Figure S9**.

Additional references

1 Lau, E. H. *et al.* Long-term persistence of SARS-CoV-2 neutralizing antibody responses after infection and estimates of the duration of protection. *EClinicalMedicine* **41**, 101174, doi:10.1016/j.eclinm.2021.101174 (2021).

2 Matsuyama, S. *et al.* Enhanced isolation of SARS-CoV-2 by TMPRSS2-expressing cells. *Proc. Natl. Acad. Sci. U. S. A.* **117**, 7001-7003, doi:10.1073/pnas.2002589117 (2020).

3 Mok, C. K. P. *et al.* Comparison of the immunogenicity of BNT162b2 and CoronaVac COVID-19 vaccines in Hong Kong. *Respirology* **27**, 301-310, doi:10.1111/resp.14191 (2022).

Supplementary Tables

Table S1. Characteristics of 435 trial participants at baseline, whom paired Day 0 and 28 sera were tested by ELISA and surrogate virus neutralization test (sVNT). 435 trial participants who provided paired Day 0 and 28 sera to evaluate antibodies against ancestral virus, Omicron BA.1 and BA.2 subvariants after third-dose vaccination, and whom Day 28 sera were provided within 60 days after third-dose vaccination were included, i.e. the four study arms included participants who previously received two-dose CoronaVac and were randomised to receive a homologous third dose of CoronaVac ("CC-C") or a heterologous third dose of BNT162b2 ("CC-B"), and participants who previously received two-dose BNT162b2 and were randomised to receive a heterologous third dose of CoronaVac ("BB-C") or a homologous third dose of BNT162b2 ("BB-B"). According to the stratified randomisation, comparison was made between CC-C and CC-B arms, and separately between BB-C and BB-B arms. Differences with p-values ≤ 0.05 were highlighted in bold. ELISA: enzyme-linked immunosorbent assay; sVNT: surrogate virus neutralisation test

	**All enrolled and received third dose vaccination**		**Received primary series of CoronaVac (CC)**				**Received primary series of BNT162b2 (BB)**		
			**Randomised to CoronaVac (CC-C)**	**Randomised to BNT162b2 (CC-B)**	***p***		**Randomised to CoronaVac (BB-C)**	**Randomised to BNT162b2 (BB-B)**	***p***
	**(n = 435)**		**(n = 100)**	**(n = 115)**			**(n = 111)**	**(n = 109)**	
	**n (%)**		**n (%)**	**n (%)**			**n (%)**	**n (%)**	
**Female**	213 (49)		46 (46)	63 (55)	0.1250		48 (43)	56 (51)	0.4926
**Male**	222 (51)		54 (54)	52 (45)	0.9227		63 (57)	53 (49)	0.4035
**Age (mean, SD)**	53 (11)		**55 (12)**	**51 (10)**	**0.0140**		54 (10)	53 (12)	0.4150
**Age group (years)**									
<18	0 (0)		0 (0)	0 (0)			0 (0)	0 (0)	
18 - 29	16 (4)		5 (5)	4 (3)			2 (2)	5 (5)	
30 - 39	36 (8)		8 (8)	12 (10)			8 (7)	8 (7)	
40 - 49	95 (22)		13 (13)	33 (29)			24 (22)	25 (23)	
50 - 59	173 (40)		36 (36)	45 (39)			47 (42)	45 (41)	
60 - 69	97 (22)		32 (32)	20 (17)			28 (25)	17 (16)	
70 - 79	18 (4)		6 (6)	1 (1)			2 (2)	9 (8)	
≥80	0 (0)		0 (0)	0 (0)			0 (0)	0 (0)	
**Ethnicity**					0.3643				0.2614
Chinese	429 (99)		99 (99)	114 (99)			109 (98)	107 (98)	
Caucasian	3 (1)		1 (1)	0 (0)			2 (2)	0 (0)	
Japanese	2 (2)		0 (0)	1 (1)			0 (0)	1 (1)	
Latin American	1 (1)		0 (0)	0 (0)			0 (0)	1 (1)	
Pakistani	0 (0)		0 (0)	0 (0)			0 (0)	0 (0)	
**Obesity**					0.9874				0.6584
Underweight	22 (5)		5 (5)	6 (5)			4 (4)	7 (6)	
Normal	183 (42)		45 (45)	50 (43)			48 (43)	40 (37)	
Overweight	93 (21)		20 (20)	22 (19)			25 (23)	26 (24)	

Obese	137 (31)		30 (30)	37 (32)			34 (31)	36 (33)	
**Chronic medical conditions**	-		-	-			-	-	
Any	102 (23)		21 (21)	21 (18)	1.0000		30 (27)	30 (28)	1.0000
Lung disease, including COPD and asthma	3 (1)		2 (2)	0 (0)	0.5000		0 (0)	1 (1)	1.0000
Heart disease	5 (1)		2 (2)	1 (1)	1.0000		2 (2)	0 (0)	0.5000
Hypertension	45 (10)		9 (9)	10 (9)	1.0000		**7 (6)**	**19 (17)**	**0.0290**
Diabetes	13 (3)		6 (6)	2 (2)	0.2891		3 (3)	2 (2)	1.0000
Hypercholesterolemia	27 (6)		7 (7)	3 (3)	0.3438		9 (8)	8 (7)	1.0000
Kidney disease	1 (0)		0 (0)	0 (0)	1.0000		1 (1)	0 (0)	1.0000
Liver disease	7 (2)		1 (1)	2 (2)	1.0000		2 (2)	2 (2)	1.0000
Cancer	6 (1)		3 (3)	2 (2)	1.0000		0 (0)	1 (1)	1.0000
**Days between first and second dose of COVID-19 vaccination (mean, SD)**	26 (4)		29 (2)	29 (2)	0.6260		22 (2)	23 (3)	0.5248
**Days between second and third (study) dose of COVID-19 vaccination (mean, SD)**	227 (29)		229 (26)	231 (27)	0.59335		223 (29)	224 (32)	0.8180
**Smoking**	-		-	-			-	-	
Ever	19 (4)		8 (8)	6 (5)	0.79		2 (2)	3 (3)	1.0000
Current	10 (2)		4 (4)	4 (3)	1.0000		1 (1)	1 (1)	1.0000

Table S2. Characteristics of randomly selected trial participants at baseline, who were tested by plaque reduction neutralization test (PRNT) to evaluate neutralising antibodies, or who were tested by intracellular cytokine staining (ICS) to evaluate T cell response. Two independent sets of 80 trial participants, 20 selected from each study arm, were randomly selected whom paired Day 0 and 28 sera were tested for PRNT to evaluate neutralising antibodies against ancestral virus and Omicron BA.1 and BA.2 subvariants after third-dose vaccination; or separately selected whom Day 0, 7 and 28 PBMCs were tested for ICS to evaluate T cell response against structural peptides of ancestral SARS-CoV-2 virus after third-dose vaccination. The four study arms included participants who previously received two-dose CoronaVac and were randomised to receive a third dose of CoronaVac ("CC-C") or BNT162b2 ("CC-B"), and participants who previously received two-dose BNT162b2 and were randomised to receive a third dose of CoronaVac ("BB-C") or BNT162b2 ("BB-B"). According to the stratified randomisation, comparison was made between CC-C and CC-B arms, and separately between BB-C and BB-B arms. Differences with p-values ≤ 0.05 were highlighted in bold. PRNT: plaque reduction neutralisation test; ICS: intracellular cytokine staining; PBMCs: peripheral blood mononuclear cells.

	**Randomly selected for PRNT**						**Randomly selected for ICS**					
	**Received primary series of CoronaVac (CC)**			**Received primary series of BNT162b2 (BB)**			**Received primary series of CoronaVac (CC)**			**Received primary series of BNT162b2 (BB)**		
	**Randomised to CoronaVac (CC-C)**	**Randomised to BNT162b2 (CC-B)**	***p***	**Randomised to CoronaVac (BB-C)**	**Randomised to BNT162b2 (BB-B)**	***p***	**Randomised to CoronaVac (CC-C)**	**Randomised to BNT162b2 (CC-B)**	***p***	**Randomised to CoronaVac (BB-C)**	**Randomised to BNT162b2 (BB-B)**	***p***
	**(n = 20)**	**(n = 20)**		**(n = 20)**	**(n = 20)**		**(n = 20)**	**(n = 20)**		**(n = 20)**	**(n = 20)**	
	**n (%)**	**n (%)**		**n (%)**	**n (%)**		**n (%)**	**n (%)**		**n (%)**	**n (%)**	
**Female**	8 (40)	11 (55)	0.6476	8 (40)	7 (35)	1.0000	9 (45)	8 (40)	1.0000	6 (30)	13 (65)	0.1671
**Male**	12 (60)	9 (45)	0.6636	12 (60)	13 (65)	1.0000	11 (55)	12 (60)	1.0000	14 (70)	7 (35)	0.1892
**Age (mean, SD)**	59 (10)	55 (8)	0.1840	53 (11)	50 (15)	0.3809	58 (9)	52 (12)	0.0652	57 (11)	50 (12)	0.0847
**Age group (years)**	-	-		-	-		-	-		-	-	
<18	0 (0)	0 (0)	0.3850	0 (0)	0 (0)	0.2351	0 (0)	0 (0)	0.4197	0 (0)	0 (0)	0.1487
18 - 29	0 (0)	0 (0)		1 (5)	2 (10)		0 (0)	0 (0)		1 (5)	1 (5)	
30 - 39	1 (5)	1 (5)		1 (5)	3 (15)		0 (0)	3 (15)		0 (0)	2 (10)	
40 - 49	2 (10)	4 (20)		4 (20)	4 (20)		5 (25)	7 (35)		2 (10)	6 (30)	
50 - 59	7 (35)	10 (50)		9 (45)	7 (35)		6 (30)	4 (20)		9 (45)	7 (35)	
60 - 69	7 (35)	5 (25)		5 (25)	1 (5)		7 (35)	5 (25)		8 (40)	3 (15)	

70 - 79	3 (15)	0 (0)			0 (0)	3 (15)			2 (10)	1 (5)			0 (0)	1 (5)	
≥80	0 (0)	0 (0)			0 (0)	0 (0)			0 (0)	0 (0)			0 (0)	0 (0)	
**Ethnicity**			1.0000				0.3679				1.0000				1.0000
Chinese	19 (95)	20 (100)			19 (95)	19 (95)			20 (100)	20 (100)			20 (100)	20 (100)	
Caucasian	1 (5)	0 (0)			1 (5)	0 (0)			0 (0)	0 (0)			0 (0)	0 (0)	
Japanese	0 (0)	0 (0)			0 (0)	0 (0)			0 (0)	0 (0)			0 (0)	0 (0)	
Latin American	0 (0)	0 (0)			0 (0)	1 (5)			0 (0)	0 (0)			0 (0)	0 (0)	
Pakistani	0 (0)	0 (0)			0 (0)	0 (0)			0 (0)	0 (0)			0 (0)	0 (0)	
**Obesity**			0.4575				0.7870				**0.0081**				0.6495
Underweight	0 (0)	1 (5)			0 (0)	1 (5)			**1 (5)**	**1 (5)**			1 (5)	3 (15)	
Normal	10 (50)	6 (30)			9 (45)	8 (40)			**14 (70)**	**4 (20)**			8 (40)	7 (35)	
Overweight	4 (20)	4 (20)			7 (35)	7 (35)			**2 (10)**	**2 (10)**			5 (25)	3 (15)	
Obese	6 (30)	9 (45)			4 (20)	4 (20)			**3 (15)**	**13 (65)**			6 (30)	7 (35)	
**Chronic medical conditions**	-	-			-	-			-	-			-	-	
Any	2 (10)	4 (20)	0.6875		6 (30)	4 (20)	0.7539		5 (25)	3 (15)	0.7266		6 (30)	5 (25)	1.0000
Lung disease, including COPD and asthma	0 (0)	0 (0)	1.0000		0 (0)	0 (0)	1.0000		2 (10)	0 (0)	0.5000		0 (0)	0 (0)	1.0000
Heart disease	0 (0)	0 (0)	1.0000		0 (0)	0 (0)	1.0000		1 (5)	0 (0)	1.0000		1 (5)	0 (0)	1.0000
Hypertension	1 (5)	2 (10)	1.0000		0 (0)	3 (15)	0.2500		2 (10)	1 (5)	1.0000		0 (0)	4 (20)	0.1250
Diabetes	0 (0)	0 (0)	1.0000		0 (0)	2 (10)	0.5000		1 (5)	0 (0)	1.0000		0 (0)	1 (5)	1.0000
Hypercholesterolemia	1 (5)	1 (5)	1.0000		1 (5)	2 (10)	1.0000		1 (5)	0 (0)	1.0000		2 (10)	2 (10)	1.0000
Kidney disease	0 (0)	0 (0)	1.0000		1 (5)	0 (0)	1.0000		0 (0)	0 (0)	1.0000		1 (5)	0 (0)	1.0000
Liver disease	0 (0)	0 (0)	1.0000		0 (0)	1 (5)	1.0000		0 (0)	0 (0)	1.0000		0 (0)	0 (0)	1.0000
Cancer	1 (5)	0 (0)	1.0000		0 (0)	0 (0)	1.0000		0 (0)	0 (0)	1.0000		0 (0)	0 (0)	1.0000
**Days between first and second dose of COVID-19 vaccination (mean, SD)**	**28 (1)**	**29 (2)**	**0.0440**		22 (2)	23 (2)	0.1855		29 (1)	29 (1)	0.1661		22 (2)	22 (2)	0.4949

**Days between second and third (study) dose of COVID-19 vaccination (mean, SD)**	234 (20)	231 (22)	0.6739		223 (19)	220 (28)	0.6718		228 (17)	230 (17)	0.7201		231 (25)	227 (33)	0.6148
**Smoking**	-	-			-	-			-	-			-	-	
Ever	1 (5)	0 (0)	1.0000		0 (0)	2 (10)	0.5000		0 (0)	0 (0)	1.0000		0 (0)	1 (5)	1.0000
Current	1 (5)	0 (0)	1.0000		0 (0)	0 (0)	1.0000		0 (0)	0 (0)	1.0000		0 (0)	1 (5)	1.0000

Table S3. Reasons for seeking ambulatory care and hospitalization after randomised third-dose CoronaVac or BNT162b2 vaccination. The four study arms included participants who previously received two-dose CoronaVac and were randomised to receive a third dose of CoronaVac ("CC-C") or BNT162b2 ("CC-B"), and participants who previously received two-dose BNT162b2 and were randomised to receive a third dose of CoronaVac ("BB-C") or BNT162b2 ("BB-B"). Reported discomfort associated with vaccination within the week after third-dose vaccination were highlighted in bold.

	**Study arm**	**Days between third-dose vaccination and seeking ambulatory care**	**Type of ambulatory care facility**	**Days between third-dose vaccination and hospitalization**	**Type of hospitalization facility**	**Summary**
1	BB-B	18	Public Western medical practices	18	Public hospital	NOT related (haemorrhoids)
2	BB-C	3	Private Western medical practices	-	-	NOT related (nausea and dizziness)
3	BB-C	5	Public Western medical practices	-	-	NOT related (diarrhoea)
**4**	**CC-B**	**1**	**Other Chinese/ Western medical practices**	-	-	**Discomfort associated with vaccination within the week after vaccination (muscle pain)**
**5**	**CC-B**	**3**	**Private Western medical practices**	-	-	**Discomfort associated with vaccination within the week after vaccination (flu-like symptoms)**
**6**	**CC-B**	**5**	**Private Chinese medical practices**	-	-	**Discomfort associated with vaccination within the week after vaccination (flu-like symptoms & fatigue post-vaccination)**
7	CC-B	15	Public Western medical practices	-	-	NOT related (anaemia, normal ECG)
8	CC-B	27	Public Western medical practices	-	-	NOT related (flu)
**9**	**CC-C**	**7**	**Private Western medical practices**	-	-	**Discomfort associated with vaccination within the week after vaccination (muscle pain)**
10	CC-C	24	Public Western medical practices	-	-	NOT related (flu)

Table S4. Change in antibody and cell-mediated response from baseline after randomized third-dose CoronaVac or BNT162b2 vaccination. (A) Change in antibody response on Day 28 compared to baseline. Change in **(B)** IFNγ-producing $CD4^+$ and $CD8^+$ T cell responses, and **(C)** their poly-cytokine production (TNFα and IL-2) and memory phenotype, on Day 7 and Day 28 compared to baseline**.** Antibody response were evaluated against the ancestral virus, Omicron BA.1 and BA.2 subvariants, while T cell responses were evaluated upon stimulation by overlapping peptide pool representing the SARS-CoV-2 structural proteins (spike, nucleocapsid, envelope and membrane). The four study arms included participants who previously received two-dose CoronaVac and were randomised to receive a third dose of CoronaVac ("CC-C") or BNT162b2 ("CC-B"), and participants who previously received two-dose BNT162b2 and were randomised to receive a third dose of CoronaVac ("BB-C") or BNT162b2 ("BB-B"). Data on ELISA against ancestral virus, and data on sVNT against ancestral virus, Omicron BA.1 and BA.2 variants, on sera collected at baseline and day 28 post-vaccination was obtained from all participants (with paired sera). Data on PRNT against ancestral virus, Omicron BA.1 and BA.2 variants on sera collected at baseline and day 28 from a random subset of 20 participants selected from each study arm was included. Comparison was made between paired baseline (day 0) and day 28 samples collected from each participant. Differences with p-values ≤ 0.05 were highlighted in bold.

Table S4A. Changes in antibody response on Day 28 compared to baseline. *The mean values for $PRNT_{50}$, $PRNT_{80}$ and $PRNT_{90}$ titers were considered as geometric mean titer (GMT), and the fold-change in PRNT titers on Day 28 from baseline were considered as geometric mean fold rise (GMFR). ELISA: enzyme-linked immunosorbent assay; sVNT: surrogate virus neutralisation test; PRNT: plaque reduction neutralisation test; OD: optical density; % inhibition: percent inhibition; $PRNT_{50}$, $PRNT_{80}$ and $PRNT_{90}$: PRNT titers with endpoints at 50%, 80% and 90% inhibition respectively.*

		n		Baseline	Day 28		Baseline vs. Day 28	
				**Mean**	**Mean**		**Fold change**	**p-value**
ELISA OD								
**Ancestral virus**								
CC-C		100		0.38	1.73		**4.5**	**<0.0001**
CC-B		115		0.39	2.43		**6.3**	**<0.0001**
BB-C		111		1.31	1.8		**1.4**	**<0.0001**
BB-B		109		1.35	2.35		**1.7**	**<0.0001**
sVNT % inhibition								
**Ancestral virus**								
CC-C		100		17	83		**5.0**	**<0.0001**
CC-B		115		16	96		**6.1**	**<0.0001**
BB-C		111		66	87		**1.3**	**<0.0001**
BB-B		109		70	96		**1.4**	**<0.0001**
**Omicron BA.1 variant**								
CC-C		100		6	15		**2.5**	**<0.0001**
CC-B		115		6	58		**10.2**	**<0.0001**
BB-C		111		8	19		**2.2**	**<0.0001**
BB-B		109		7	69		**9.6**	**<0.0001**
**Omicron BA.2 variant**								
CC-C		100		5	43		**8.0**	**<0.0001**
CC-B		115		6	85		**13.6**	**<0.0001**
BB-C		111		28	50		**1.8**	**<0.0001**
BB-B		109		29	90		**3.1**	**<0.0001**
$PRNT_{50}$ titer								

**Ancestral virus**								
CC-C		20		8	109		**13.5**	**<0.0001**
CC-B		20		10	905		**93.7**	**<0.0001**
BB-C		20		34	92		**2.7**	**<0.0001**
BB-B		20		43	816		**19.0**	**<0.0001**
**Omicron BA.1 variant**								
CC-C		20		5	9		**1.8**	**0.0011**
CC-B		20		5	75		**14.4**	**<0.0001**
BB-C		20		6	8		**1.5**	**0.0007**
BB-B		20		6	86		**15.5**	**<0.0001**
**Omicron BA.2 variant**								
CC-C		20		6	6		1.1	0.5454
CC-B		20		5	80		**15.5**	**<0.0001**
BB-C		20		5	6		**1.2**	**0.0102**
BB-B		20		5	67		**12.6**	**<0.0001**
$PRNT_{80}$ titer								
**Ancestral virus**								
CC-C		20		5	57		**10.9**	**<0.0001**
CC-B		20		6	520		**84.4**	**<0.0001**
BB-C		20		17	46		**2.7**	**<0.0001**
BB-B		20		21	381		**17.8**	**<0.0001**
**Omicron BA.1 variant**								
CC-C		20		5	6		1.3	0.0308
CC-B		20		5	39		**7.7**	**<0.0001**
BB-C		20		5	5		1.0	0.3299
BB-B		20		5	43		**8.6**	**<0.0001**
**Omicron BA.2 variant**								
CC-C		20		5	6		1.0	0.7481
CC-B		20		5	41		**8.3**	**<0.0001**
BB-C		20		5	5		1.0	1.0000
BB-B		20		5	34		**6.5**	**<0.0001**
$PRNT_{90}$ titer								
**Ancestral virus**								
CC-C		20		5	31		**6.3**	**<0.0001**
CC-B		20		6	331		**59.7**	**<0.0001**
BB-C		20		10	25		**2.5**	**<0.0001**
BB-B		20		12	219		**18.4**	**<0.0001**
**Omicron BA.1 variant**								
CC-C		20		5	6		1.1	0.0828
CC-B		20		5	22		**4.4**	**0.0001**
BB-C		20		5	5		1.0	1.0000
BB-B		20		5	26		**5.3**	**<0.0001**
**Omicron BA.2 variant**								
CC-C		20		5	5		1.0	0.5770
CC-B		20		5	25		**4.9**	**<0.0001**
BB-C		20		5	5		1.0	1.0000
BB-B		20		5	22		**4.4**	**<0.0001**

Table S4B. Changes in IFNγ-producing CD4⁺ and CD8⁺ T cell responses on Day 7 and Day 28 compared to baseline. *The mean values of CD4+ or CD8+ T cells were expressed in number of cells per 100,000 cells.*

	n		Baseline	Day 7	Day 28		Baseline vs. Day 7			Baseline vs. Day 28	
			**Mean**	**Mean**	**Mean**		**Fold change**	***p***		**Fold change**	***p***
**IFNg-producing CD4⁺ cells** (per 100,000)											
CC-C	20		877	4248	2241		**4.8**	**0.0007**		**2.6**	**0.0430**
CC-B	20		641	3203	1822		**5**	**0.0004**		**2.8**	**0.0142**
BB-C	20		3034	2745	2449		0.9	0.8091		0.8	0.5043
BB-B	20		2196	2087	1383		1	0.9241		0.6	0.3724
**IFNg-producing CD8⁺ cells** (per 100,000)											
CC-C	20		208	668	359		3.2	0.1333		1.7	0.5542
CC-B	20		78	969	98		**12.4**	**0.0021**		1.2	0.6329
BB-C	20		213	122	275		0.6	0.4993		1.3	0.6816
BB-B	20		146	173	179		1.2	0.8360		1.2	0.8157
**IL4-producing CD4+ cells** (per 100,000)											
CC-C	20		119	68	84		0.6	0.4301		0.7	0.6440
CC-B	20		98	203	25		1.8	0.3157		**0.3**	**0.0120**
BB-C	20		66	61	65		0.9	0.8987		1.**0**	0.9868
BB-B	20		112	180	64		1.5	0.2190		0.6	0.3405

Table S4C. Changes in poly-cytokine production (TNFα and IL-2) and memory phenotype in IFNγ-producing CD4⁺ and CD8⁺ T cells on Day 7 and Day 28 compared to baseline. *Tn: naïve T cells; Tcm: central memory T cells; Tem: effector memory T cells; Teem: terminal effector memory T cells.*

		n	Baseline	Day 7	Day 28		Baseline vs. Day 7			Baseline vs. Day 28	
			**Mean**	**Mean**	**Mean**		**Difference**	**p-value**		**Difference**	**p-value**
**Cytokine polyfunctional quality**											
CD4⁺ T cells											
**CC-C**		20									
IFNg⁺			45.10%	58.80%	53.30%		13.70%	0.2374		8.20%	0.4654
IFNg⁺ TNFa⁺			11.50%	23.40%	8.10%		11.90%	0.1147		-3.50%	0.5450
IFNg⁺ IL2⁺			2.80%	3.60%	9.30%		0.80%	0.6499		**6.50%**	**0.0248**
IFNg⁺ TNFa⁺ IL2⁺			10.60%	14.20%	9.30%		3.60%	0.4931		-1.30%	0.7916
**CC-B**		20									
IFNg⁺			42.60%	61.70%	61.60%		19.10%	0.0624		19.00%	0.0707
IFNg⁺ TNFa⁺			8.10%	21.40%	9.00%		**13.30%**	**0.0033**		1.00%	0.7328
IFNg⁺ IL2⁺			2.20%	3.10%	3.60%		0.90%	0.4231		1.40%	0.2223
IFNg⁺ TNFa⁺ IL2⁺			7.20%	8.80%	5.90%		1.60%	0.5436		-1.30%	0.5799
**BB-C**		20									
IFNg⁺			51.20%	50.20%	47.20%		-1.00%	0.9036		-4.00%	0.6127
IFNg⁺ TNFa⁺			15.00%	11.10%	10.60%		-3.90%	0.2625		-4.40%	0.1166
IFNg⁺ IL2⁺			5.40%	10.70%	11.60%		**5.40%**	**0.0074**		**6.30%**	**0.0184**
IFNg⁺ TNFa⁺ IL2⁺			18.40%	12.90%	15.60%		-5.40%	0.2626		-2.80%	0.5261

**BB-B**		20									
IFNg+			48.70%	47.20%	50.90%		-1.40%	0.8722		2.20%	0.8258
IFNg+ TNFa+			15.00%	14.20%	9.70%		-0.70%	0.8334		-5.30%	0.1238
IFNg+ IL2+			3.90%	4.30%	6.20%		0.40%	0.8176		2.30%	0.3062
IFNg+ TNFa+ IL2+			12.50%	9.20%	8.20%		-3.20%	0.5071		-4.20%	0.3496
CD8+ T cells											
**CC-C**		20									
IFNg+			49.30%	74.40%	51.60%		25.10%	0.1143		2.30%	0.8891
IFNg+ TNFa+			0.60%	5.50%	3.30%		4.90%	0.0508		**2.70%**	**0.0253**
IFNg+ IL2+			0.00%	0.20%	4.30%		0.20%	0.3299		4.30%	0.1097
IFNg+ TNFa+ IL2+			0.10%	0.00%	0.80%		-0.10%	0.3299		0.70%	0.2530
**CC-B**		20									
IFNg+			27.50%	71.00%	43.20%		**43.50%**	**0.0039**		15.70%	0.2464
IFNg+ TNFa+			0.90%	4.60%	1.60%		3.70%	0.108		0.70%	0.4248
IFNg+ IL2+			1.10%	2.20%	0.20%		1.10%	0.4591		-0.90%	0.1433
IFNg+ TNFa+ IL2+			0.50%	2.20%	0.00%		1.70%	0.314		-0.50%	0.3299
**BB-C**		20									
IFNg+			37.10%	34.30%	61.30%		-2.80%	0.8367		**24.20%**	**0.0356**
IFNg+ TNFa+			4.50%	2.60%	2.80%		-2.00%	0.3949		-1.70%	0.5504
IFNg+ IL2+			2.80%	1.10%	4.50%		-1.70%	0.3537		1.70%	0.5442
IFNg+ TNFa+ IL2+			5.60%	2.10%	1.30%		-3.50%	0.3395		-4.30%	0.2034
**BB-B**		20									
IFNg+			31.90%	41.90%	43.40%		10.00%	0.4218		11.50%	0.4202
IFNg+ TNFa+			6.60%	4.10%	1.90%		-2.50%	0.6755		-4.70%	0.4063
IFNg+ IL2+			0.50%	1.10%	1.00%		0.60%	0.5012		0.50%	0.5824
IFNg+ TNFa+ IL2+			3.10%	0.30%	1.00%		-2.90%	0.2436		-2.10%	0.4702
**Memory phenotype**											
CD4+ T cells											
**CC-C**		20									
Tn			2.20%	6.90%	4.30%		4.70%	0.0514		2.10%	0.3455
Teem			1.90%	4.80%	2.80%		2.90%	0.2294		0.90%	0.6239
Tcm			26.90%	21.20%	19.60%		-5.70%	0.3942		-7.30%	0.2560
Tem			39.10%	67.10%	53.30%		**28.00%**	**0.0007**		14.20%	0.0793
**CC-B**		20									
Tn			6.20%	3.30%	5.20%		-2.90%	0.1156		-1.00%	0.6776
Teem			1.40%	0.90%	3.20%		-0.50%	0.5677		1.80%	0.1741
Tcm			15.30%	26.20%	20.60%		10.90%	0.0310		5.30%	0.2648
Tem			37.10%	64.60%	51.10%		**27.50%**	**0.0014**		14.00%	0.0694
**BB-C**		20									

Tn			7.20%	2.40%	4.50%		-4.80%	0.3618		-2.60%	0.6320
Teem			1.50%	2.00%	3.20%		0.50%	0.6306		1.80%	0.0579
Tcm			25.10%	21.70%	19.60%		-3.40%	0.4249		-5.60%	0.2062
Tem			56.20%	58.90%	57.60%		2.70%	0.7374		1.40%	0.8620
**BB-B**		20									
Tn			3.90%	2.40%	2.60%		-1.50%	0.5881		-1.30%	0.6842
Teem			1.10%	1.40%	2.00%		0.30%	0.7277		0.90%	0.3894
Tcm			24.70%	16.80%	18.80%		-7.90%	0.0482		-5.90%	0.2199
Tem			50.40%	54.50%	51.60%		4.10%	0.6446		1.20%	0.8774
$CD8^+$ T cells											
**CC-C**		20									
Tn			11.50%	14.10%	10.30%		2.60%	0.7200		-1.20%	0.8582
Teem			6.30%	16.50%	9.10%		10.20%	0.1299		2.80%	0.5801
Tcm			7.60%	12.30%	15.20%		4.70%	0.3110		7.50%	0.2360
Tem			24.50%	37.10%	25.40%		12.50%	0.2895		0.80%	0.9414
**CC-B**		20									
Tn			3.40%	10.80%	9.90%		7.40%	0.0930		6.60%	0.2888
Teem			4.50%	9.20%	4.60%		4.60%	0.4442		0.10%	0.9795
Tcm			5.90%	19.10%	11.00%		**13.30%**	**0.0152**		5.10%	0.4206
Tem			16.20%	40.90%	19.50%		**24.70%**	**0.0089**		3.30%	0.6384
**BB-C**		20									
Tn			10.20%	6.20%	14.00%		-4.10%	0.4715		3.80%	0.3871
Teem			7.20%	3.10%	10.90%		-4.10%	0.3106		3.70%	0.5719
Tcm			10.70%	6.60%	12.20%		-4.10%	0.2403		1.50%	0.7043
Tem			21.90%	24.20%	32.90%		2.30%	0.8257		11.00%	0.1948
**BB-B**		20									
Tn			9.60%	4.70%	8.50%		-4.80%	0.2221		-1.10%	0.7676
Teem			3.40%	4.30%	5.80%		0.90%	0.5933		2.40%	0.3296
Tcm			13.20%	10.20%	10.20%		-3.00%	0.6715		-3.00%	0.6788
Tem			16.00%	28.20%	23.00%		12.20%	0.1344		7.00%	0.4232

Table S5. Comparisons of antibody and cell-mediated response between randomised third-dose CoronaVac or BNT162b2 vaccination in a random subset of 20 participants from each study arm. (A) Comparisons of antibody response on Day 28**.** Comparisons of **(B)** IFNγ-producing $CD4^+$ and $CD8^+$ T cell responses, and **(C)** their poly-cytokine production (TNFα and IL-2) and memory phenotype, on Day 7 and 28. The four study arms included participants who previously received two-dose CoronaVac and were randomised to receive a third dose of CoronaVac ("CC-C") or BNT162b2 ("CC-B"), and participants who previously received two-dose BNT162b2 and were randomised to receive a third dose of CoronaVac ("BB-C") or BNT162b2 ("BB-B"). According to the stratified randomisation, comparison was made between CC-C and CC-B arms, and separately between BB-C and BB-B arms. Data on ELISA against ancestral virus, and data on sVNT against ancestral virus, Omicron BA.1 and BA.2 variants, on sera collected at baseline and day 28 post-vaccination from all participants (with paired sera, i.e. CC-C: n=100; CC-B: n=115; BB-C: n=111; BB-B: n=109) was included. Data on PRNT against ancestral virus, Omicron BA.1 and BA.2 variants on sera collected at baseline and day 28 from a random subset of 20 participants selected from each study arm was included. Data on cell-mediated response (CMI) on PBMCs collected at baseline, day 7 and day 28 from a separate random subset of 20 participants selected from each study arm from participants in the CMI group was included. Differences with p-values ≤ 0.05 were highlighted in bold. PBMCs: peripheral blood mononuclear cells.

Table S5A. Comparisons of antibody response on Day 0 and 28 between third dose CoronaVac or BNT162b2. *The mean values for $PRNT_{50}$, $PRNT_{80}$ and $PRNT_{90}$ titers were considered as geometric mean titer (GMT), and the fold-difference in PRNT titers between homologous and heterologous third dose were considered as geometric mean fold rise (GMFR). ELISA: enzyme-linked immunosorbent assay; sVNT: surrogate virus neutralisation test; PRNT: plaque reduction neutralisation test; OD: optical density; % inhibition: percent inhibition; $PRNT_{50}$, $PRNT_{80}$ and $PRNT_{90}$: PRNT titers with endpoints at 50%, 80% and 90% inhibition respectively.*

	**Baseline**					**Day 28**			
	**Third-dose CoronaVac**	**Third-dose BNT162b2**	**Fold-difference**	***p***		**Third-dose CoronaVac**	**Third-dose BNT162b2**	**Fold-difference**	***p***
	**Mean**	**Mean**				**Mean**	**Mean**		
**Prior two-dose CoronaVac**									
**ELISA OD**									
Ancestral virus	0.38	0.39	1	0.8956		1.73	2.43	**1.4**	**<0.0001**
**sVNT % inhibition**									
Ancestral virus	17	16	0.9	0.6755		83	96	**1.2**	**<0.0001**
Omicron BA.1 variant	6	6	0.9	0.5632		15	58	**3.9**	**<0.0001**
Omicron BA.2 variant	5	6	1.2	0.3004		43	85	**2.0**	**<0.0001**
**$PRNT_{50}$ titer**									
Ancestral virus	8	10	1.2	0.3189		109	905	**8.3**	**<0.0001**
Omicron BA.1 variant	5	5	1	>0.9999		9	75	**8.0**	**<0.0001**
Omicron BA.2 variant	6	5	0.9	0.5333		6	80	**13**	**<0.0001**
**$PRNT_{80}$ titer**									
Ancestral virus	5	6	1.2	0.1206		57	520	**9.2**	**<0.0001**

Omicron BA.1 variant	5	5	1	>0.9999		6	39	**6.1**	**<0.0001**
Omicron BA.2 variant	5	5	0.9	0.3299		6	41	**7.5**	**<0.0001**
**$PRNT_{90}$ titer**									
Ancestral virus	5	6	1.1	0.0828		31	331	**10.6**	**<0.0001**
Omicron BA.1 variant	5	5	1	>0.9999		6	22	**4**	**<0.0001**
Omicron BA.2 variant	5	5	1	0.3299		5	25	**4.6**	**<0.0001**
**Prior two-dose BNT162b2**									
**ELISA OD**									
Ancestral virus	1.31	1.35	1	0.4478		1.8	2.35	**1.3**	**<0.0001**
**sVNT % inhibition**									
Ancestral virus	66	70	1.1	0.1143		87	96	**1.1**	**<0.0001**
Omicron BA.1 variant	8	7	0.9	0.2108		19	69	**3.7**	**<0.0001**
Omicron BA.2 variant	28	29	1	0.6575		50	90	**1.8**	**<0.0001**
**$PRNT_{50}$ titer**									
Ancestral virus	34	43	1.3	0.2320		92	816	**8.9**	**<0.0001**
Omicron BA.1 variant	6	6	1	>0.9999		8	86	**10.6**	**<0.0001**
Omicron BA.2 variant	5	5	1.1	0.3299		6	67	**10.9**	**<0.0001**
**$PRNT_{80}$ titer**									
Ancestral virus	17	21	1.3	0.2320		46	381	**8.3**	**<0.0001**
Omicron BA.1 variant	5	5	1	>0.9999		5	43	**8.3**	**<0.0001**
Omicron BA.2 variant	5	5	1	0.3299		5	34	**6.7**	**<0.0001**
**$PRNT_{90}$ titer**									
Ancestral virus	10	12	1.2	0.3609		25	219	**8.9**	**<0.0001**
Omicron BA.1 variant	5	5	1	>0.9999		5	26	**5.3**	**<0.0001**
Omicron BA.2 variant	5	5	1	>0.9999		5	22	**4.4**	**<0.0001**

Table S5B. Comparisons of IFNγ-producing $CD4^+$ and $CD8^+$ T cell responses on Day 0, 7 and 28 between third dose CoronaVac or BNT162b2. *The mean values of CD4+ or CD8+ T cells were expressed in number of cells per 100,000 cells.*

	Baseline				Day 7				Day 28			
	**Third-dose CoronaVac**	**Third-dose BNT162b2**	**Fold-difference**	***p***	**Third-dose CoronaVac**	**Third-dose BNT162b2**	**Fold-difference**	***p***	**Third-dose CoronaVac**	**Third-dose BNT162b2**	**Fold-difference**	***p***
	**Mean**	**Mean**			**Mean**	**Mean**			**Mean**	**Mean**		
**<u>Prior two-dose CoronaVac</u>** <u>(No. of cells per 100,000)</u>												
IFNg-producing $CD4^+$ cells	877	641	0.7	0.584	4248	3203	0.8	0.4608	2241	1822	0.8	0.7213
IFNg-producing $CD8^+$ cells	208	78	0.4	0.2639	668	969	1.5	0.6106	359	98	0.3	0.1113
IL4-producing $CD4^+$ cells	119	98	0.8	0.8312	68	203	3.0	0.2300	84	25	0.3	0.1308
**<u>Prior two-dose BNT162b2</u>** <u>(No. of cells per 100,000)</u>												
IFNg-producing $CD4^+$ cells	3034	2196	0.7	0.5479	2745	2087	0.8	0.6332	2449	1383	0.6	0.2737
IFNg-producing $CD8^+$ cells	213	146	0.7	0.6749	122	173	1.4	0.6769	275	179	0.7	0.5695
IL4-producing $CD4^+$ cells	66	112	1.7	0.5657	61	180	2.9	0.2173	65	64	1	0.9904

Table S5C. Comparisons of poly-cytokine production (TNFα and IL-2) and memory phenotype in IFNγ-producing $CD4^+$ and $CD8^+$ T cells on Day 0, 7 and 28 between third dose CoronaVac or BNT162b2. *Tn: naïve T cells; Tcm: central memory T cells; Tem: effector memory T cells; Teem: terminal effector memory T cells.*

	Baseline				Day 7				Day 28			
	**Third-dose CoronaVac**	**Third-dose BNT162b2**	**Mean-difference**	**p-value**	**Third-dose CoronaVac**	**Third-dose BNT162b2**	**Mean-difference**	**p-value**	**Third-dose CoronaVac**	**Third-dose BNT162b2**	**Mean-difference**	**p-value**
	**Mean**	**Mean**			**Mean**	**Mean**			**Mean**	**Mean**		
**<u>Prior two-dose CoronaVac</u>**												
Cytokine polyfunctional quality												
**$CD4^+$ T cells**												
$IFNg^+$	45.1%	42.6%	-2.5%	0.8444	58.8%	61.7%	2.9%	0.6330	53.3%	61.6%	8.2%	0.4251
$IFNg^+$ $TNFa^+$	11.5%	8.1%	-3.5%	0.5723	23.4%	21.4%	-2.1%	0.6639	8.1%	9.0%	1.0%	0.7261
$IFNg^+$ $IL2^+$	2.8%	2.2%	-0.6%	0.6764	3.6%	3.1%	-0.5%	0.7554	**9.3%**	**3.6%**	**-5.7%**	**0.0263**
$IFNg^+$ $TNFa^+$ $IL2^+$	10.6%	7.2%	-3.4%	0.4984	14.2%	8.8%	-5.4%	0.1316	9.3%	5.9%	-3.5%	0.1713
**$CD8^+$ T cells**												
$IFNg^+$	49.3%	27.5%	-21.8%	0.1517	74.4%	71.0%	-3.4%	0.7843	51.6%	43.2%	-8.4%	0.5735
$IFNg^+$ $TNFa^+$	0.6%	0.9%	0.3%	0.6678	5.5%	4.6%	-0.9%	0.7872	3.3%	1.6%	-1.7%	0.2734
$IFNg^+$ $IL2^+$	0.0%	1.1%	1.1%	0.1385	0.2%	2.2%	2.1%	0.1297	**4.3%**	**0.2%**	**-4.0%**	0.1319

IFNg⁺<br>TNFa⁺<br>IL2⁺	0.1%	0.5%	0.4%	0.4788	0.0%	2.2%	2.2%	0.2141	0.8%	0.0%	-0.8%	0.1641
Memory phenotype												
**CD4⁺ T cells**												
Tn	2.2%	6.2%	4.0%	0.1135	6.9%	3.3%	-3.6%	0.1806	4.3%	5.2%	0.8%	0.6797
Teem	1.9%	1.4%	-0.5%	0.7482	**4.8%**	**0.9%**	**-3.9%**	**0.0500**	2.8%	3.2%	0.4%	0.8198
Tcm	26.9%	15.3%	-11.6%	0.1304	21.2%	26.2%	5.0%	0.1771	19.6%	20.6%	1.0%	0.8285
Tem	39.1%	37.1%	-2.0%	0.8539	67.1%	64.6%	-2.5%	0.6460	53.3%	51.1%	-2.2%	0.8145
**CD8⁺ T cells**												
Tn	11.5%	3.4%	-8.1%	0.1983	14.1%	10.8%	-3.3%	0.6030	10.3%	9.9%	-0.4%	0.9486
Teem	6.3%	4.5%	-1.7%	0.6687	16.5%	9.2%	-7.3%	0.3032	9.1%	4.6%	-4.5%	0.2778
Tcm	7.6%	5.9%	-1.8%	0.6619	12.3%	19.1%	6.8%	0.2997	15.2%	11.0%	-4.2%	0.5983
Tem	24.6%	16.2%	-8.3%	0.4224	37.1%	40.9%	3.8%	0.6941	25.4%	19.5%	-5.9%	0.5182
**Prior two-dose BNT162b2**												
Cytokine polyfunctional quality												
**CD4⁺ T cells**												
IFNg⁺	51.2%	48.7%	-2.6%	0.8000	50.2%	47.2%	-3.0%	0.7442	47.2%	50.9%	3.6%	0.7001
IFNg⁺<br>TNFa⁺	15.0%	15.0%	-0.1%	0.9878	11.1%	14.2%	3.1%	0.3882	10.6%	9.7%	-0.9%	0.7305
IFNg⁺<br>IL2⁺	5.4%	3.9%	-1.5%	0.5649	**10.7%**	**4.3%**	**-6.4%**	**0.0077**	11.6%	6.2%	-5.4%	0.0568
IFNg⁺<br>TNFa⁺<br>IL2⁺	18.4%	12.5%	-5.9%	0.3344	12.9%	9.2%	-3.7%	0.3059	15.6%	8.2%	-7.3%	0.0604
**CD8⁺ T cells**												
IFNg⁺	37.1%	30.3%	-6.8%	0.6195	34.3%	39.8%	5.5%	0.6991	61.3%	41.2%	-20.1%	0.1630
IFNg⁺<br>TNFa⁺	4.5%	6.3%	1.7%	0.7512	2.6%	3.9%	1.3%	0.5936	2.8%	1.8%	-1.0%	0.6343
IFNg⁺<br>IL2⁺	2.8%	0.5%	-2.3%	0.2599	1.1%	1.0%	0.0%	0.9691	4.5%	0.9%	-3.6%	0.0570
IFNg⁺<br>TNFa⁺<br>IL2⁺	5.6%	3.0%	-2.6%	0.5059	2.1%	0.2%	-1.8%	0.2944	1.3%	1.0%	-0.3%	0.7851
Memory phenotype												
**CD4⁺ T cells**												
Tn	7.2%	3.9%	-3.3%	0.5728	2.4%	2.4%	0.0%	0.9804	4.5%	2.6%	-1.9%	0.3671
Teem	1.5%	1.1%	-0.4%	0.5523	2.0%	1.4%	-0.6%	0.523	3.2%	2.0%	-1.2%	0.3473
Tcm	25.1%	24.7%	-0.5%	0.934	21.7%	16.8%	-5.0%	0.2475	19.6%	18.8%	-0.7%	0.8694
Tem	56.2%	50.4%	-5.8%	0.5211	58.9%	54.5%	-4.4%	0.652	57.6%	51.6%	-6.1%	0.5391
**CD8⁺ T cells**												
Tn	10.2%	9.1%	-1.1%	0.8404	6.2%	4.5%	-1.7%	0.7095	14.0%	8.1%	-6.0%	0.3140
Teem	7.2%	3.2%	-4.0%	0.2977	3.1%	4.1%	1.0%	0.6767	10.9%	5.5%	-5.4%	0.3398
Tcm	10.7%	12.5%	1.9%	0.792	6.6%	9.7%	3.1%	0.467	12.2%	9.7%	-2.5%	0.5841
Tem	21.9%	15.2%	-6.7%	0.4409	24.2%	26.8%	2.6%	0.8006	32.9%	21.8%	-11.1%	0.2589

Table S6. Comparisons of neutralizing antibodies against Omicron BA.2 subvariant on Day 28 between randomised third-dose CoronaVac or BNT162b2 vaccination in all participants. The four study arms included participants who previously received two-dose CoronaVac and were randomised to receive a third dose of CoronaVac ("CC-C") or BNT162b2 ("CC-B"), and participants who previously received two-dose BNT162b2 and were randomised to receive a third dose of CoronaVac ("BB-C") or BNT162b2 ("BB-B"). According to the stratified randomisation, comparison was made between CC-C and CC-B arms ("Prior two-dose CoronaVac"), and separately between BB-C and BB-B arms ("Prior two-dose BNT162b2"). Data on sera collected on day 28 post-vaccination from all participants with paired day 0 and 28 sera (CC-C: n=100; CC-B: n=115; BB-C: n=111; BB-B: n=109) were analysed. Differences with p-values ≤ 0.05 were highlighted in bold.

	**Day 28**			
	**Third-dose CoronaVac**	**Third-dose BNT162b2**	**Fold-difference**	***p***
	**Mean**	**Mean**		
**Prior two-dose CoronaVac**				
$PRNT_{50}$ titer	9	102	12.4	**<0.0001**
$PRNT_{80}$ titer	6	53	8.4	**<0.0001**
$PRNT_{90}$ titer	6	35	6.0	**<0.0001**
**Prior two-dose BNT162b2**				
$PRNT_{50}$ titer	11	87	8.2	**<0.0001**
$PRNT_{80}$ titer	7	46	6.3	**<0.0001**
$PRNT_{90}$ titer	7	31	4.7	**<0.0001**

Table S7. Sensitivity analysis for the association between neutralizing antibodies 28 days post-vaccination and randomised third-dose CoronaVac or BNT162b2 vaccination. Linear regression on log_2 $PRNT_{50}$ titer with adjustment of covariates that were imbalanced at baseline (i.e., age and hypertension) were performed in post-hoc sensitivity analysis. The four study arms included participants who previously received two-dose CoronaVac and were randomised to receive a third dose of CoronaVac ("CC-C") or BNT162b2 ("CC-B"), and participants who previously received two-dose BNT162b2 and were randomised to receive a third dose of CoronaVac ("BB-C") or BNT162b2 ("BB-B"). According to the stratified randomisation, comparison was made between CC-C and CC-B arms ("Prior two-dose CoronaVac"), and separately between BB-C and BB-B arms ("Prior two-dose BNT162b2"), with third-dose CoronaVac as the reference group in both comparisons. Data on $PRNT_{50}$ against ancestral virus, Omicron BA.1 and BA.2 variants from a random subset of 20 participants selected from each study arm, and data on $PRNT_{50}$ against BA.2 variant from all participants with paired day 0 and 28 sera (CC-C: n=100; CC-B: n=115; BB-C: n=111; BB-B: n=109), on sera collected on day 28 post-vaccination were analysed. GMTR >1 indicate higher antibody levels in third-dose BNT162b2 recipients compared to third-dose CoronaVac recipients. Differences with p-values ≤ 0.05 were highlighted in bold. GMTR: geometric mean titer ratio.

		**Crude**			**Adjusted for age only**			**Adjusted for hypertension only**			**Adjusted for both age and hypertension**	
		**GMTR**	***p***		**GMTR**	***p***		**GMTR**	***p***		**GMTR**	***p***
**Prior two-dose CoronaVac**												
**Random subset of 80 participants**												
Ancestral virus		8.28	**<0.0001**		8.41	**<0.0001**		8.12	**<0.0001**		8.19	**<0.0001**
Omicron BA.1 variant		8.00	**<0.0001**		8.43	**<0.0001**		8.07	**<0.0001**		8.59	**<0.0001**
Omicron BA.2 variant		13.00	**<0.0001**		13.42	**<0.0001**		13.21	**<0.0001**		13.79	**<0.0001**
**All participants**												
Omicron BA.2 variant		11.86	**<0.0001**		11.62	**<0.0001**		11.86	**<0.0001**		11.59	**<0.0001**
**Prior two-dose BNT162b2**												
**Random subset of 80 participants**												
Ancestral virus		8.88	**<0.0001**		9.09	**<0.0001**		7.25	**<0.0001**		7.13	**<0.0001**
Omicron BA.1 variant		10.56	**<0.0001**		10.68	**<0.0001**		9.46	**<0.0001**		9.35	**<0.0001**
Omicron BA.2 variant		10.93	**<0.0001**		10.76	**<0.0001**		10.6	**<0.0001**		10.19	**<0.0001**
**All participants**												
Omicron BA.2 variant		8.16	**<0.0001**		8.08	**<0.0001**		8.08	**<0.0001**		7.91	**<0.0001**

Table S8. Proportions of participants who were responders of cell-mediated response at baseline (Day 0), Day 7 and Day 28 after randomised third-dose CoronaVac or BNT162b2 vaccination. The four study arms included participants who previously received two-dose CoronaVac and were randomised to receive a third dose of CoronaVac ("CC-C") or BNT162b2 ("CC-B"), and participants who previously received two-dose BNT162b2 and were randomised to receive a third dose of CoronaVac ("BB-C") or BNT162b2 ("BB-B"). Data on cell-mediated response (CMI) on PBMCs collected at baseline, day 7 and day 28 from a random subset of 20 participants selected from each study arm from participants in the CMI group was included. According to the stratified randomisation, comparison was made between CC-C and CC-B arms ("Prior two-dose CoronaVac"), and separately between BB-C and BB-B arms ("Prior two-dose BNT162b2"). Comparison was also made between paired baseline (day 0) and day 7 samples, or between paired baseline and day 28 samples, collected from each participant. Differences with p-values ≤ 0.05 were highlighted in bold.

	**Baseline**			**Day 7**			**Day 28**			**Baseline vs. Day 7**		**Baseline vs. Day 28**	
	**Third-dose CoronaVac**	**Third-dose BNT162b2**	***p***	**Third-dose CoronaVac**	**Third-dose BNT162b2**	***p***	**Third-dose CoronaVac**	**Third-dose BNT162b2**	***p***	**Third-dose CoronaVac**	**Third-dose BNT162b2**	**Third-dose CoronaVac**	**Third-dose BNT162b2**
	**n (%)**	**n (%)**		**n (%)**	**n (%)**		**n (%)**	**n (%)**		***p***	***p***	***p***	***p***
**Prior two-dose CoronaVac**													
IFNg-producing $CD4^+$ cells	14 (70)	12 (60)	0.7403	20 (100)	19 (95)	>0.9999	16 (80)	16 (80)	>0.9999	**0.0268**	**0.0231**	0.715	0.3006
IFNg-producing $CD8^+$ cells	10 (50)	6 (30)	0.3329	16 (80)	16 (80)	>0.9999	12 (60)	9 (45)	0.5266	0.0974	**0.0042**	0.7506	0.5136
IL4-producing $CD4^+$ cells	12 (60)	13 (65)	>0.9999	11 (55)	14 (70)	0.5136	12 (60)	9 (45)	0.5266	>0.9999	>0.9999	>0.9999	0.3404
**Prior two-dose BNT162b2**													
IFNg-producing $CD4^+$ cells	18 (90)	16 (80)	0.6579	17 (85)	15 (75)	0.6926	17 (85)	15 (75)	0.6926	>0.9999	>0.9999	>0.9999	>0.9999
IFNg-producing $CD8^+$ cells	10 (50)	8 (40)	0.7506	8 (40)	9 (45)	>0.9999	14 (70)	10 (50)	0.3329	0.7506	>0.9999	0.3329	0.7506
IL4-producing $CD4^+$ cells	11 (55)	12 (60)	>0.9999	11 (55)	15 (75)	0.3200	11 (55)	11 (55)	>0.9999	>0.9999	0.4996	>0.9999	>0.9999

Table S9. (A) Participant characteristics and (B) antibody and cell-mediated responses of all vaccinated participants at baseline, stratified by prior two-dose CoronaVac or BNT162b2. We enrolled 219 adults who previously received two doses of CoronaVac and 232 adults who previously received two doses of BNT162b2, and randomly assigned participants to receive either vaccine as a third dose in each group. Comparison was made between participants who had received primary series of CoronaVac ("Prior two-dose CoronaVac", i.e. CC-C plus CC-B arms) and participants who had received primary series of BNT162b2 ("Prior two-dose BNT162b2", i.e. BB-C plus BB-B arms). Differences with p-values ≤ 0.05 were highlighted in bold. ELISA: enzyme-linked immunosorbent assay; sVNT: surrogate virus neutralisation test; PRNT: plaque reduction neutralisation test; ICS: intracellular cytokine staining; OD: optical density; % inhibition: percent inhibition; $PRNT_{50}$, $PRNT_{80}$ and $PRNT_{90}$: PRNT titers with endpoints at 50%, 80% and 90% inhibition respectively.

Table S9A. Participant characteristics at baseline.

		**Prior two-dose CoronaVac (CC)**	**Prior two-dose BNT162b2 (BB)**		***p***
		**(n = 219)**	**(n = 232)**		
		**n (%)**	**n (%)**		
**Female**		111 (51)	110 (47)		1.0000
**Male**		108 (49)	122 (53)		0.3914
**Age (mean, SD)**		53 (12)	53 (11)		0.9909
**Age group (years)**					
<18		0 (0)	0 (0)		
18 - 29		10 (5)	12 (5)		
30 - 39		21 (10)	17 (7)		
40 - 49		47 (21)	53 (23)		
50 - 59		81 (37)	92 (40)		
60 - 69		53 (24)	46 (20)		
70 - 79		7 (3)	12 (5)		
**Ethnicity**					0.7017
Chinese		217 (99)	227 (98)		
Caucasian		1 (0)	2 (1)		
Japanese		1 (0)	1 (0)		
Latin American		0 (0)	1 (0)		
Pakistani		0 (0)	1 (0)		
**Obesity**					0.4827
Underweight		12 (5)	11 (5)		
Normal		97 (44)	90 (39)		
Overweight		42 (19)	57 (25)		
Obese		68 (31)	74 (32)		
**Chronic medical conditions**					
Any		42 (19)	62 (27)		0.0619
Lung disease, including COPD and asthma		2 (1)	1 (0)		1.0000
Heart disease		3 (1)	2 (1)		1.0000
Hypertension		19 (9)	26 (11)		0.3713
Diabetes		8 (4)	5 (2)		0.5811
Hypercholesterolemia		10 (5)	18 (8)		0.1849
Kidney disease		0 (0)	1 (0)		1.0000
Liver disease		3 (1)	4 (2)		1.0000
Cancer		5 (2)	1 (0)		0.2188
**Days between first and second dose of COVID-19 vaccination (mean, SD)**		**29 (2)**	**22 (2)**		**<0.0001**
**Days between second and third (study) dose of COVID-19 vaccination (mean, SD)**		**231 (26)**	**224 (31)**		**0.0215**
**Smoking**					
Ever		**15 (7)**	**5 (2)**		**0.0414**
Current		**9 (4)**	**2 (1)**		**0.0654**

Table S9B. Antibody and cell-mediated response at baseline.

	Received primary series of CoronaVac (CC)	Received primary series of BNT162b2 (BB)	Fold-difference	*p*
	(n = 219)	(n = 232)		
	Mean	Mean		
Antibody response				
**ELISA OD**				
Ancestral virus	**0.38**	**1.33**	**3.5**	**<0.0001**
**sVNT % inhibition**				
Ancestral virus	**16**	**68**	**4.2**	**<0.0001**
Omicron BA.1 variant	**6**	**8**	**1.3**	**0.0005**
Omicron BA.2 variant	**6**	**29**	**4.9**	**<0.0001**
**PRNT50 titer**				
Ancestral virus	**9**	**38**	**4.3**	**0.0001**
Omicron BA.1 variant	5	6	1.1	0.1404
Omicron BA.2 variant	5	5	1.0	0.5927
**PRNT80 titer**				
Ancestral virus	**6**	**19**	**3.4**	**0.0001**
Omicron BA.1 variant	5	5	1.0	0.9999
Omicron BA.2 variant	5	5	1.0	0.6564
**PRNT90 titer**				
Ancestral virus	**5**	**11**	**2.1**	**0.0001**
Omicron BA.1 variant	5	5	1.0	0.9999
Omicron BA.2 variant	5	5	1.0	0.3235
Cell-mediated response				
IFNg-producing $CD4^+$ responder (n, %)	26 (65)	34 (85)	-	0.0707
IFNg-producing $CD8^+$ responder (n, %)	16 (40)	18 (45)	-	0.8211
IL4-producing $CD4^+$ cells responder (n, %)	25 (62)	23 (57)	-	0.8195
IFNg-producing $CD4^+$ cells (per 100,000)	**750**	**2581**	**3.4**	**0.0019**
IFNg-producing $CD8^+$ cells (per 100,000)	128	176	1.4	0.6058
IL4-producing $CD4^+$ cells (per 100,000)	108	86	0.8	0.7160

Table S10. List of trial participants with SARS-CoV-2 infection and their method of identification. Altogether, (A) a total of 58 COVID-19 infection were identified either by RAT or PCR after third-dose vaccination until 31 May 2022 by various means, including (B) self-reported positive RAT or PCR result before the start of active surveillance, (C) during active surveillance, systematic monitoring for SARS-CoV-2 infection with self-administered RAT every 4 days, and (D) during active surveillance, upon a reported illness episode self-administered RAT daily starting from 4 days after the initiation of symptom diary. All COVID-19 RAT positive result identified during systematic monitoring and illness episodes were verified with the photo of the test kit whenever available. The four study arms included participants who previously received two-dose CoronaVac and were randomised to receive a third dose of CoronaVac ("CC-C") or BNT162b2 ("CC-B"), and participants who previously received two-dose BNT162b2 and were randomised to receive a third dose of CoronaVac ("BB-C") or BNT162b2 ("BB-B"). Data from 378/451 (84%) participants who agreed to participate in active surveillance for respiratory illness was included. RAT: rapid antigen test; PCR: polymerase chain reaction; Pos.: Positive; Sym.: Symptomatic. 1: Yes; 0: No; -: Unknown.

				A: Summary of SARS-CoV-2 infection identified		B: Reported before initiation of active surveillance			C: Reported during active surveillance - systematic monitoring for SARS-CoV-2 infection					D: Reported during active surveillance - Daily symptom diary upon an illness episode				
	Study arm	Date of third-dose vaccination (Month/ Year)	Date of COVID-19 infection (Month/ Year)	Pos. verified with picture of RAT	Sym. illness	Pos. by RAT or PCR	Pos. by RAT	Pos. by PCR	Pos. by RAT or PCR	Pos. by RAT	RAT result verified	Pos. by PCR	Sym. illness	Pos. by RAT or PCR	Pos. by RAT	RAT result verified	Pos. by PCR	Sym. illness
1	CC-C	11/21	2/22	0	0	1	1	1	0	0	-	-	-	-	-	-	-	-
2	CC-C	11/21	3/22	1	1	1	1	1	1	1	1	-	1	1	0	-	1	1
3	CC-B	11/21	3/22	1	1	0	0	0	0	0	1	0	1	1	1	1	-	1
4	CC-B	11/21	3/22	1	1	-	-	-	0	0	1	-	1	1	1	1	-	1
5	CC-B	12/21	3/22	1	1	1	1	-	1	1	1	-	1	1	1	0	1	1
6	CC-B	12/21	3/22	1	1	1	1	-	1	1	0	-	1	1	1	1	-	1
7	CC-B	12/21	3/22	1	0	1	1	0	1	0	1	1	0	1	0	-	1	0
8	BB-C	12/21	3/22	1	0	-	-	-	1	1	1	-	0	0	0	-	0	0
9	CC-B	12/21	3/22	0	0	1	1	1	0	0	-	0	-	-	-	-	-	-
10	CC-B	12/21	2/22	0	0	1	1	1	0	0	-	-	-	-	-	-	-	-
11	CC-B	12/21	3/22	1	1	-	0	-	1	1	1	0	1	1	1	1	1	1
12	CC-C	12/21	2/22	0	0	1	1	1	0	0	-	-	-	-	-	-	-	-
13	CC-C	12/21	3/22	0	1	-	0	-	1	1	0	-	1	1	1	0	-	1
14	BB-C	12/21	2/22	0	0	1	1	1	0	0	-	-	-	-	-	-	-	-
15	CC-C	12/21	2/22	1	1	1	1	1	0	0	1	0	0	0	0	-	0	1
16	CC-C	12/21	2/22	0	0	1	1	-	0	0	-	-	-	-	-	-	-	-
17	CC-C	12/21	3/22	0	0	1	1	1	0	0	-	-	-	-	-	-	-	-
18	CC-C	12/21	2/22	0	0	1	1	1	0	0	-	-	-	-	-	-	-	-

19	CC-C	12/21	2/22	0	0	1	1	1	0	0	-	-	-	-	-	-	-	-
20	CC-B	12/21	2/22	0	0	1	1	0	0	0	-	0	-	-	-	-	-	-
21	CC-B	12/21	3/22	1	1	1	1	1	0	0	1	0	1	1	-	-	1	1
22	CC-B	12/21	4/22	1	1	-	0	-	1	1	1	-	0	1	1	1	-	1
23	CC-B	12/21	3/22	1	1	1	1	1	1	1	1	-	1	1	1	1	-	1
24	BB-C	12/21	3/22	0	0	1	1	-	0	0	-	-	-	-	-	-	-	-
25	BB-C	12/21	3/22	1	1	0	0	0	1	1	1	-	0	1	1	1	-	1
26	CC-C	12/21	3/22	1	1	0	0	0	0	0	1	0	1	1	-	1	-	1
27	CC-B	12/21	3/22	1	1	-	0	-	1	1	1	-	1	0	0	-	-	1
28	CC-C	1/22	3/22	0	0	1	1	-	0	-	-	-	-	-	-	-	-	-
29	BB-C	1/22	4/22	1	1	0	0	0	1	1	1	1	1	1	1	1	-	1
30	BB-B	1/22	3/22	0	0	1	1	-	0	0	-	0	-	-	-	-	-	-
31	BB-B	1/22	3/22	0	0	1	1	0	0	0	-	0	-	-	-	-	-	-
32	BB-B	1/22	3/22	1	1	0	0	0	1	1	1	1	1	1	1	1	-	1
33	BB-B	1/22	3/22	1	1	0	0	0	0	0	1	-	1	1	1	1	-	1
34	BB-B	1/22	3/22	0	1	1	1	1	1	1	0	1	1	1	0	-	1	1
35	BB-B	1/22	3/22	0	1	1	1	1	1	1	0	1	1	1	0	-	1	1
36	CC-B	1/22	3/22	0	0	1	1	0	0	0	-	-	-	-	-	-	-	-
37	BB-C	1/22	2/22	0	0	1	0	1	0	0	-	-	-	-	-	-	-	-
38	BB-C	1/22	3/22	1	1	1	1	0	1	1	1	-	1	0	0	-	-	1
39	CC-C	1/22	3/22	0	0	1	1	0	0	0	-	-	-	-	-	-	-	-
40	CC-C	1/22	3/22	1	1	0	0	0	1	1	1	-	1	0	0	-	-	1
41	BB-C	1/22	2/22	0	0	1	0	1	0	0	-	0	-	-	-	-	-	-
42	CC-B	1/22	3/22	1	1	1	-	1	1	1	1	-	0	0	0	-	0	1
43	BB-B	1/22	3/22	1	1	1	1	-	1	1	1	1	1	-	-	-	-	1
44	BB-B	1/22	3/22	0	0	1	1	1	0	0	-	0	-	-	-	-	-	-
45	BB-B	1/22	3/22	1	1	1	1	0	1	1	1	-	1	0	0	-	-	1
46	BB-C	1/22	4/22	1	1	0	0	0	1	1	1	1	1	0	0	-	0	1
47	BB-C	1/22	2/22	0	0	1	1	-	0	0	-	-	-	-	-	-	-	-
48	BB-C	1/22	3/22	1	1	1	1	-	0	0	1	0	1	0	0	-	0	1
49	BB-B	1/22	3/22	1	1	1	1	0	1	1	1	0	1	1	0	-	1	1
50	BB-B	1/22	2/22	0	0	1	1	-	0	0	-	-	-	-	-	-	-	-
51	BB-B	1/22	5/22	1	1	-	-	-	1	1	1	1	1	1	0	-	1	1
52	BB-C	1/22	2/22	0	0	1	1	1	0	0	-	-	-	-	-	-	-	-
53	BB-C	1/22	2/22	0	0	1	1	1	0	0	-	0	-	-	-	-	-	-
54	BB-C	1/22	3/22	1	1	1	1	1	0	0	1	-	0	-	-	-	-	1
55	BB-C	1/22	3/22	1	1	-	0	-	0	0	1	-	0	1	0	-	1	1
56	BB-C	1/22	2/22	0	0	1	1	1	0	0	-	0	-	-	-	-	-	-
57	CC-B	1/22	3/22	0	0	1	1	1	0	0	-	0	-	-	-	-	-	-
58	BB-B	1/22	3/22	1	1	1	1	0	1	1	1	-	1	1	0	-	1	1

Supplementary Figures

Figure S1. A detailed flow chart of participant enrolment for this open-label randomised trial of third-dose CoronaVac or BNT162b2 (Cobovax study), including reasons for exclusion at each stage. (A) From eligibility assessment for enrolment to randomisation of study intervention. **(B)** From randomisation to administration of study intervention. **(C)** From administration of study intervention to selection of paired Day 0 and Day 28 blood samples for laboratory testing. We enrolled 219 adults who previously received two doses of CoronaVac (CC) and 232 adults who previously received two doses of BNT162b2 (BB), who were randomly assigned to receive either vaccine as a third dose in each group. Therefore, the four study arms included participants who previously received two-dose CoronaVac and were randomised to receive a third dose of CoronaVac ("CC-C") or BNT162b2 ("CC-B"), and participants who previously received two-dose BNT162b2 and were randomised to receive a third dose of CoronaVac ("BB-C") or BNT162b2 ("BB-B").

(Continued to next page)

A

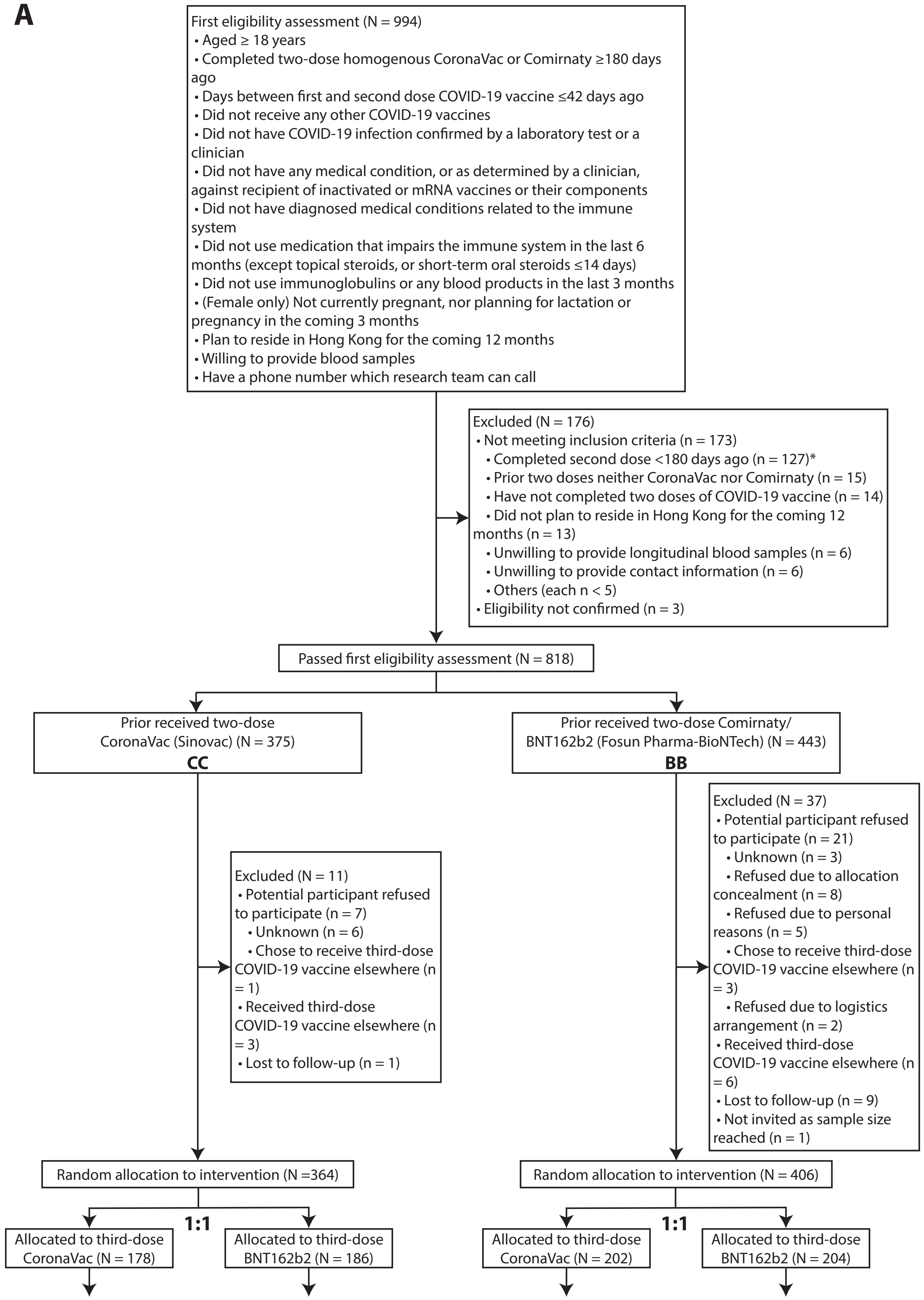
First eligibility assessment (N = 994)
• Aged ≥ 18 years
• Completed two-dose homogenous CoronaVac or Comirnaty ≥180 days ago
• Days between first and second dose COVID-19 vaccine ≤42 days ago
• Did not receive any other COVID-19 vaccines
• Did not have COVID-19 infection confirmed by a laboratory test or a clinician
• Did not have any medical condition, or as determined by a clinician, against recipient of inactivated or mRNA vaccines or their components
• Did not have diagnosed medical conditions related to the immune system
• Did not use medication that impairs the immune system in the last 6 months (except topical steroids, or short-term oral steroids ≤14 days)
• Did not use immunoglobulins or any blood products in the last 3 months
• (Female only) Not currently pregnant, nor planning for lactation or pregnancy in the coming 3 months
• Plan to reside in Hong Kong for the coming 12 months
• Willing to provide blood samples
• Have a phone number which research team can call
Excluded (N = 176)
• Not meeting inclusion criteria (n = 173)
• Completed second dose <180 days ago (n = 127)*
• Prior two doses neither CoronaVac nor Comirnaty (n = 15)
• Have not completed two doses of COVID-19 vaccine (n = 14)
• Did not plan to reside in Hong Kong for the coming 12 months (n = 13)
• Unwilling to provide longitudinal blood samples (n = 6)
• Unwilling to provide contact information (n = 6)
• Others (each n < 5)
• Eligibility not confirmed (n = 3)
Passed first eligibility assessment (N = 818)
Prior received two-dose CoronaVac (Sinovac) (N = 375)
CC
Prior received two-dose Comirnaty/ BNT162b2 (Fosun Pharma-BioNTech) (N = 443)
BB
Excluded (N = 11)
• Potential participant refused to participate (n = 7)
• Unknown (n = 6)
• Chose to receive third-dose COVID-19 vaccine elsewhere (n = 1)
• Received third-dose COVID-19 vaccine elsewhere (n = 3)
• Lost to follow-up (n = 1)
Excluded (N = 37)
• Potential participant refused to participate (n = 21)
• Unknown (n = 3)
• Refused due to allocation concealment (n = 8)
• Refused due to personal reasons (n = 5)
• Chose to receive third-dose COVID-19 vaccine elsewhere (n = 3)
• Refused due to logistics arrangement (n = 2)
• Received third-dose COVID-19 vaccine elsewhere (n = 6)
• Lost to follow-up (n = 9)
• Not invited as sample size reached (n = 1)
Random allocation to intervention (N =364)
Random allocation to intervention (N = 406)
1:1
1:1
Allocated to third-dose CoronaVac (N = 178)
Allocated to third-dose BNT162b2 (N = 186)
Allocated to third-dose CoronaVac (N = 202)
Allocated to third-dose BNT162b2 (N = 204)

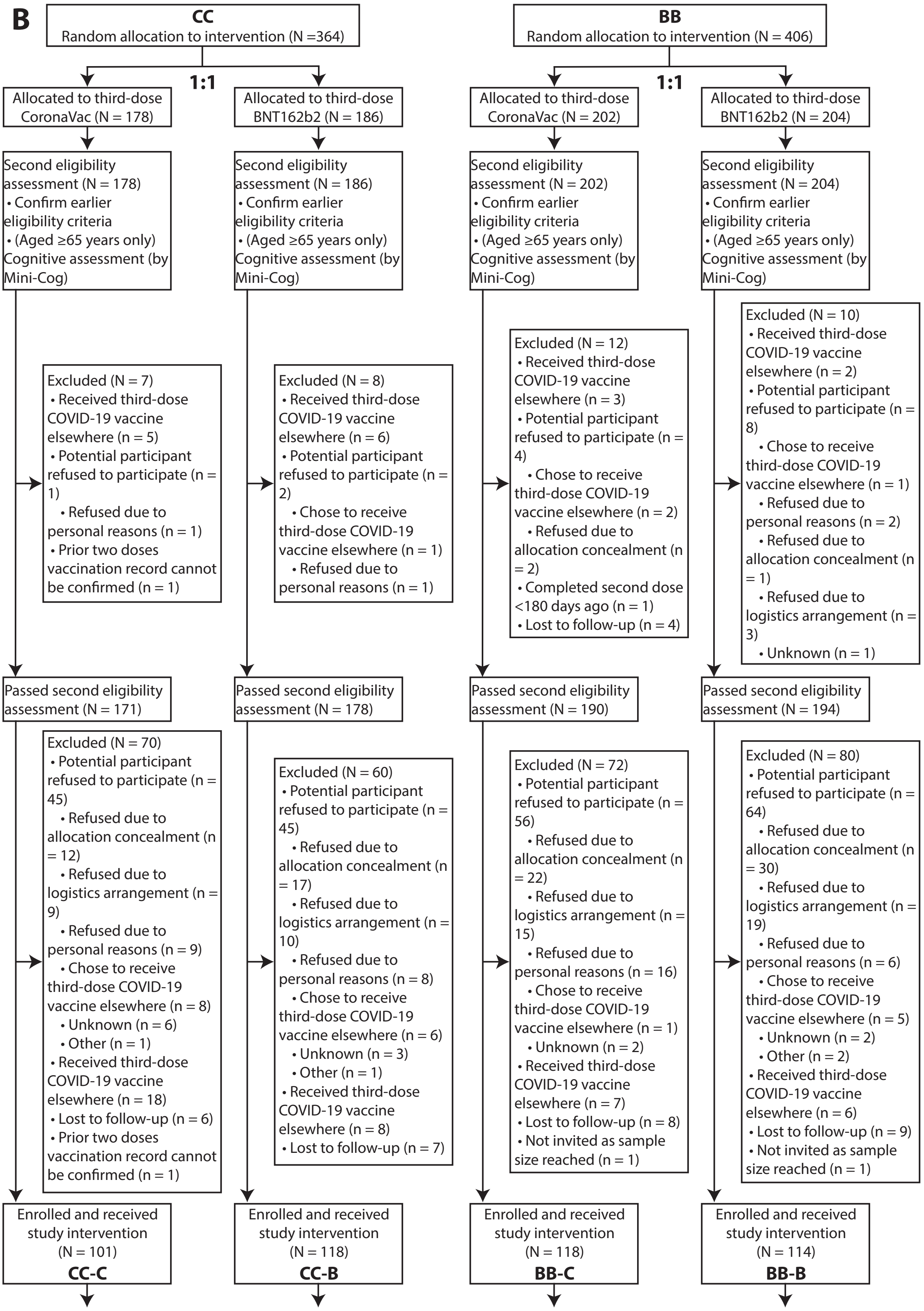
B
CC
Random allocation to intervention (N =364)
BB
Random allocation to intervention (N = 406)
1:1
1:1
Allocated to third-dose CoronaVac (N = 178)
Allocated to third-dose BNT162b2 (N = 186)
Allocated to third-dose CoronaVac (N = 202)
Allocated to third-dose BNT162b2 (N = 204)
Second eligibility assessment (N = 178) • Confirm earlier eligibility criteria • (Aged ≥65 years only) Cognitive assessment (by Mini-Cog)
Second eligibility assessment (N = 186) • Confirm earlier eligibility criteria • (Aged ≥65 years only) Cognitive assessment (by Mini-Cog)
Second eligibility assessment (N = 202) • Confirm earlier eligibility criteria • (Aged ≥65 years only) Cognitive assessment (by Mini-Cog)
Second eligibility assessment (N = 204) • Confirm earlier eligibility criteria • (Aged ≥65 years only) Cognitive assessment (by Mini-Cog)
Excluded (N = 7) • Received third-dose COVID-19 vaccine elsewhere (n = 5) • Potential participant refused to participate (n = 1) • Refused due to personal reasons (n = 1) • Prior two doses vaccination record cannot be confirmed (n = 1)
Excluded (N = 8) • Received third-dose COVID-19 vaccine elsewhere (n = 6) • Potential participant refused to participate (n = 2) • Chose to receive third-dose COVID-19 vaccine elsewhere (n = 1) • Refused due to personal reasons (n = 1)
Excluded (N = 12) • Received third-dose COVID-19 vaccine elsewhere (n = 3) • Potential participant refused to participate (n = 4) • Chose to receive third-dose COVID-19 vaccine elsewhere (n = 2) • Refused due to allocation concealment (n = 2) • Completed second dose <180 days ago (n = 1) • Lost to follow-up (n = 4)
Excluded (N = 10) • Received third-dose COVID-19 vaccine elsewhere (n = 2) • Potential participant refused to participate (n = 8) • Chose to receive third-dose COVID-19 vaccine elsewhere (n = 1) • Refused due to personal reasons (n = 2) • Refused due to allocation concealment (n = 1) • Refused due to logistics arrangement (n = 3) • Unknown (n = 1)
Passed second eligibility assessment (N = 171)
Passed second eligibility assessment (N = 178)
Passed second eligibility assessment (N = 190)
Passed second eligibility assessment (N = 194)
Excluded (N = 70) • Potential participant refused to participate (n = 45) • Refused due to allocation concealment (n = 12) • Refused due to logistics arrangement (n = 9) • Refused due to personal reasons (n = 9) • Chose to receive third-dose COVID-19 vaccine elsewhere (n = 8) • Unknown (n = 6) • Other (n = 1) • Received third-dose COVID-19 vaccine elsewhere (n = 18) • Lost to follow-up (n = 6) • Prior two doses vaccination record cannot be confirmed (n = 1)
Excluded (N = 60) • Potential participant refused to participate (n = 45) • Refused due to allocation concealment (n = 17) • Refused due to logistics arrangement (n = 10) • Refused due to personal reasons (n = 8) • Chose to receive third-dose COVID-19 vaccine elsewhere (n = 6) • Unknown (n = 3) • Other (n = 1) • Received third-dose COVID-19 vaccine elsewhere (n = 8) • Lost to follow-up (n = 7)
Excluded (N = 72) • Potential participant refused to participate (n = 56) • Refused due to allocation concealment (n = 22) • Refused due to logistics arrangement (n = 15) • Refused due to personal reasons (n = 16) • Chose to receive third-dose COVID-19 vaccine elsewhere (n = 1) • Unknown (n = 2) • Received third-dose COVID-19 vaccine elsewhere (n = 7) • Lost to follow-up (n = 8) • Not invited as sample size reached (n = 1)
Excluded (N = 80) • Potential participant refused to participate (n = 64) • Refused due to allocation concealment (n = 30) • Refused due to logistics arrangement (n = 19) • Refused due to personal reasons (n = 6) • Chose to receive third-dose COVID-19 vaccine elsewhere (n = 5) • Unknown (n = 2) • Other (n = 2) • Received third-dose COVID-19 vaccine elsewhere (n = 6) • Lost to follow-up (n = 9) • Not invited as sample size reached (n = 1)
Enrolled and received study intervention (N = 101) CC-C
Enrolled and received study intervention (N = 118) CC-B
Enrolled and received study intervention (N = 118) BB-C
Enrolled and received study intervention (N = 114) BB-B

C

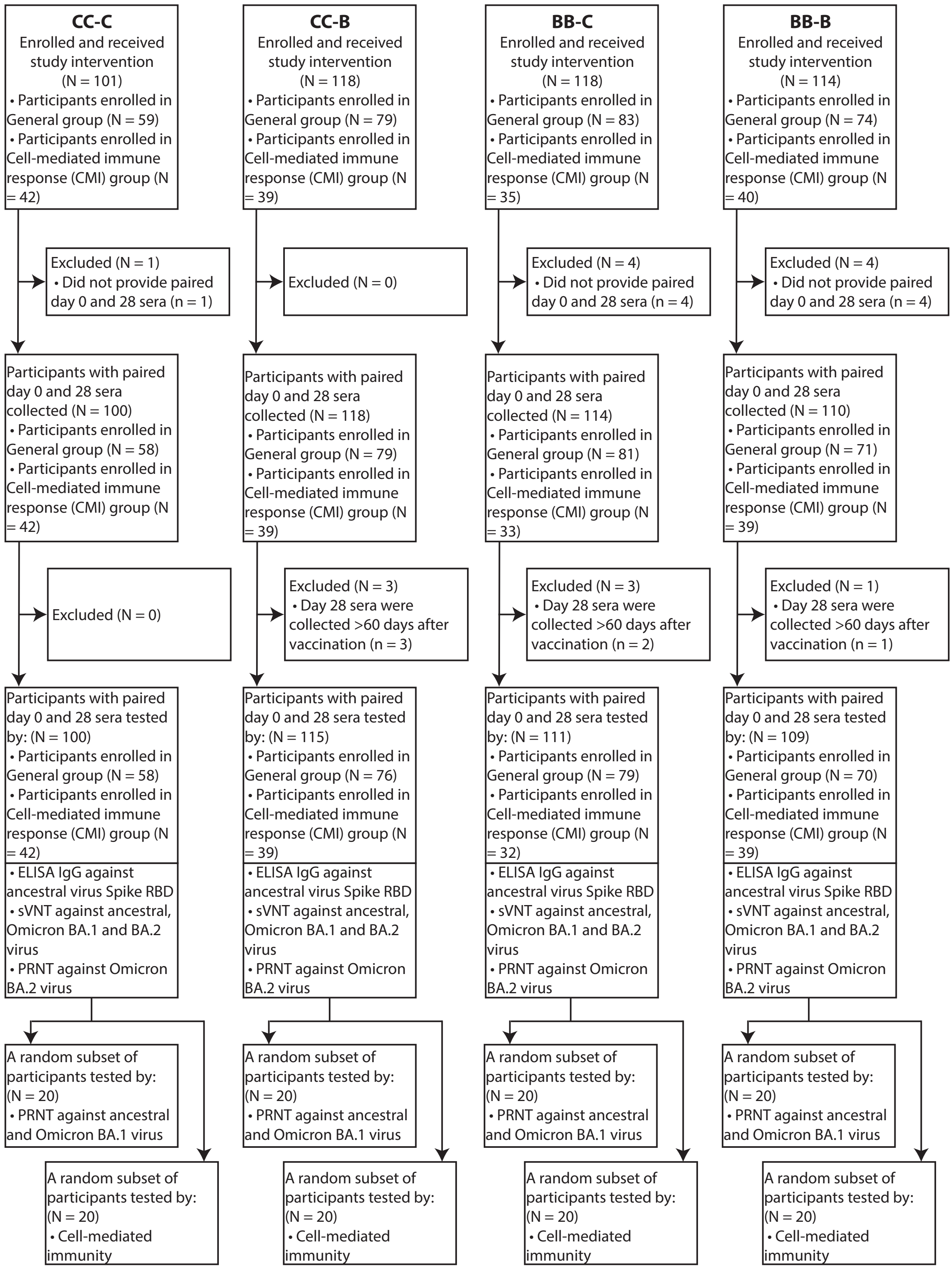
CC-C
Enrolled and received study intervention (N = 101)
• Participants enrolled in General group (N = 59)
• Participants enrolled in Cell-mediated immune response (CMI) group (N = 42)
Excluded (N = 1)
• Did not provide paired day 0 and 28 sera (n = 1)
Participants with paired day 0 and 28 sera collected (N = 100)
• Participants enrolled in General group (N = 58)
• Participants enrolled in Cell-mediated immune response (CMI) group (N = 42)
Excluded (N = 0)
Participants with paired day 0 and 28 sera tested by: (N = 100)
• Participants enrolled in General group (N = 58)
• Participants enrolled in Cell-mediated immune response (CMI) group (N = 42)
• ELISA IgG against ancestral virus Spike RBD
• sVNT against ancestral, Omicron BA.1 and BA.2 virus
• PRNT against Omicron BA.2 virus
A random subset of participants tested by: (N = 20)
• PRNT against ancestral and Omicron BA.1 virus
A random subset of participants tested by: (N = 20)
• Cell-mediated immunity
CC-B
Enrolled and received study intervention (N = 118)
• Participants enrolled in General group (N = 79)
• Participants enrolled in Cell-mediated immune response (CMI) group (N = 39)
Excluded (N = 0)
Participants with paired day 0 and 28 sera collected (N = 118)
• Participants enrolled in General group (N = 79)
• Participants enrolled in Cell-mediated immune response (CMI) group (N = 39)
Excluded (N = 3)
• Day 28 sera were collected >60 days after vaccination (n = 3)
Participants with paired day 0 and 28 sera tested by: (N = 115)
• Participants enrolled in General group (N = 76)
• Participants enrolled in Cell-mediated immune response (CMI) group (N = 39)
• ELISA IgG against ancestral virus Spike RBD
• sVNT against ancestral, Omicron BA.1 and BA.2 virus
• PRNT against Omicron BA.2 virus
A random subset of participants tested by: (N = 20)
• PRNT against ancestral and Omicron BA.1 virus
A random subset of participants tested by: (N = 20)
• Cell-mediated immunity
BB-C
Enrolled and received study intervention (N = 118)
• Participants enrolled in General group (N = 83)
• Participants enrolled in Cell-mediated immune response (CMI) group (N = 35)
Excluded (N = 4)
• Did not provide paired day 0 and 28 sera (n = 4)
Participants with paired day 0 and 28 sera collected (N = 114)
• Participants enrolled in General group (N = 81)
• Participants enrolled in Cell-mediated immune response (CMI) group (N = 33)
Excluded (N = 3)
• Day 28 sera were collected >60 days after vaccination (n = 2)
Participants with paired day 0 and 28 sera tested by: (N = 111)
• Participants enrolled in General group (N = 79)
• Participants enrolled in Cell-mediated immune response (CMI) group (N = 32)
• ELISA IgG against ancestral virus Spike RBD
• sVNT against ancestral, Omicron BA.1 and BA.2 virus
• PRNT against Omicron BA.2 virus
A random subset of participants tested by: (N = 20)
• PRNT against ancestral and Omicron BA.1 virus
A random subset of participants tested by: (N = 20)
• Cell-mediated immunity
BB-B
Enrolled and received study intervention (N = 114)
• Participants enrolled in General group (N = 74)
• Participants enrolled in Cell-mediated immune response (CMI) group (N = 40)
Excluded (N = 4)
• Did not provide paired day 0 and 28 sera (n = 4)
Participants with paired day 0 and 28 sera collected (N = 110)
• Participants enrolled in General group (N = 71)
• Participants enrolled in Cell-mediated immune response (CMI) group (N = 39)
Excluded (N = 1)
• Day 28 sera were collected >60 days after vaccination (n = 1)
Participants with paired day 0 and 28 sera tested by: (N = 109)
• Participants enrolled in General group (N = 70)
• Participants enrolled in Cell-mediated immune response (CMI) group (N = 39)
• ELISA IgG against ancestral virus Spike RBD
• sVNT against ancestral, Omicron BA.1 and BA.2 virus
• PRNT against Omicron BA.2 virus
A random subset of participants tested by: (N = 20)
• PRNT against ancestral and Omicron BA.1 virus
A random subset of participants tested by: (N = 20)
• Cell-mediated immunity

Figure S2. Solicited local and systemic reactions during the 7 days after randomised third-dose CoronaVac or BNT162b2 vaccination. For solicited systemic reactions only the most frequently reported reactions are shown. The four study arms included participants who previously received two-dose CoronaVac and were randomised to receive a third dose of CoronaVac ("CC-C") or BNT162b2 (left panel), and participants who previously received two-dose BNT162b2 and were randomised to receive a third dose of CoronaVac ("BB-C") or BNT162b2 ("BB-B") (right panel). Data from 424/451 (94%) participants who reported adverse reactions for at least 7 days post-vaccination was included. Red: Randomised and received a third dose of CoronaVac; Blue: Randomised and received a third dose of BNT162b2.

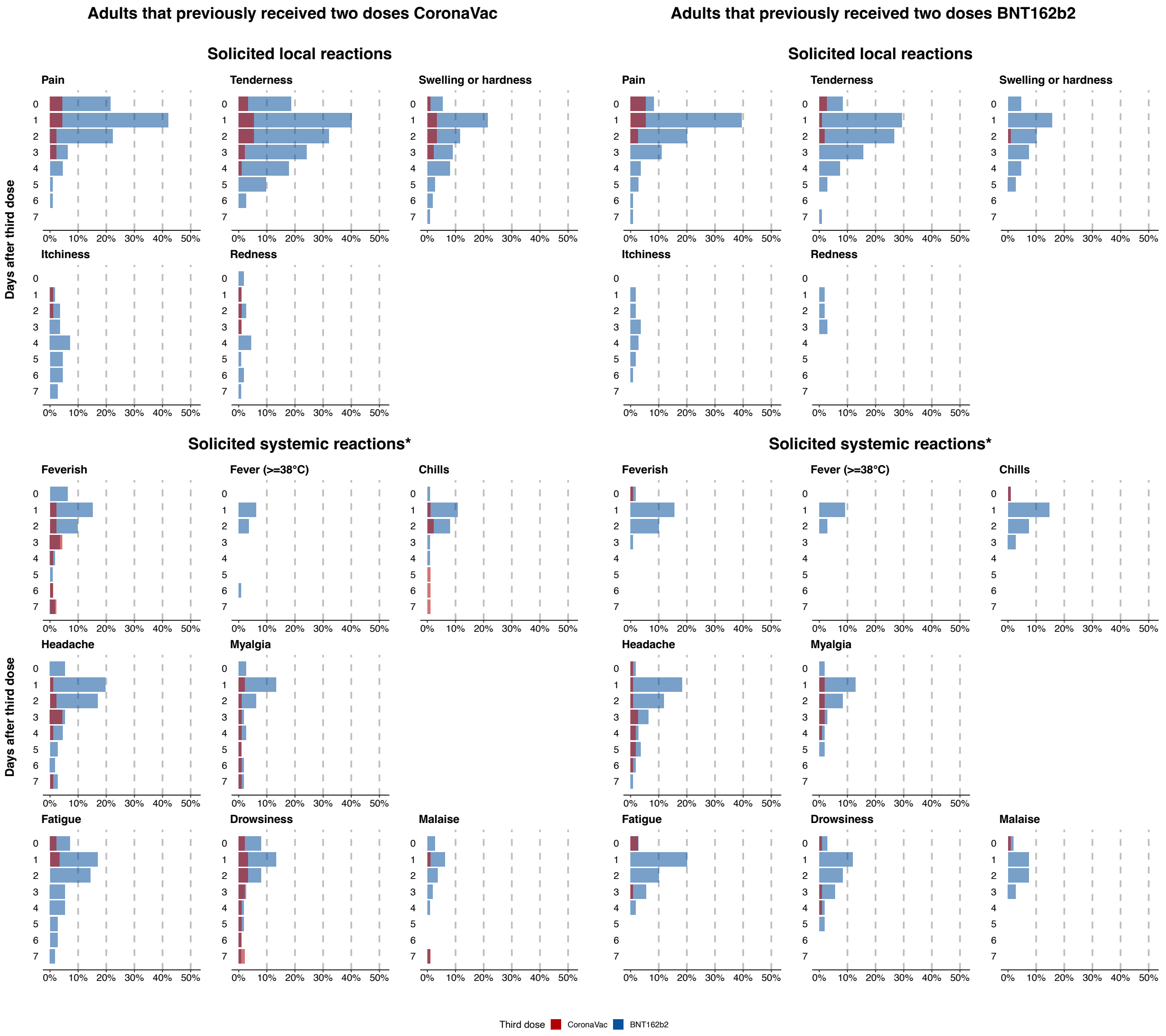

Figure S3. Serum neutralising antibodies measured by live virus plaque reduction neutralization test (PRNT) against (A) ancestral SARS-CoV-2 virus, (B) Omicron BA.1 and (C) Omicron BA.2 variants, or by (D-F) surrogate virus neutralization test (sVNT) respectively, at baseline and 28 days after randomised third-dose CoronaVac or BNT162b2 vaccination, in the random subset of 20 participants from each study arm. PRNT titers were evaluated with endpoint at 50% inhibition (PRNT50). The four study arms included participants who previously received two-dose CoronaVac and were randomised to receive a third dose of CoronaVac ("CC-C") or BNT162b2 ("CC-B"), and participants who previously received two-dose BNT162b2 and were randomised to receive a third dose of CoronaVac ("BB-C") or BNT162b2 ("BB-B"). Data from a random subset of 20 participants selected to test by PRNT against all three virus strains (ancestral virus, Omicron BA.1 subvariant, Omicron BA.2 subvariant) from each of the four study arms was included. The symbol X and the numbers above each panel indicate the mean level, and p-values for comparisons with significant p-values ≤ 0.05 was provided.

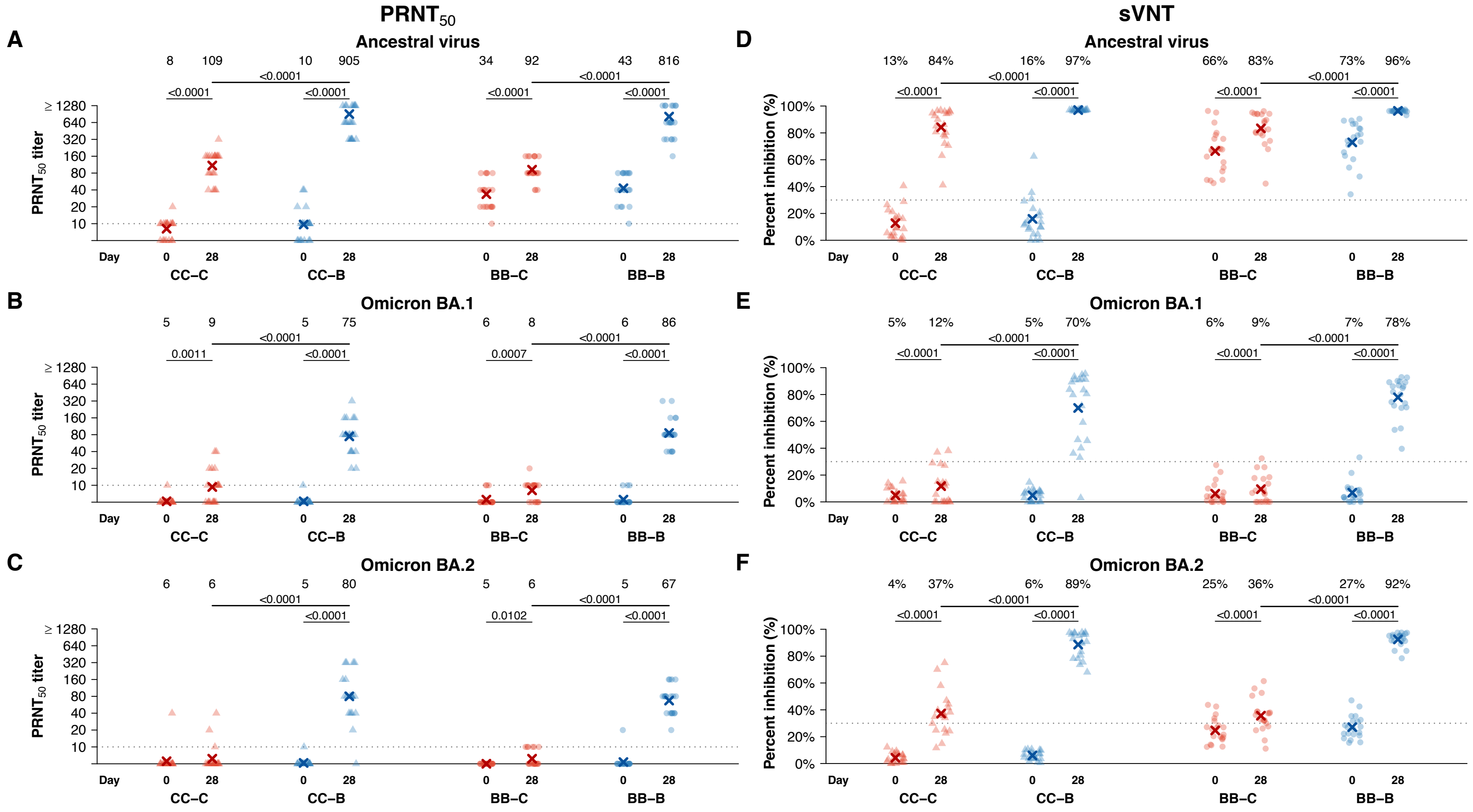

Figure S4. Fold-change of boosting of (A) neutralising antibody response and (B) cell-mediated response from baseline after randomised third-dose CoronaVac or BNT162b2 vaccination, in random subsets of 20 participants from each study arm. Neutralising antibody response was measured by live virus plaque reduction neturalization test (PRNT) against ancestral SARS-CoV-2 virus, Omicron BA.1 and BA.2 subvariants at baseline (Day 0) and Day 28, and cell-mediated response by intracellular cytokine staining (ICS) at baseline, Day 7 and Day 28 upon stimulation by overlapping peptide pool representing the SARS-CoV-2 structural proteins (spike, nucleocapsid, envelope and membrane). PRNT titers were evaluated with endpoint at 50% inhibition (PRNT50). The four study arms included participants who previously received two-dose CoronaVac and were randomised to receive a third dose of CoronaVac ("CC-C") or BNT162b2 ("CC-B"), and participants who previously received two-dose BNT162b2 and were randomised to receive a third dose of CoronaVac ("BB-C") or BNT162b2 ("BB-B"). For neutralising antibody response, data on sera collected from a random subset of 20 participants selected to test by PRNT against all three virus strains (ancestral virus, Omicron BA.1 subvariant, Omicron BA.2 subvariant) from each of the four study arms was included. For cell-mediated response, data on PBMCs collected from a separate random subset of 20 participants selected from each study arm from participants in the CMI group was included. The symbol X and the numbers above each panel indicate the fold-change compared to baseline (Day 0). D28/D0: comparison between Day 28 against Day 0; D7/D0: comparison between Day 7 against Day 0.

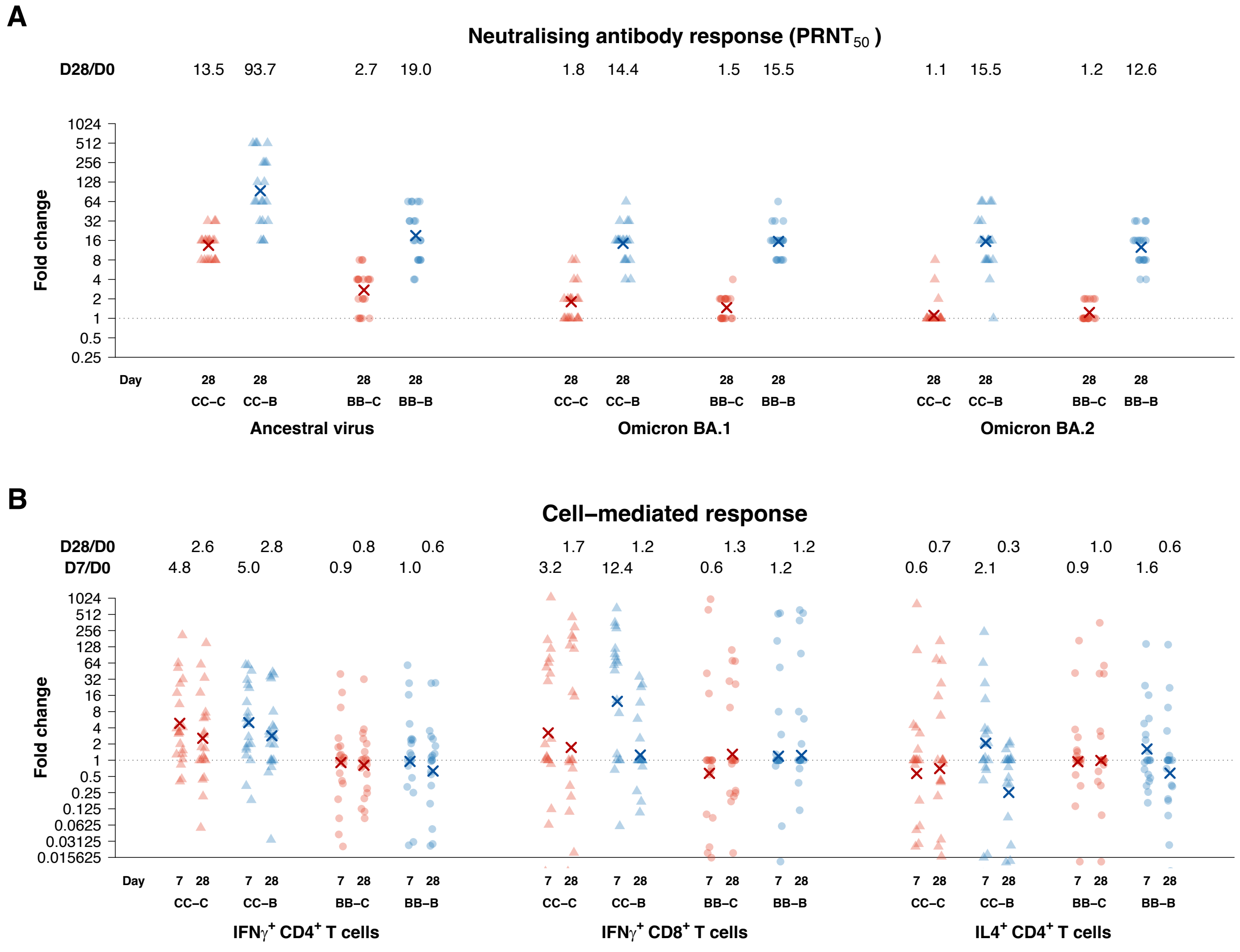

Figure S5. Correlation between surrogate virus neutralization test (sVNT) and live virus plaque reduction neutralization test (PRNT), for serum neutralizing antibodies against ancestral SARS-CoV-2 virus, Omicron BA.1 and BA.2 virus 28 days after randomised third-dose CoronaVac or BNT162b2 vaccination. PRNT titers were evaluated with endpoint at 50% inhibition (PRNT50). The four study arms included participants who previously received two-dose CoronaVac and were randomised to receive a third dose of CoronaVac ("CC-C") or BNT162b2 ("CC-B"), and participants who previously received two-dose BNT162b2 and were randomised to receive a third dose of CoronaVac ("BB-C") or BNT162b2 ("BB-B"). For ancestral virus and Omicron BA.1 subvariant, sVNT and PRNT data on sera collected on day 28 post-vaccination from a random subset of 20 participants selected from each study arm was included. For Omicron BA.2 subvariant, sVNT and PRNT data on sera collected on day 28 post-vaccination from all participants (with paired sera) was included. Red: Ancestral virus; Green: Omicron BA.1; Purple: Omicron BA.2.

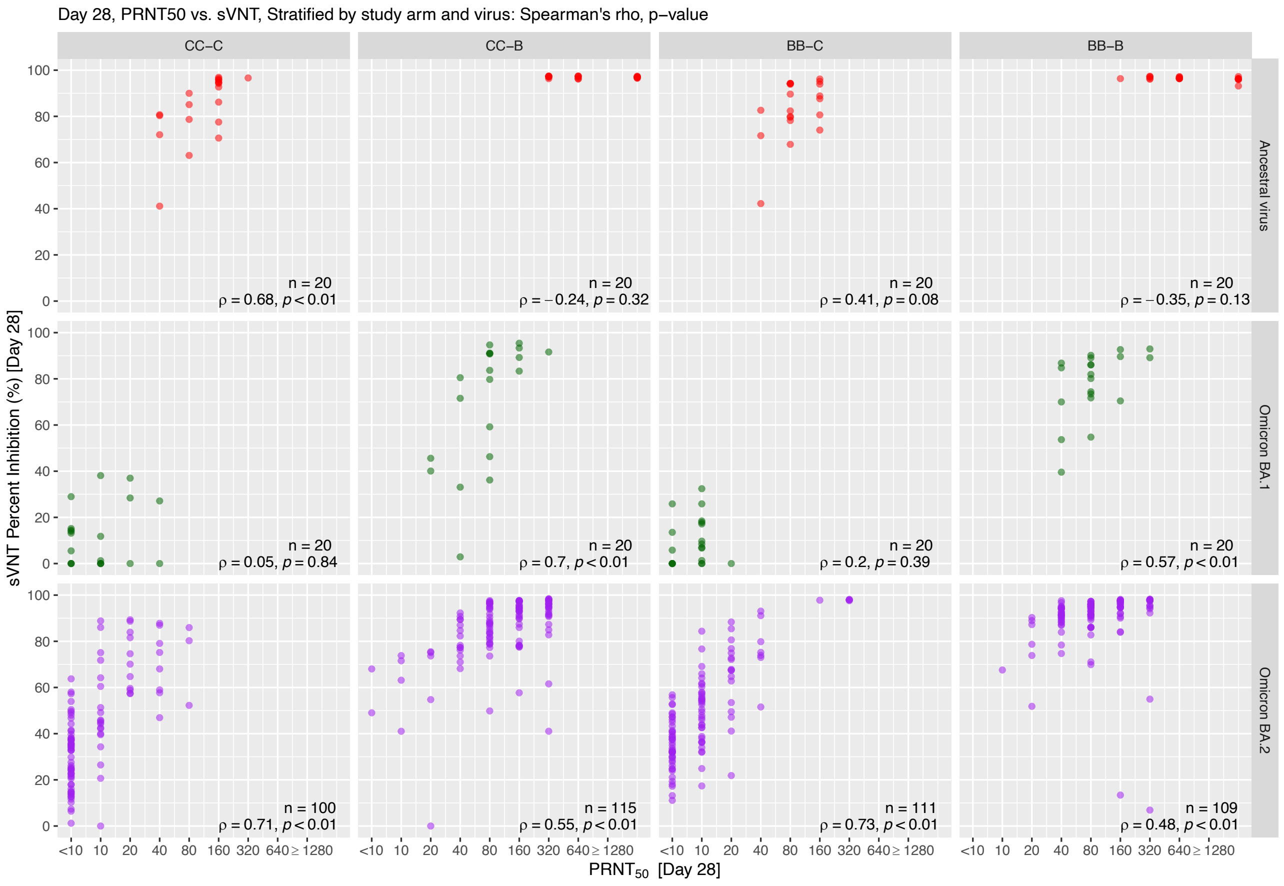

Figure S6. Serum antibodies against ancestral SARS-CoV-2 virus at baseline and Day 28 after randomised third-dose CoronaVac or BNT162b2 vaccination, measured by (A) ELISA, (B) surrogate virus neutralization test (sVNT) or (C) live virus plaque reduction neutralization test (PRNT). PRNT titers were evaluated with endpoint at 50% inhibition (PRNT50). The four study arms included participants who previously received two-dose CoronaVac and were randomised to receive a third dose of CoronaVac ("CC-C") or BNT162b2 ("CC-B"), and participants who previously received two-dose BNT162b2 and were randomised to receive a third dose of CoronaVac ("BB-C") or BNT162b2 ("BB-B"). For ELISA and sVNT, data on sera collected at baseline and on day 28 post-vaccination from all participants (with paired sera) was included. For PRNT, data on sera collected at baseline and on day 28 from a random subset of 20 participants selected from each study arm was included. The symbol X and the numbers above each panel indicate the mean level, and p-values for comparisons with significant p-values ≤ 0.05 was provided.

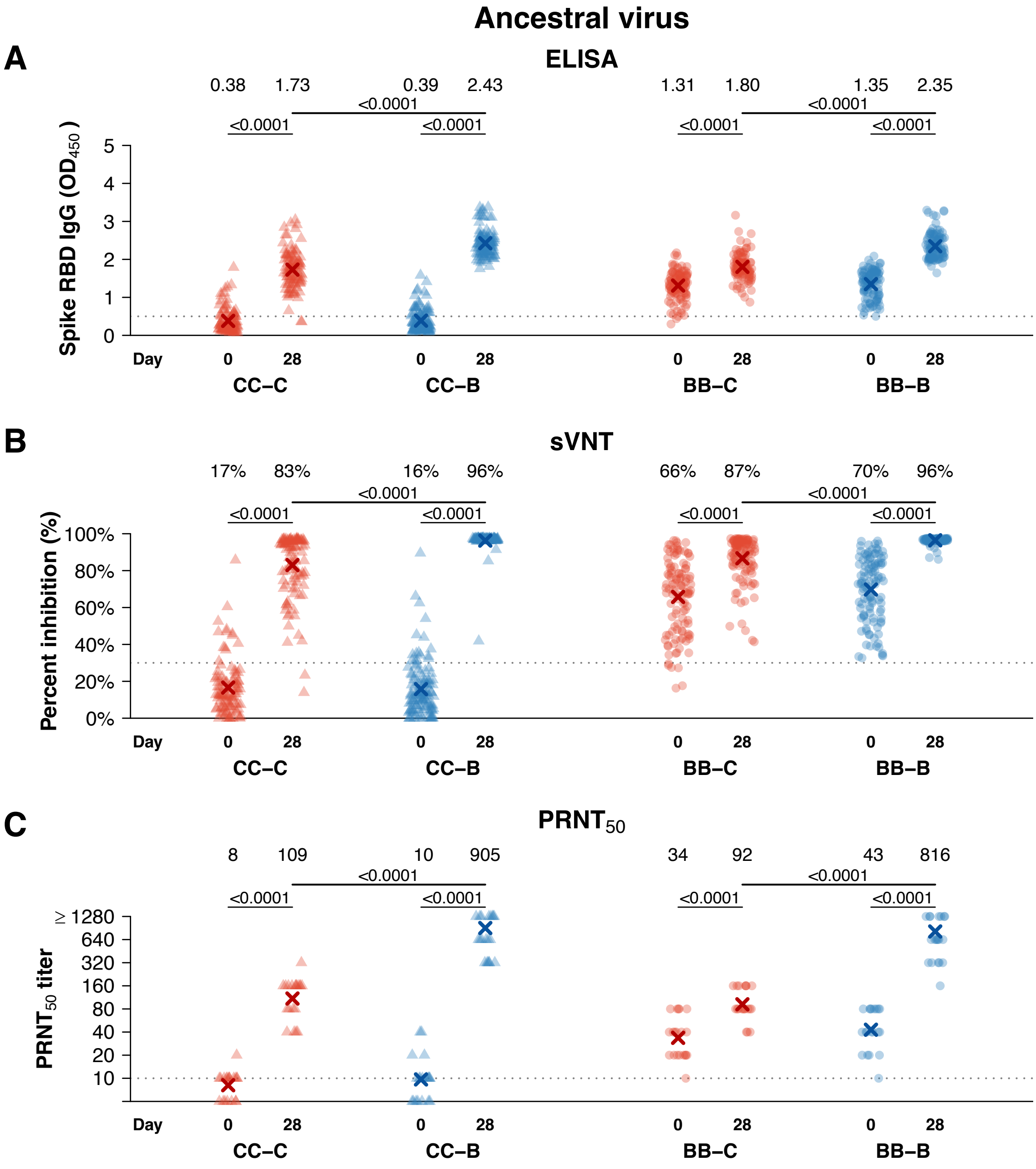

Figure S7. Serum neutralising antibodies against (A-C) ancestral SARS-CoV-2 virus, (D-F) Omicron BA.1 and (G-I) Omicron BA.2 variants at baseline (Day 0) and Day 28 after randomised third-dose CoronaVac or BNT162b2 vaccination, measured by live virus plaque reduction neutralization test (PRNT) with endpoints at 50% (PRNT50), 80% (PRNT80) and 90% (PRNT90) respectively. The four study arms included participants who previously received two-dose CoronaVac and were randomised to receive a third dose of CoronaVac ("CC-C") or BNT162b2 ("CC-B"), and participants who previously received two-dose BNT162b2 and were randomised to receive a third dose of CoronaVac ("BB-C") or BNT162b2 ("BB-B"). Data on PRNT against ancestral virus and Omicron BA.1 variant on sera collected at baseline and day 28 from a random subset of 20 participants selected from each study arm was included. Data on PRNT against Omicron BA.2 variant on sera collected on day 28 post-vaccination from all participants, and on sera collected at baseline was obtained from a random subset of 20 participants selected from each study arm, was included. The symbol X and the numbers above each panel indicate the mean level, and p-values for comparisons with significant p-values ≤ 0.05 was provided.

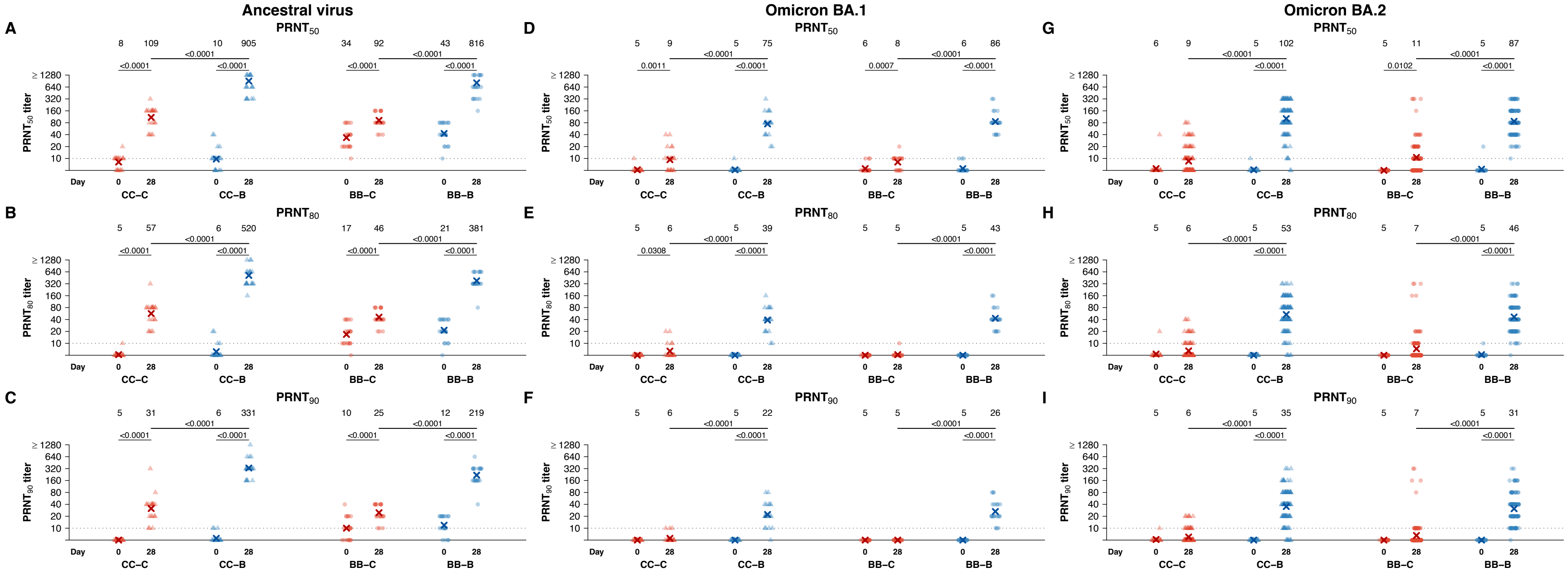

Figure S8. IL-4 producing CD4⁺ T cells at baseline, Day 7 and Day 28 after randomised third-dose CoronaVac or BNT162b2 vaccination. IL-4⁺ CD4⁺ T cell response was evaluated upon stimulation by overlapping peptide pool representing the SARS-CoV-2 structural proteins (spike, nucleocapsid, envelope and membrane). The four study arms included participants who previously received two-dose CoronaVac and were randomised to receive a third dose of CoronaVac ("CC-C") or BNT162b2 ("CC-B"), and participants who previously received two-dose BNT162b2 and were randomised to receive a third dose of CoronaVac ("BB-C") or BNT162b2 ("BB-B"). Data on cell-mediated response (CMI) on PBMCs collected at baseline, day 7 and day 28 from a random subset of 20 participants selected from each study arm from participants in the CMI group was included. The dotted lines represent the lower limits of detection based on DMSO controls and participants with values above these thresholds are considered as responders: 0.000039% for CD4⁺ IL4⁺ T cells. The numbers above each panel indicate the mean level, the percentages indicate the proportion of responder, and p-values for comparisons with significant p-values ≤ 0.05 was provided.

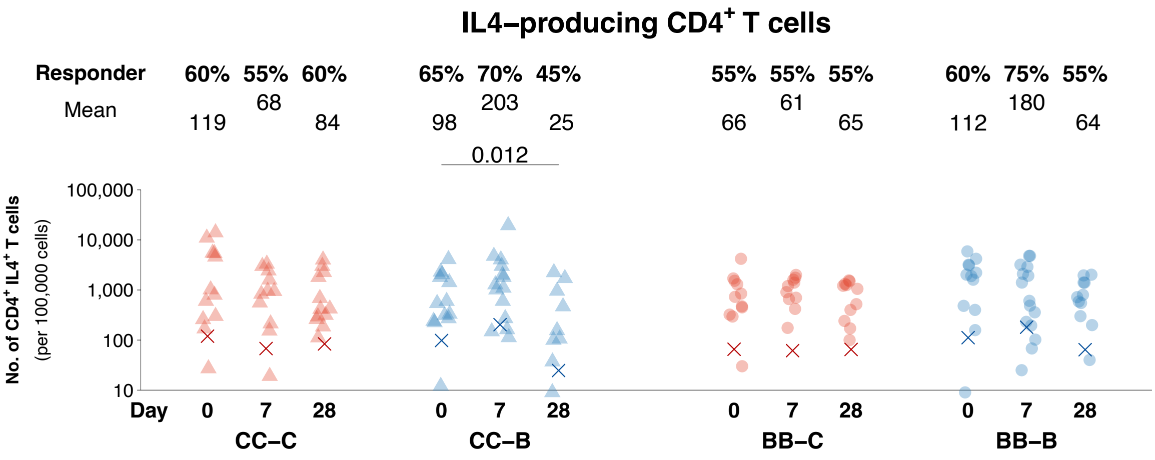

Figure S9. FACS gating strategy for intra cellular cytokine staining (ICS). Plots attained from flow cytometry of PBMCs for IFNγ staining following stimulation by overlapping peptide pool representing the SARS-CoV-2 structural proteins (spike, nucleocapsid, envelope and membrane). FACS: Fluorescence-activated cell sorting.

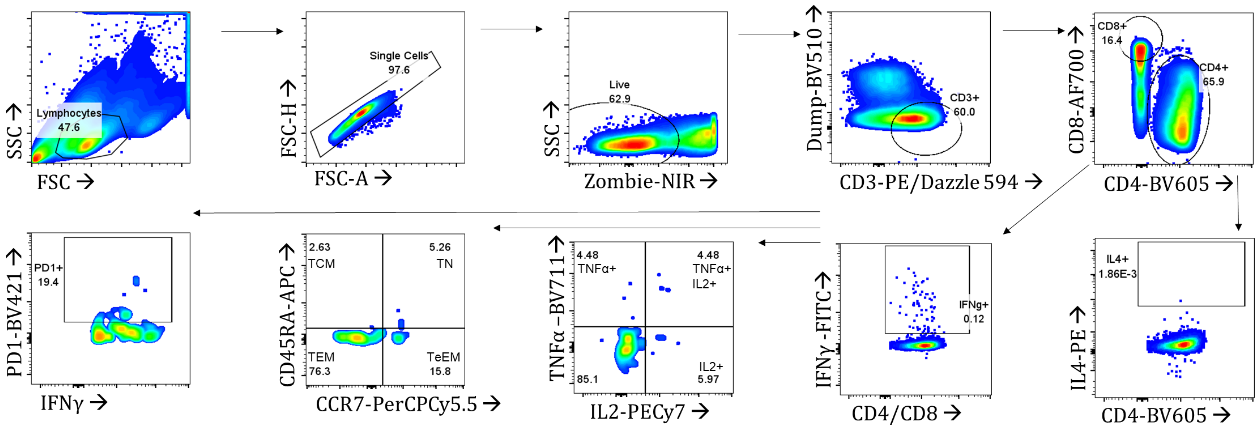

Figure S10. Representative FACS plots of IFNγ production by $CD4^+$ and $CD8^+$ T cells, and IL4 production by $CD4^+$ T cells, at baseline (Day 0), Day 7 and Day 28 after randomised third-dose CoronaVac or BNT162b2 vaccination, of (A) responders and (B) non-responders to SARS-CoV-2 structural proteins stimulation, and of (C) buffy coat samples from healthy donors to control stimulations. A subset of 20 participants were randomly selected from participants who agreed to provide PBMCs samples from each study arm for stimulation by overlapping peptide pool representing the SARS-CoV-2 structural proteins (spike, nucleocapsid, envelope and membrane) and then subjected to intracellular cytokine staining. Representative FACS plots of responders were shown for each study arm, and FACS plots of non-responders were representative of all arms. Representative FACS plots of buffy coat samples from separate uninfected and unvaccinated healthy donors stimulated by PMA/Ionomycin (positive control) or DMSO (negative control) were also included. For each sample, the (paired) IFNγ background response from DMSO stimulation was subtracted from the IFNγ peptide response from SARS-CoV-2 peptides stimulation to give the peptide-specific IFNγ response. We defined the threshold specific to that response as the lowest positive value from all samples collected from all days (0/ 7/ 28) (value for CD4+ IFNγ+ T cells: 0.001%; CD8+ IFNγ+ T cells: 0.00017; CD4+ IL4+ T cells: 0.000042%). A sample with a value larger than this threshold was classified as having a positive response, i.e. a "responder", otherwise was classified as "non-responder". FACS: Fluorescence-activated cell sorting.

(Continued to next page)

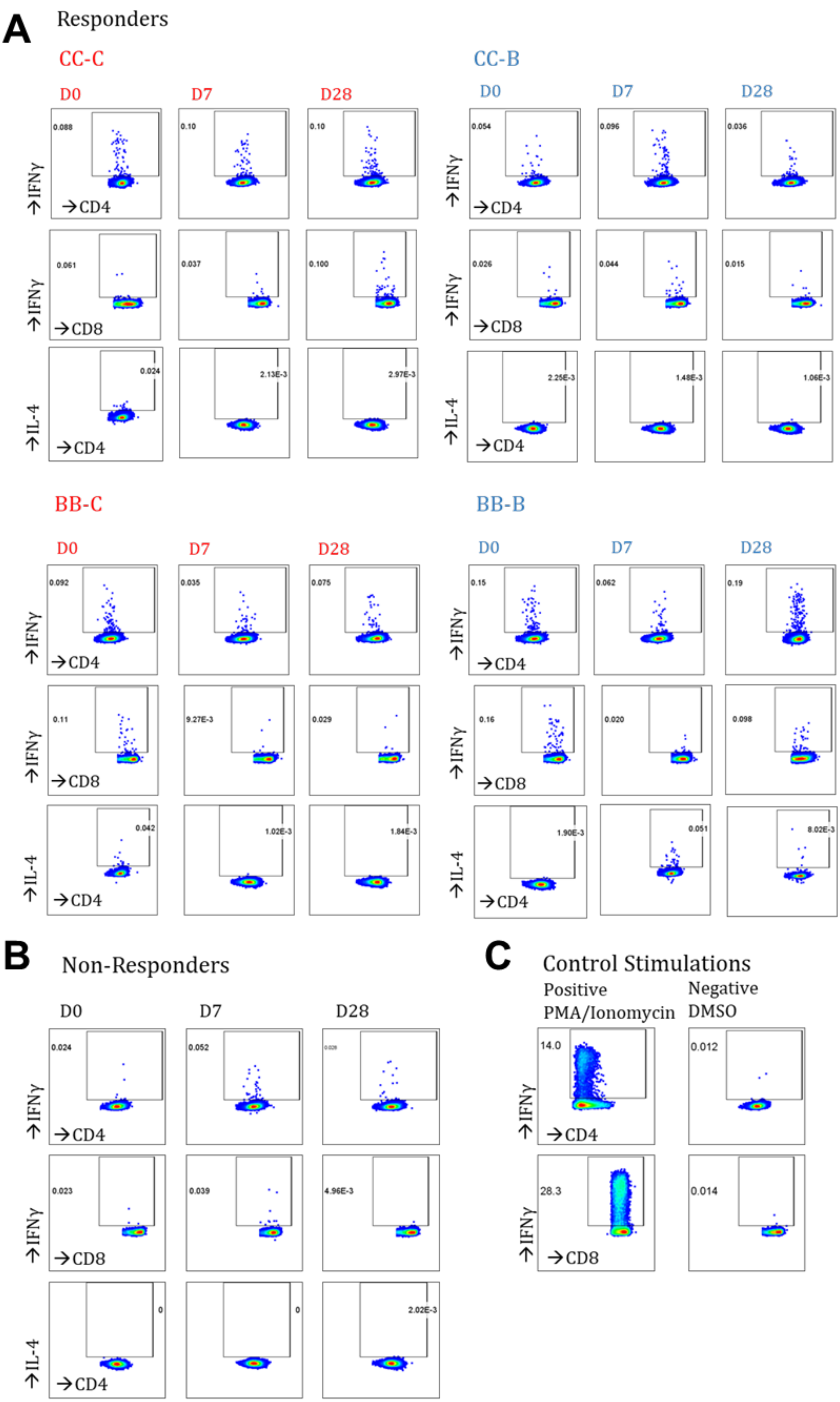
A
Responders
CC-C
CC-B
BB-C
BB-B
D0
D7
D28
→IFNγ
→IL-4
→CD4
→CD8
0.088
0.10
0.10
0.054
0.096
0.036
0.061
0.037
0.100
0.026
0.044
0.015
0.024
2.13E-3
2.97E-3
2.25E-3
1.48E-3
1.06E-3
0.092
0.035
0.075
0.15
0.062
0.19
0.11
9.27E-3
0.029
0.16
0.020
0.098
0.042
1.02E-3
1.84E-3
1.90E-3
0.051
8.02E-3
B
Non-Responders
0.024
0.052
0.023
0.039
4.96E-3
0
0
2.02E-3
C
Control Stimulations
Positive
PMA/Ionomycin
Negative
DMSO
14.0
0.012
28.3
0.014

Figure S11. Poly-cytokine production (TNFx and IL-2) and memory phenotype in IFNγ-producing (A) CD4+ and (B) CD8+ T cell responses against structural peptides of ancestral SARS-CoV-2 virus, at baseline, 7, and 28 days after randomised third-dose CoronaVac or BNT162b2 vaccination. The four study arms included participants who previously received two-dose CoronaVac and were randomised to receive a third dose of CoronaVac ("CC-C") or BNT162b2 ("CC-B"), and participants who previously received two-dose BNT162b2 and were randomised to receive a third dose of CoronaVac ("BB-C") or BNT162b2 ("BB-B"). Data was available from a random subset of 20 participants selected from each study arm. The grey lines represent percentages of IFNγ-producing cells from 0% to 100% with increments of 25%, and the position of measurement on line represents the mean value of the 20 participants. Tn: naïve T cells, Tcm: central memory T cells, Tem: effector memory T cells, Teem: terminal effector memory T cells.

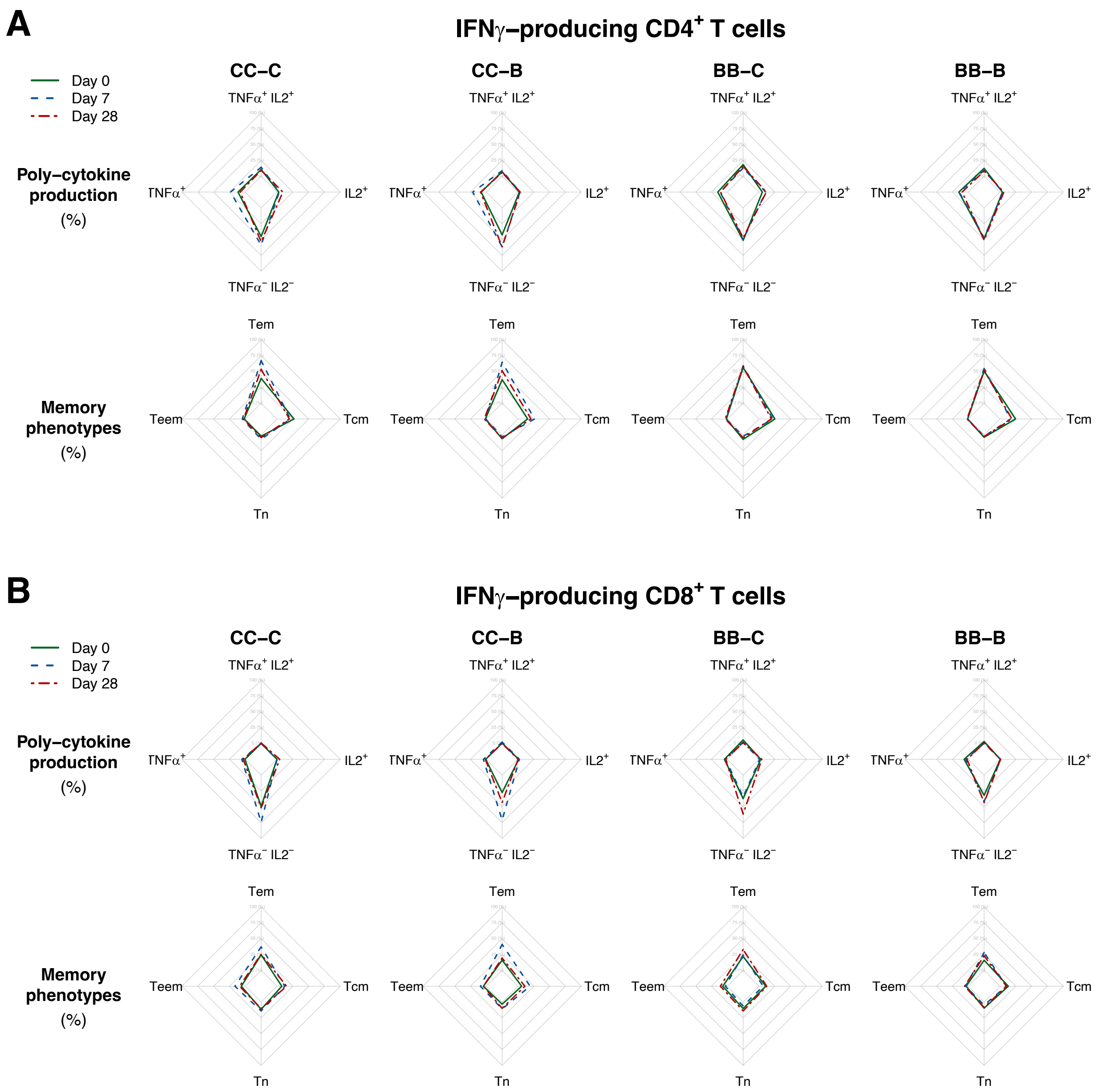

Study Protocol

**Abbreviated Title**	**Cobovax study**
**Protocol Title**	**Randomized trial of COVID-19 booster vaccinations (Cobovax study)**
**Study Phase**	IV
**Intervention/Treatment**	One booster dose of BNT162b2 (Comirnaty®) or CoronaVac
**Manufacturer**	BioNTech/Fosun Pharma, Sinovac
**Protocol No (HKG)**	BJC053
**Version No**	3.0
**Version Date**	7 April 2022
**Grant/Project No.**	Food and Health Bureau and Health and Medical Research Fund (Ref. No. COVID19F09)
**Sponsor**	The University of Hong Kong
**ClinicalTrials.gov Identifier**	NCT05057169
**Indication**	Adults who received two doses of CoronaVac (Sinovac) or two doses of BNT162b2 (BioNTech/Fosun Pharma) at least 6 months earlier

1 INVESTIGATORS CONTACT INFORMATION

**Principal Investigator:**	Prof. Benjamin J. Cowling (PI)<br>Dr. Nancy H. L. Leung (co-PI)
**Institute:**	School of Public Health, Li Ka Shing Faculty of Medicine<br>The University of Hong Kong
**Address:**	Patrick Manson Building<br>7 Sassoon Road, Pokfulam, HKG
**E-mail:**	bcowling@hku.hk<br>leungnan@hku.hk
**Contact No:**	+852 3917 6711<br>+852 3917 6757
**Co-Investigators:**	Pof. J. S. Malik Peiris<br>Dr. Sophie Valkenburg<br>Dr. Mario Martín-Sánchez<br>Dr. Dennis K. M. Ip

2 SIGNATURE PAGE

The signature below provides the necessary assurance that this trial will be conducted according to all stipulations of the protocol, including all statements regarding confidentiality, and according to local legal and regulatory requirements and applicable US federal regulations and ICH E6 Good Clinical Practice (GCP) guidelines.

I agree to conduct the study in compliance with GCP and applicable regulatory requirements.

I agree to conduct the study in accordance with the current protocol and will not make changes to the protocol without obtaining the sponsor's approval and IRB/IEC approval, except when necessary to protect the safety, rights, or welfare of subjects.

Site Investigator Signature:

Signed: ______________________________ Date: ______________

Name: Benjamin J. Cowling

Title: Chair Professor of Epidemiology

3 SUMMARY OF PROTOCOL CHANGES

Changes to the protocol since version 1.0 are summarized in the following table:

Number	Date	Type	Brief Details
2.0	2021-Nov-04	Minor	Revised to:<br>- Change in terminology from "booster dose" to "3rd dose" since future booster doses (e.g. 4th dose, 5th dose etc.) are not assessed in this study<br>- Clarification of the permuted block design for the randomization sequence (section 5.1)<br>- Addition of Day 7 assessment (section 5.2.5)<br>- Collection of additional blood samples in a subset of participants for assessment of cellular immunity (section 5.3)<br>- Clarification that participants are only eligible if they have received two doses of BioNTech/Fosun vaccine or two doses of Sinovac vaccine, but not any other COVID-19 vaccines, listed in the second exclusion criteria in (section 6.2)<br>- Inclusion of information on financing and insurance for this study (section 18)<br>- Minor edits for clarification throughout, and addition of additional background information, with no substantive change to any specific procedure or process in the trial
3.0	2022-Apr-07	Major	Revised to:<br>- Addition of new research activities: Durability of immunity; Active surveillance for illness (section 2)<br>- Addition of background and rationale for new research activities: Durability of immunity; Active surveillance for illness (section 3)<br>- Addition of secondary objectives for new research activities: Durability of immunity; Active surveillance for illness (section 4.2)<br>- Addition of requesting additional informed consent from participants for new research activities: Durability of immunity; Active surveillance for illness (section 5.2.3)<br>- Collection of additional blood samples at additional time points in CMI participants for assessment of durability of immunity (section 5.3)<br>- Addition of new section describing the study procedure for Active surveillance for illness (section 5.5)<br>- Addition of secondary outcomes for new research activities: Durability of immunity; Active surveillance for illness (section 8.2)<br>- Additional details describing blinding of participants and study team (section 10.2)<br>- Additional details describing the laboratory methods for assessing cell-mediated immune responses (section 10.2)<br>- Addition of new section describing the laboratory methods for assessing host genetics (section 10.3)

- Addition of new section describing the laboratory methods for assessing systems serology (section 10.4)
- Clarification on data cleaning for missing data (section 12.2)
- Addition of details describing risk associated with self-collection of respiratory swabs for rapid antigen tests (section 15)
- Removal of details and section on monitoring by Data Monitoring Committee as both third-dose homologous and heterologous vaccination is emergency-approved and recommended by local government policy (section 15)
- Addition of new section describing biological sample handling and keeping in accordance to the Informed Consent obtained (section 16)
- Additional details describing data entry and anonymized data sharing with local and international collaborators (section 17)
- Minor edits for clarification throughout
- Addition of the following study documents:
  - Appx 13. Additional Informed Consent – Durability of immunity
  - Appx 14. Additional Informed Consent – Active surveillance for illness
  - Appx 15. Active surveillance Initiation
  - Appx 16. Active surveillance Form
  - Appx 17. Illness Symptom Diary
  - Appx 18. Illness Recovery Questionnaire

4 PROTOCOL SUMMARY

**Background:**	The accrual of population immunity to COVID-19 could allow life to return to pre-pandemic normality. Immunity can be acquired through natural infections or, preferably, by vaccination. An unprecedented global effort has succeeded in developing a number of COVID-19 vaccines. All vaccines against COVID-19 approved until now have originally been developed as either a single dose or following a homologous two-dose regimen. Inactivated COVID-19 vaccines have shown inferior immunogenicity compared to mRNA vaccines but there are no studies comparing the advantages of alternative 3rd doses in individuals who have previously received two doses of an inactivated COVID-19 vaccine or two doses of an mRNA vaccine.
**Aims and primary objectives:**	The aims of this study are: (1) to compare the SARS-CoV-2 antibody responses to one dose of BNT162b2 (mRNA vaccine, BioNTech/Fosun Pharma) versus one dose of CoronaVac (inactivated vaccine, Sinovac) in individuals who have previously received two doses of COVID-19 vaccination using BNT162b2 (mRNA vaccine, BioNTech/ Fosun Pharma) or CoronaVac (inactivated vaccine, Sinovac), and (2) to assess the reactogenicity and safety of homologous or heterologous 3rd dose of vaccination. The specific primary objective of our study is to assess the vaccine (humoral) immunogenicity, proxied by SARS-CoV-2 serum neutralizing antibody titers, of a 3rd dose of BNT162b2 or CoronaVac at 28 days after the 3rd dose in individuals who have previously received a homologous two-dose primary series of BNT162b2 or CoronaVac.
**Study design:**	Randomized open label trial in adults aged 18 years of age or older (at enrolment). The duration of participation for each participant will be 12 months from the administration of the 3rd vaccine dose. We will investigate the immune response and reactogenicity of one dose of BNT162b2 or CoronaVac in individuals who previously received two doses of inactivated or mRNA COVID-19 vaccines at least 6 months earlier. Participants will be enrolled shortly before receiving the 3rd dose of BNT162b2 or CoronaVac (day 0), with blood collection at days 0, 28, 182 and 365 days after enrolment for analysis of humoral immune responses. A subset of 25% of participants will provide (optional) additional blood samples at day 0, 7, 28, 182 and 365 for assessment of cellular immune responses and durability of immunity. To assess potential vaccine effectiveness, during period with considerable local COVID-19 activities, we will invite all participants to participate in (optional) active surveillance for illness. We will invite participants for additional blood collection and adverse monitoring for each subsequent COVID-19 vaccine dose that they may receive elsewhere.
**Main outcomes:**	The primary outcome is the vaccine (humoral) immunogenicity measured as SARS-CoV-2 serum neutralizing antibodies, evaluated as the geometric mean titer (GMT) at 28 days after the 3rd doses. The secondary outcomes include (1) a comparison of SARS-CoV-2 serum neutralizing antibodies as the geometric mean fold rise from baseline to each post-vaccination timepoint (i.e. at days 28, 182 and 365); (2) a comparison of cellular immune responses at day 7 and 28 compared to day 0; (3) descriptive analysis of the reactogenicity and safety profiles of the 3rd doses.
**Target Population**	Adults aged 18 years or older
**Number of Subjects Planned**	400 participants to be recruited in 2021-22

**Study Duration**	12 months, from December 2021 through to June 2023
**Participant Duration**	12 months follow-up for each participant
**Potential implications:**	Our study will provide important evidence into the comparative effects of using a 3rd dose of mRNA vaccine or inactivated vaccine to boost the immune response in individuals that had previously received two doses of mRNA or inactivated COVID-19 vaccines. This information together with data collected on reactogenicity and safety could inform COVID-19 vaccination policy locally and internationally.

5 BACKGROUND

The recent emergence of the new coronavirus disease 2019 (COVID-19) in late 2019 and the subsequent global pandemic has since caused 165 million cases and 3.4 million deaths globally by 20 May 2021, as well as significant economic impacts as an observed 4% global GDP reduction in 2020 compared to 2019. SARS-CoV-2 infections vary widely in disease severity from asymptomatic infection, mild self-limiting infections with common respiratory symptoms, through to severe disease requiring hospitalization that could even lead to death. Studies suggested older adults and individuals with comorbidities such as hypertension, obesity and diabetes have higher risk of severe disease including hospitalization and deaths.

The accrual of population immunity to COVID-19 will allow life to return to normal. Immunity can be acquired through natural infections or, preferably, by vaccination. An unprecedented global effort has been concerted to develop COVID-19 vaccines (1). Within one year since the emergence of the virus, the BioNTech/Pfizer 2-dose mRNA vaccine BNT162b2 (trade name 'Comirnaty') was granted emergency-use approval in the United Kingdom on 2 December 2020. The United States Food and Drug Administration (FDA) granted emergency use approval to the BioNTech/ Pfizer vaccine on 11 December as well as the Moderna 2-dose mRNA vaccine mRNA-1273 on 18 December. Since then, other vaccines that use diverse vaccine platforms have received partial or full approval across the world, including Oxford/AstraZeneca 2-dose ChAdOx1 vector-based vaccine AZD1222 that granted emergency authorization from United Kingdom and Argentina on 29 December, or 1-dose CanSino vector-based vaccine Ad5-nCoV and 2-dose Sinovac inactivated vaccine CoronaVac approved for use in China.

The COVID-19 vaccination programme started in Hong Kong in February 2021. The programme offers each adult in Hong Kong a choice of Sinovac 2-dose inactivated vaccine CoronaVac (2 doses on average 28 days apart) or BioNTech/ Fosun Pharma/Pfizer 2-dose mRNA vaccine BNT162b2 (2 doses 21 days apart), with broad eligibility criteria and availability. Up to 21 October 2021, 68% of the eligible population has received at least one dose of either vaccine and a total of 5.7 million doses of BNT162b2 and 3.3 million doses of CoronaVac have been administered.

Safety and effectiveness are the main aspects evaluated before vaccines are licensed. Results from clinical trials have shown that COVID-19 vaccines, despite the heterogeneity in design and development, have a safety profile similar to other viral vaccines (2-4). Besides common local reactions, for the BNT162b2 vaccine the most common adverse reactions reported in phase II/II trials were fatigue and headache (2). Although about 4% of recipients presented severe fatigue and 2% severe headache, symptoms were most of the time mild-to-moderate and resolved with a week (2). Fever was reported by more than 10% of the vaccine recipients and lymphadenopathy was presented by 64 (0.3%) vaccine recipients (2). For Sinovac's CoronaVac vaccine, results from phase II show that most adverse reactions were mild and participants recovered within a few days (4), and interim results of a double-blind, randomized, placebo-controlled phase III trial in Turkey with >11000 participants aged 18-59 years suggested good tolerability profile of CoronaVac, with any adverse events of 18.9% versus 16.9% in the CoronaVac versus placebo group respectively, with no fatalities or grade 4 adverse events (5). A double-blind, randomized, placebo-controlled phase I/II trial in China of 422 healthy older adults ≥ 60 years old receiving CoronaVac at different doses (1.5μg, equals to 300SU (Spike Unit); 3μg/600SU; 6μg/1200SU), reported that in those receiving 3μg/600SU, 20% reported

mild/moderate adverse reactions within 28 days after infection, compared to 21% in the placebo group, and only one serious adverse event (SAE) in the 3µg/600SU group which was deemed unrelated to vaccination (6). A recently published study evaluated the risk of Bell's palsy within 42 days after BNT162b2 (in aged ≥ 16 years old) or CoronaVac (in aged ≥ 18 years old) vaccination in Hong Kong, using local data from voluntary surveillance reporting with the Hospital Authority, the COVID-19 Vaccine Adverse Event Online Reporting system for all health-care professionals, and the Hospital Authority's territory-wide electronic health records from the Clinical Data Analysis and Reporting System (7). From 451,939 individuals who have received first dose of CoronaVac and 537,205 who have received first dose of BNT162b2, the age-standardised incidence of clinically confirmed Bell's palsy was 66.9 cases per 100 000 person-years (95% CI 37.2 to 96.6) following CoronaVac vaccination and 42.8 per 100 000 person-years (19.4 to 66.1) for BNT162b2 vaccination. The age-standardised difference for the incidence compared with the background population was 41.5 (95% CI 11.7 to 71.4) for CoronaVac and 17.0 (-6.6 to 40.6) for BNT162b2, equivalent to an additional 4.8 cases per 100 000 people vaccinated for CoronaVac and 2.0 cases per 100 000 people vaccinated for BNT162b2, with about 2.4-fold significant increase in risk of Bell's palsy after CoronaVac vaccination and about 1.8-fold non-significant increase after BNT162b2 vaccination compared to the background population; however, the authors noted that beneficial and protective effects of the inactivated COVID-19 vaccine far outweigh the risk of this generally self-limiting adverse event. While in clinical trials the Oxford/AstraZeneca ChAdOx1 AZD1222 vaccine showed an acceptable safety profile, and the number of serious adverse events were similar in the vaccine and the control arm (3), an increase of the incidence in venous thromboembolic events has been found after the its emergency use in several European countries. Nevertheless, the European Medical Agency concluded that the risk was low and the benefits far outweigh its risks for most age groups (8). Due to the public health emergency, the speed of development and approval of COVID-19 vaccines have been unprecedented, which may have risen public concerns about their safety. Surveys conducted New Zealand, Australia and United States have shown that the main concerns and reasons for unwillingness to get vaccinated are in relation to safety and potential side effects (9).

The emergence of variants of concern (VOCs) and observed decrease in vaccine-induced immune responses within few months after vaccination have led to the discussion of the potential need of booster vaccination in the near future. So far there are limited studies on the use of third (booster) dose of mRNA or inactivated COVID-19 vaccines in healthy (10-13) or immunocompromised individuals (14, 15). For mRNA booster vaccination, Israel is one of the populations with highest vaccination coverage in the world and used mRNA vaccines (both Pfizer/BioNTech and Moderna) in their nation-wide vaccination programme; in August 2021, the Israeli government recommended individuals aged 60 years or older who received the second dose at least 5 months earlier to receive a (homologous) booster dose. An observational study of 346 healthcare workers who received a third dose of BNT162b2 (Pfizer/BioNTech) per the Israeli government's recommendation showed that most had about 64-fold increase in anti-SARS-CoV-2 IgG 10 days after the booster vaccination with no SAEs reported (10). Another study in Israeli using health system data suggested the rate of confirmed COVID-19 infection was about 11-fold and the rate of severe illness was about 19-fold lower in those who received a booster dose at least 12 days earlier compared to those who were not (16). Separately, in an open-label phase 2a trial in the US to evaluate the safety and immunogenicity of a third dose of mRNA-1273 (Moderna) or variant-modified multivalent mRNA-1273.211,

based on data of 20 mostly older adults in each group, neutralizing antibodies against wild-type D614G virus had waned before the third dose from the primary series about 6 months ago, and in particular neutralizing titer against B.1.351 (Beta), P.1 (Gamma) and B.1.617.2 (Delta) VOCs were either low or undetectable (11). After mRNA-1273 or mRNA-1273.211 booster vaccination 6 months after the second dose, neutralizing titers had either significantly increased compared to the peak titer 1 month after the primary series against wild-type D614G virus, or at least at a statistically equivalent level against the three VOCs (11). Both the mRNA-1273 and variant-modified boosters were safe and well-tolerated, with 15% or less who reported grade 3 (i.e. significant interference which prevents daily activities) solicited local or systemic adverse reactions (ARs) and no grade 4 (i.e. requires emergency room visit or hospitalization) solicited ARs after booster vaccination (11). Studies in a small number of organ transplant recipients suggested antibody titers increased after third dose and the vaccination reactions apparently to be acceptable with no severe adverse events observed (14). For inactivated vaccines, a placebo-controlled, double-blind phase II trial of CoronaVac vaccination in 540 participants aged 18 – 59 years old is being conducted in China, where participants are randomly allocated in 1:1:1:1 ratio to one of four schedules to receive a third dose (28 days or 6 months after two two-dose regimens either 14-day or 28-day apart, including: schedule 1 – days 0, 14, 42; schedule 2 – days 0, 14, 194; schedule 3 – days 0, 28, 56; schedule 4 – days 0, 28, 208), and simultaneous randomisation in 2:2:1 ratio to two vaccine-dose/placebo group (high-dose group – 6μg; medium-dose group – 3μg; placebo group) (13). Based on preliminary results reported in a pre-print article, in the medium-dose (3μg) group, neutralizing antibody titers declined to below the seropositive cutoff 5-8 months after the second dose, but administering a third dose 6 months later increased the GMT titer to 144, with only grade 1 or 2 solicited local and systemic adverse reactions reported within 28 days after receiving the third dose. For other vaccine platforms such as vector-based vaccines, in a substudy of a phase 1/2 controlled trial (COV001) of AZD1222 in the UK which provided a third dose 28 – 38 weeks after the second dose in about 70 participants aged 18 – 55 years and who had reactogenicity and immunogenicity data available, it showed that antibody titres were significantly higher 28 days after a third dose compared to 28 days after the second dose (median total IgG titer 3746 EUs versus 1792 EUs respectively), and reactogenicity after a third dose was lower than that after a first dose (12).

All vaccines against COVID-19 approved until now have originally been developed as either a single dose or following a homologous two-dose regimen as primary series. Only few studies are being conducted to assess the immunogenicity and reactogenicity of a vaccine mixing regimen as a heterologous prime-boost strategy in primary series (17-21) and no studies on the use of a different vaccine platform as a booster dose to a complete homologous 2-dose regime. However, administering a third/ booster dose of a different vaccine platform could have advantages: 1) they could allow a flexible immunisation schedule given distribution challenges of vaccines at a global scale, and 2) the combination of vaccines using different vaccine platforms or targeting diverse viral elements may enhance the immune response and, eventually, increase its effectiveness. Inactivated covid-19 vaccines have shown an inferior immunogenicity, even with the use of adjuvants compared to mRNA vaccines (22). T-cell responses induced by inactivated vaccine has also been reported to be minimal (4, 23). There are no published studies to-date yet looking into the immunogenicity, reactogenicity and safety profile of an mRNA vaccine in individuals who have previously received an inactivated COVID-19 vaccine, but it is foreseeable that the administration of mRNA vaccine as a booster to

inactivated vaccine may further improve the vaccine immunogenicity when compared to vaccination with inactivated vaccine alone, or may even provide more advantage when compared to booster dose using inactivated vaccine. Conversely, one may consider receiving an inactivated vaccine as a booster to primary series of mRNA vaccines for presumably lower reactogenicity.

Recent studies and our preliminary data suggested that antibody response start to decrease shortly after COVID-19 vaccination (24, 25), while there are conflicting evidence about the persistence of cell-mediated immune responses (26-28). In a study of SARS-CoV-2 antibody waning within 6 months after vaccination with the 2-dose BioNTech vaccine, it was observed that GMT for IgG or neutralizing antibody peaked at about one month after vaccination, with substantial decrease in IgG over the next 6 months (by a factor of 18.3), but less so in neutralizing antibodies (decreased by a factor of 3.9 from the 1st to 2nd month, and by a factor 1.2 from the 2nd to 6th month); and significantly lower neutralizing antibody titers were associated with older age, male sex, and the presence of immunosuppression (29). Our preliminary data on the vaccine effectiveness locally after one, two and three doses of COVID-19 vaccines also suggest breakthrough infections after infection for all vaccine types particularly by the VOCs such as the Omicron variant (30). A comprehensive evaluation of short-term vaccine-induced immunity as well as health outcomes will inform the potential contribution of different combinations of homologous or heterologous COVID-19 vaccination to reducing susceptibility to infection or severe disease as well as the duration of vaccine-induced immune responses.

Here, we propose to compare the immunogenicity and reactogenicity to a 3rd dose of an mRNA vaccine (BNT162b2, also known as BioNTech/ Fosun Pharma-Comirnaty locally) or an inactivated vaccine (Sinovac-CoronaVac) in individuals that had previously received two doses of mRNA or inactivated COVID-19 vaccination, the durability of vaccine-induced antibody and cell-mediated response, as well as the potential vaccine effectiveness against COVID-19 infection. Our results will inform vaccination policy locally and internationally, specifically on the potential benefits of 3rd doses using the same or different vaccine platforms to the primary series. Secondary laboratory and data analyses will also allow in-depth investigation of the interaction between host genetics, different branches of immune responses and vaccine types.

Previous clinical trials by our research group

Our research group is experienced in conducting research on COVID-19 and other respiratory viruses of public health importance including large randomized controlled trials of influenza vaccines (31-34), with expertise across infectious disease epidemiology, virology, immunology and biostatistics. The research team is well staffed with GCP-trained research personnel, health care practitioners and phlebotomists experienced in conducting community-based studies, laboratory technicians in a world-recognized team on coronavirus research equipped with a biosafety level-3 laboratory, and statisticians experienced in analyzing similar data. For this study, both CoronaVac and BNT162b2 are already approved for use in Hong Kong and made freely available to adults through the Hong Kong government's COVID-19 Vaccination Programme.

6 AIMS AND OBJECTIVES

The aims of this study are: (1) to determine whether one dose of BNT162b2 (mRNA vaccine, Fosun/BioNTech) or one dose of CoronaVac (inactivated vaccine, Sinovac) can boost neutralizing antibodies against SARS-CoV-2 in individuals who have previously received two doses of COVID-19 vaccination, and (2) to assess the reactogenicity and safety of homologous or heterologous 3rd dose of vaccination.

Primary objective

The specific primary objective of our study is to assess the vaccine (humoral) immunogenicity, proxy by SARS-CoV-2 serum neutralizing antibody titers, of a 3rd dose of BNT162b2 or CoronaVac at 28 days after the 3rd dose in individuals who have previously received a homologous two-dose primary series of BNT162b2 or CoronaVac.

Secondary objectives

The specific secondary objectives of our study are:

1. To assess the maintenance of SARS-CoV-2 serum neutralizing antibody response after a 3rd dose of BNT162b2 and CoronaVac at 28, 182 and 365 days after enrolment in individuals who have previously received a homologous two-dose primary series of BNT162b2 or CoronaVac.
2. To assess cellular immune responses after a 3rd dose of BNT162b2 and CoronaVac at day 7 and 28 compared to day 0.
3. To characterize the possible local and systemic adverse events by describing the immediate and delayed vaccine-induced events within 28 days after a 3rd dose of BNT162N2 or CoronaVac, in individuals who have previously received a homologous two-dose primary series of BNT162b2 or CoronaVac.
4. To assess the maintenance of cellular immune responses after a 3rd dose of BNT162b2 and CoronaVac at 28, 182 and 365 days after enrolment in individuals who have previously received a homologous two-dose primary series of BNT162b2 or CoronaVac.
5. To identify breakthrough infections and assess the potential vaccine effectiveness of homologous or heterologous third-dose COVID-19 vaccination against SARS-CoV-2 infection.

7 STUDY DESIGN & STUDY PROCEDURE

Study design

This study is an open-label randomized trial in adults aged 18 years or older (at enrollment) to measure the vaccine (humoral) immunogenicity and reactogenicity of boosting with a mRNA vaccine (BNT162b2) or an inactivated vaccine (CoronaVac) in individuals who have previously received two doses of either BNT162b2 or two doses of CoronaVac (**Figure 1**). The duration of participation for each participant will be 12 months from the administration of the 3rd dose of BNT162N2 or CoronaVac **(Figure 2)**.

In this study, invitations to participate will be extended to community-dwelling adults in Hong Kong through mass promotion efforts including advertisements in newspapers, transportation and social media platforms (such as Facebook and Instagram), study website, bulk university emails, mass mailing to residential estates, and invitation to and referrals from members of existing cohorts. Interested adults will be invited to visit designated Community Vaccination Centres (CVC), DH/HA facilities or private clinics for the government's mass vaccination programme for screening and enrollment, and designated study research sites or clinics for other trial-related activities. We plan to enroll 400 adults and follow them up throughout the

study period for 1 year (365 days). Individuals will be eligible for this study if they have previously received a homologous two-dose primary series of either BNT162b2 or CoronaVac, with the second dose received at least 6 months (180 days) earlier. Participants who either received a primary series of BNT162b2 or CoronaVac will be randomized separately, and in each group they will be randomly assign to receive either a BNT162b2 (B) or CoronaVac (C) in 1:1 ratio, i.e. four randomized groups (BB-B; BB-C; CC-B; CC-C).

Figure 1. Design of the Cobovax study.

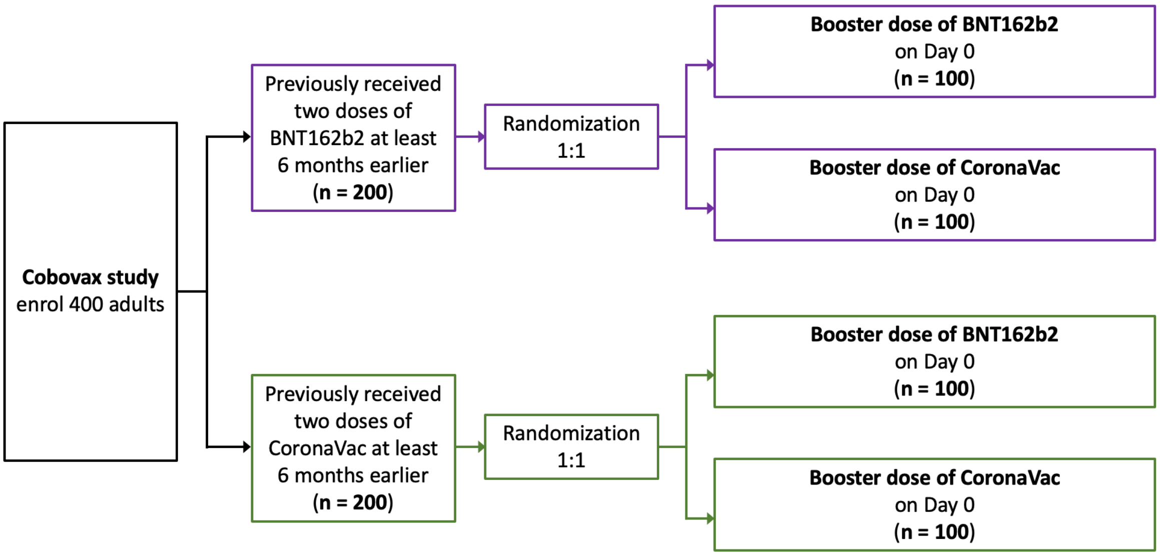

Figure 2. Study design and participant follow-up of the Cobovax study.

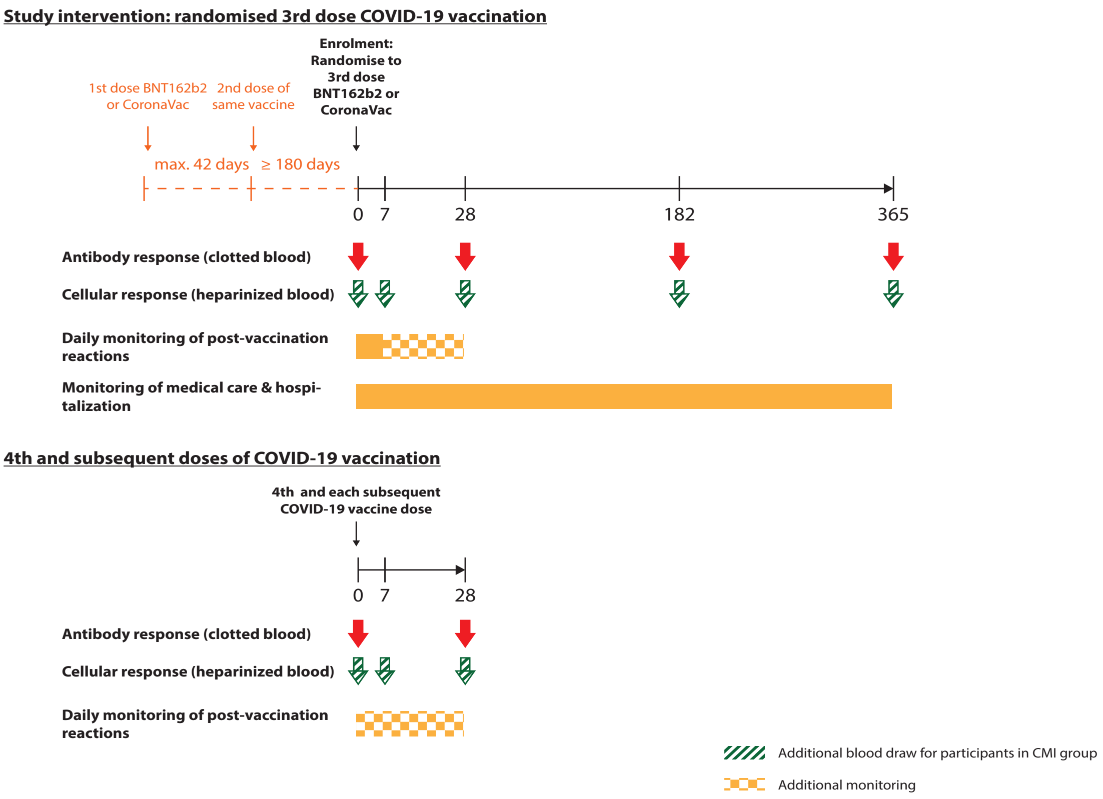

During the follow-up, we will collect clotted blood specimens at enrolment (day 0) and again after 28 days, 182 days and 365 days to assess the SARS-CoV-2 serum neutralizing antibody response (**Figure 2**). In a voluntary subset of 25% of participants (CMI group) we will collect additional whole blood samples at days 0, 7 and 28, and days 182 and 365 (if funding available) for analysis of cellular responses (**Figure 2**). In all participants, to characterize the possible local and systemic adverse events participants experience after the 3rd dose, participants will be requested to record solicited immediate and delayed vaccine-induced local or systematic events through an online e-diary, daily within the first 7 days after the 3rd dose, or until the last symptom disappears, whichever the later. Study personnel will contact the participants at days 28, 182 and 365 to confirm the presence or absence of events requiring medical attention within 28 days after the 3rd dose, and within about 6 and 12 months afterwards. To identify breakthrough infections and assess potential vaccine effectiveness, during period with considerable local COVID-19 activities, we will invite all participants to participate in (optional) active surveillance to identify acute respiratory illnesses (ARI). Throughout the study, participants will be interviewed at enrolment, 182 and 365 days after enrolment to provide health information such as existing medical conditions, vaccination history and hospitalization. If participants seek additional COVID-19 vaccination subsequently elsewhere (e.g. government's mass vaccination programme), we will invite participants to provide (optional) additional blood samples shortly before and about 28 days after each dose of vaccination, and (optional) monitoring of vaccine-induced local or systematic events daily within the first 7 days after the vaccination or until all symptoms resolve.

Study Procedures
The study procedure to be conducted for individual participant in the study are presented in tabular form in Table 1 and described in the text that follows.

Table 1. Study procedures.

**Cobovax study**	**1 year**					
**Procedure**	SCR	BSL Day[E]	Day	Day	Day	Day
		0	7	28	182	365
Demographics	X	X				
Medical History	X	X			X	X
Eligibility Criteria	X	X				
Informed Consent		X				
Height		X				
Weight		X			X	X
Vaccination Card		X				
Incentive		X	X [E]	X	X	X
**Health Questionnaires**						
Enrolment Assessment		X				
Mid-year Assessment					X	
End-of-study Assessment						X
Immediate local & systemic events [A]		X				
Delayed local & systemic events [B]			X			

Medical attention e.g. hospitalizations & serious adverse events [C]		X				
**Study intervention**						
Randomly assigned to receive either one dose of Comirnaty (BNT162b2) or one dose of CoronaVac vaccine		X				
**Blood draw (Vaccine immunogenicity laboratory assessment)**						
Clotted blood [D]		X [F]		X	X	X
Whole blood [E]		X [E, F]	X [E]	X [E]		
**Active surveillance for respiratory illness**						
Regular COVID-19 rapid antigen test [G]				X		

SCR: screening; BSL: baseline

A. On Day 0, after one dose of vaccination, patients will be observed for 30 minutes for immediate events.
B. E-diaries for daily monitoring of solicited immediate and delayed events will be provided from Day 0 to Day 7, with additional daily monitoring from Day 8 up to Day 28 or until all symptoms resolve, whichever the earlier, if reported any reaction on Day 7.
C. Study personnel will contact the participant at days 28, 182 and 365 to confirm the presence or absence of events requiring medical attention, including but not limited to serious adverse events (SAEs), i.e. within 28 days, about 6 and 12 months after the administration of study intervention respectively.
D. For all participants, during the visit, we will draw up to 20 ml of blood (serum) for serologic testing for antibody response. Using Clotted blood tube (RED), 9-10 ml per tube x 2.
E. For a subset of participants (CMI group), during the visit, we will draw additionally up to 20 ml of blood (whole blood) for testing for cell-mediated immune response. Using Heparin blood tube (GREEN), 9-10 ml per tube x 1 (Day 0, 7 and 28) AND (if funding available) 9-10 ml per tube x 2 (Day 182, 365).
F. Blood collection shortly before vaccination.
G. For a subset of participants, an optional active surveillance for respiratory illness will be conducted when there is substantial local COVID-19 activity. Participants will be given COVID-19 rapid antigen test (RAT) for self-test (depending on RAT availability) every 4 days and report the presence of any symptomatic illness.

Participant enrolment

In this study, invitations to participate will be extended to community-dwelling adults in Hong Kong through mass promotion efforts including advertisements in newspapers, transportation and social media platforms (such as Facebook and Instagram), study website, bulk university emails, mass mailing to residential estates, and invitation to and referrals from members of existing cohorts. Interested individuals will either complete an online application form, or call the study hotline, to sign up for screening for eligibility.

Screening

Screening for eligible adults may start 1-2 months before enrolment, and will continue until the target sample size is reached. Adults will be screened via telephone, or face to face at designed CVCs prior to enrolment, with a standardized questionnaire for assessment of eligibility, including questions on COVID-19 vaccination history, existing medical conditions, assessment of cognitive function and ability to provide informed consent (**Appendix 1: Recruitment Screening Questionnaire**).

Informed consent

Written informed consent will be sought from each eligible individual who is willing to participate in the study (**Appendix 2: Participant Information Sheet; Appendix 3:**

Participant Consent Form). Research personnel will explain the study in detail to the potential participants, including the study procedure and participants' risks, rights and benefits. Participation is voluntary, and the potential participants will be given time to ask questions about the study before providing written consent. For optional additional research activity on assessing durability of immunity, invitation will be extended to participants in the CMI group, and written informed consent will be sought from those who is willing to participate in the study activity (**Appendix 13: Additional Informed Consent – Durability of immunity**). For optional additional research activity on active surveillance for illness, invitation will be extended to all current participants, and written informed consent will be sought from those who is willing to participate in the study activity (**Appendix 14: Additional Informed Consent – Active surveillance for illness**).

Enrolment (Day 0) interview and assessment
The enrolment interview and blood draw will be completed to assess baseline characteristics in all participants. Participant will be asked to complete a standardized questionnaire via an online survey under the guidance of trained research personnel to collect information including demographics, body weight and height, medication use, vaccination history, medical conditions (chronic cardiovascular, pulmonary, renal, hepatic, metabolic, autoimmune and immunocompromising diseases or treatment) and other health-related information such as smoking status, general functional and health status and socioeconomical status (**Appendix 5: Enrolment Questionnaire**).

Day 7 assessment (in a subset only)
At day 7, a subset of participants who have elected to provided additional blood samples to evaluate cell-mediated immune response (CMI group) will return to our study research sites to provide blood samples.

Day 28 interview and assessment
At day 28, all participants will return to our study research sites to provide blood samples and will also complete a questionnaire about whether the participant has had any medical encounters since vaccination and specifically whether they have experienced any serious or unexpected Adverse Events Following Immunization (AEFI) (**Appendix 6: Post-vaccination Reaction Questionnaire**).

Mid-year (Day 182) interview and assessment
At day 182, a brief mid-year assessment and blood draw will be completed to assess potential changes in health status in the 6 months after vaccination in all participants, as well as to identify any additional COVID-19 vaccination that participants may seek elsewhere not via the study. A brief questionnaire on health information, including body weight, general health status, vaccination history, medical care including hospitalizations will be administered by trained research personnel (**Appendix 7: Mid-year Assessment Questionnaire**).

End-of-study (Day 365) interview and assessment
At day 365, an end-of-year assessment and blood draw will be completed to assess potential changes in health status in the 12 months after vaccination in all participants, as well as to identify any additional COVID-19 vaccination that participants may seek elsewhere not via the study. A standardized questionnaire similar to the baseline enrollment questionnaire will be administered by trained research personnel (**Appendix 8: End-of-study Assessment Questionnaire**).

Assessment for additional vaccine dose

As the fourth COVID-19 vaccine dose are available from the government's mass vaccination programme starting from April 2022, study participants may opt to receive 4th and subsequent doses outside our study. For these participants, we will invite participants to provide (optional) blood samples shortly before, 7 days and 28 days after each dose of vaccination to assess boost in immunogenicity.

Collection of blood samples

Blood draw by venepuncture will be performed by trained phlebotomists or nurses. Butterfly syringes and vacuum tubes will be used for participant comfort, and to allow filling in multiple blood tubes from a single venepuncture on some occasions for some participants. For clotted blood we will use tubes with clot activator and with or without separator gel (RED tube).

In all study participants, we will collect up to 20 ml clotted blood specimens at any time to assess vaccine immunogenicity and durability of immunity at days 0, 28, 182 and 365. The collected clotted blood will be stored in a refrigerated container at 2-8°C immediately and while in transit. Blood samples will be delivered to the laboratory at the University of Hong Kong as soon as possible, with the optimal delivery time within 24 hours after collection for further processing. Serum will be extracted from the clotted blood within 48 hours after collection, divided into 2-4 aliquots, and stored at -80°C until subsequent serologic testing.

In the CMI group, we will also collect heparinized blood specimens to assess cell-mediated immune (CMI) response, up to 10 ml at days 0, 7 and 28, and (if funding available) up to 20 ml at days 182 and 365. The collected heparinized blood will be kept at room temperature upon collection and while in transit. Blood samples will be delivered to the laboratory at the University of Hong Kong as soon as possible, with the optimal delivery time within 24 hours after collection for further processing. Peripheral blood mononuclear cells (PBMCs) will be isolated from the heparinized (whole) blood using Ficoll-Paque within 15 hours after collection, divided into 2 aliquots, and cryopreserved in liquid nitrogen until further analysis for CMI responses.

Table 2. Blood collection scheme for different participant groups.

Age group	General group	Cell-mediated immune response (CMI) group
**≥18 years old**	• 20ml clotted blood	• 20ml clotted blood<br>• 10-20ml heparinised whole blood

Adverse reaction monitoring

After study vaccination, participants will be observed for 15-30 minutes for immediate events. Participants will be provided with an e-diary or diary card (administered electronically daily) and requested to record any possible delayed events including local (e.g., pain, tenderness, swelling, erythema and itching) and systemic adverse events (e.g., feverish, fever, headache, fatigue, chills, muscle pain, nausea and others), and use of antipyretic or pain-relief medication for 7 days after each booster dose, or until the last symptom disappears, whichever the later (**Appendix 6: Post-vaccination Reaction Questionnaire**). A tympanic thermometer will also be provided to the participants for daily record of body temperature for 7 days after the booster dose. If the participant is still reporting any reaction on Day 7, additional daily monitoring from Day 8 up to Day 28 until all symptoms resolve will be conducted. The severity

of adverse reaction will be proxy by the overall impact of adverse events on daily activities. Alternatives e-diary will be provided based on the digital literacy of the participants such as paper-based diary or follow-up phone calls by the study team. If participants seek additional COVID-19 vaccination subsequently elsewhere (e.g. government's mass vaccination programme), we will invite participants to undergo (optional) monitoring of vaccine-induced local or systematic events daily within the first 7 days after the vaccination or until all symptoms resolve, whichever the later.

In addition, study personnel will assess the participants at days 28, 182 and 365 to confirm the presence or absence of events requiring medical attention, including but not limited to serious adverse events (SAEs), i.e. within 28 days after the booster dose of BNT162N2 or CoronaVac (**Appendix 6: Post-vaccination Reaction Questionnaire**), and within about 6 (**Appendix 7: Mid-year Assessment Questionnaire**) and 12 months (**Appendix 8: End-of-study Assessment Questionnaire**) afterwards respectively. SAE is any undesirable experience associated with the use of a medical product in participant when the participant outcome is either: 1) death; 2) life-threatening; 3) hospitalization; 4) disability or permanent damage; 5) required intervention to prevent permanent impairment or damage or 6) other serious event (important medical events). Fatal or life-threatening SAE will be reported to the local authorities within 7 calendar days from the first awareness by the Principal Investigator; for SAE other than fatal or life-threatening will be reported within 15 calendar days.

We expect very few if any vaccine-associated SAEs due to their rarity and the limited sample size of our study. However, if we do observe any SAEs (including severe allergic reactions like anaphylaxis, GBS or death) confirmed by a physician where appropriate, we will terminate participation for the participant and conduct an immediate investigation. Our research team will continue to follow up the terminated participants if they agree, same as those who withdraw with or without reason.

Active surveillance for acute respiratory illness

To identify SARS-CoV-2 infections, we will invite all participants to participate in an optional active surveillance for acute respiratory illness (ARI) during periods of considerable local COVID-19 activities. Research personnel will extend invitation to all participants by instant messages or telephone calls. For those participants who express interest in participating (**Appendix 14: Additional Informed Consent – Active surveillance for illness**), we will request the participants to self-perform COVID-19 rapid antigen test (RAT), and self-report the RAT and any other virologic test results, the presence of any respiratory illness and the details of the illness periodically via the online data collection platform REDCap. At the start of the active surveillance, participants are asked to report any RAT, virologic (PCR) test or symptom since 1 January 2022 (**Appendix 15: Active Surveillance Initiation**). Then, depending on the availability of COVID-19 RAT kits, we will arrange to mail participants COVID-19 RAT kits, so that participants can test themselves with the COVID-19 RAT on a regular basis every 4 days, and whether the test result is positive or not, report to the team through our questionnaire system and upload a photo of the test result (**Appendix 16: Active Surveillance Form**). Participants are also requested to report any other RAT or virologic test (such as mass testing or voluntary testing) results for COVID-19 or influenza virus, or if any symptoms appear. Research personnel will monitor real-time the progress of self-reporting by the participants, and send instant message reminders to participants who fail to report during the designated period. When any RAT or PCR test result is positive or any symptoms appear at present, participants are requested to complete the symptom diary (**Appendix 17: Illness Symptom Diary**) every day for at least 7 days and until all symptoms disappear, whichever is later, to

monitor disease severity, medication use and medical attention. When all symptoms resolve, or when the participant reports a resolved illness during active surveillance, the participant is asked to complete a questionnaire on medical care and medication use during the illness (**Appendix 18: Illness Recovery Questionnaire**).

Information on hospitalizations and deaths

If any hospitalizations or deaths are identified during the study, for example during longitudinal follow-up of health status or adverse reaction monitoring, we will collect additional information to evaluate the severity and potential causes of the severe health outcomes. For hospitalizations, participants or their proxies will be interviewed for details of the hospitalization including symptoms, duration of the hospitalization and discharge diagnosis (**Appendix 9: Hospitalization Questionnaire**). For deaths, participants who pass away during the study period will be identified and recorded through interview with the participant's family member or friend upon learning of the participant's death, or identified from hospital records or death certificates at the end of the study, with potential causes of death reported by the family member or friend, or abstracted from death certificates from Deaths Registry (**Appendix 10: Death Questionnaire**).

Incentives

Incentives will be provided in this study to compensate participants for their time, inconvenience, and discomfort, which is standard research practice for studies of this nature and we believe is necessary to maintain participation. Each participant will be offered an incentive of a gift voucher valued at HKD100 at each encounter for a blood draw (maximum 5 times in total). A tympanic thermometer will also be gifted to participant to encourage daily adverse event reporting including daily body temperature monitoring for 7 days after the booster dose.

Withdrawal criteria

Participants are free to withdraw their consent and participation at any stage of the study with no explanation and without any prejudice. If the participant chooses to withdraw, the reasons for withdrawal and participant health status will be recorded (**Appendix 11: Participant Withdrawal Form**). If participant wants to withdraw after having been administered the intervention, our research personnel will seek the participant's permission for monitoring the occurrence of a participant's adverse reaction (**Appendix 6: Post-vaccination Reaction Questionnaire**). The reasons for withdrawal and the participant's health status will then be recorded duly (**Appendix 11: Participant Withdrawal Form**). For participants who pass away during the study period, after the causes of death are recorded duly (**Appendix 10: Death Questionnaire**), the participant will be withdrawn from the study by research personnel (**Appendix 11: Participant Withdrawal Form**).

8 INCLUSION AND EXCLUSION CRITERIA

Potential participants will be eligible for this study if they meet the following inclusion criteria and meet none of the exclusion criteria, to be assessed before vaccination:

Inclusion criteria

1. Aged 18 years or older at enrolment.
2. Have received two doses of BNT162b2 OR two doses of CoronaVac, with the most recent dose at least six months (180 days) prior to enrolment.

3. Currently resident and planning to remain resident in Hong Kong during the duration of the study, i.e. for 12 months after enrolment.
4. Willing to provide blood samples for all the required time points.
5. The individual or their caregiver have a home phone or cellular or mobile phone for communications purpose.
6. Capable of providing informed consent.

Exclusion criteria
1. A history of laboratory-confirmed or clinically confirmed COVID-19 infection.
2. Have received (at least one dose of) any COVID-19 vaccines other than BNT162b2 or CoronaVac. **[Note 1]**
3. The two vaccine doses in the primary series were 43 days or more apart.
4. Individuals who report any medical condition, or as determined by a clinician, not suitable to receive mRNA or inactivated COVID-19 vaccines, including but not limited to allergies to the active substance or other ingredients of the vaccine. **[Note 2]**
5. Have any medical conditions related to their immune system as determined by a clinician.
6. Use of medication that impairs immune system in the last 6 months, except topical steroids or short-term oral steroids (course lasting ≤ 14 days).
7. Have used immunoglobulins and/or any blood products within 90 days prior to enrolment (administration of the study vaccine).
8. Currently pregnant, or planning, lactation or intention to become pregnant in the coming 3 months. **[Note 3]**

Note: [1] COVID-19 vaccines other than BNT162b2 or CoronaVac include (but not limited to) mRNA-1273 (mRNA vaccine, Moderna), BIBP (inactivated vaccine, Sinopharm), AZD1222 (adenovirus vector-based vaccine, Oxford/AstraZeneca), Sputnik V (adenovirus vector-based vaccine, Gamaleya Research Institute), Ad26.COV2.S (adenovirus vector-based vaccine, Johnson & Johnson) and ZyCoV-D (DNA vaccine, Zydus Cadila). **[2]** mRNA COVID-19 vaccine ingredients include ALC-0315, ALC-0159, DSPC, cholesterol, potassium chloride, potassium dihydrogen phosphate, sodium chloride, disodium phosphate dihydrate, sucrose, water for injection. Inactivated COVID-19 vaccine ingredients include inactivated SARS-CoV-2 virus, aluminium hydroxide, disodium hydrogen phosphate dodecahydrate, sodium dihydrogen phosphate monohydrate and sodium chloride. **[3]** At enrolment we will request enrolled female participants to report to us if they become pregnant during the study, and these participants will be withdrawn upon the reporting.

9 STUDY INTERVENTIONS

All injections with vaccines will be given through the intramuscular route by trained registered or enrolled nurses. Before vaccination, participants' vaccination history will be confirmed by asking participants and checking electronic vaccination records and/or vaccination cards (if applicable). After vaccination, the vaccine administered will be recorded on the study vaccination card (**Appendix 12: Vaccination Card**) to notify other clinicians of his/her study participation and vaccination.

Study Intervention Description
BNT162b2 is a nucleoside-modified mRNA encoding the trimerized SARS-CoV-2 spike glycoprotein. The vaccine is formulated in lipid nanoparticles that increase the efficiency of delivery of the mRNA into cells after intramuscular injection. BNT162b2 encodes the SARS-

CoV-2 full-length spike, modified by two proline mutations to lock it in the prefusion conformation and more closely recreate the intact virus with which the elicited virus-neutralizing antibodies interact. mRNA vaccines use the pathogen's genetic code as the vaccine; hence they exploit the host cells to translate the code and generate the target spike protein. The protein then acts as an intracellular antigen to stimulate the immune response of the vaccinated individual. The mRNA is then degraded within days. The vaccine was approved by the Secretary for Food & Health (SFH) in Hong Kong on 25 January. The first batch arrived at the end of February 2021 and was put into use in the COVID-19 Vaccination Programme on 10 March 2021.

CoronaVac is a Vero cell-based, aluminium hydroxide-adjuvanted, β-propiolactone-inactivated vaccine based on the CZ02 strain. This strain of SARS-CoV-2 was isolated from the bronchoalveolar lavage of a hospitalized patient and is closely related to the 2019-nCoV-BetaCoV Wuhan/WIV04/2019 strain. Each 0.5 ml dose is composed of 3 μg of inactivated SARS-CoV-2 virus. The excipients are aluminium hydroxide, disodium hydrogen phosphate, sodium dihydrogen phosphate, sodium chloride, and water for injection. The vaccine was approved by the Secretary for Food & Health (SFH) in Hong Kong on 18 February. The first batch arrived in late February 2021 and was put into use in the COVID-19 Vaccination Programme on 26 February 2021.

Randomization and blinding

The random allocation process will be concealed from both participants and the investigation team, using tamper-proof methods, by way of pre-assignment based on a computer-generated sequence of random numbers administered by clicking a randomization button in REDCap. A sequence of random numbers will be generated prior to the start of the study by a statistician. Computer software (R) will be used to generate the randomization sequence, using a block randomization structure with block sizes of 2, 4 and 6 for the enrollment of participants. Separate random allocation processes will be performed for participants who had previously received two doses of BNT162b2 and those who had previously received two doses of CoronaVac. We will randomly allocate each participant to receive a BNT162b2 or a CoronaVac in equal proportions.

This is an open-label study, where the participants and field investigation team will be aware of the vaccination regime received by the participants after the intervention is administered. Both the participants and field investigation team are not aware of the intervention before vaccination, as the vaccination is to be done by separate unblinded nurses at the community vaccination centres. After vaccination, participants will be provided with vaccination cards as required by the local government indicating which vaccine they have received, i.e. participants and field investigation team will be unblinded from this time onwards; however, laboratory investigation team performing all the laboratory testing will be blinded to the third-dose vaccine intervention at the time of testing as specimens will be coded with unique identifiers.

Description and handling of study vaccine

The interventions given through the intramuscular route are either: (1) BNT162b2 mRNA vaccine (Comirnaty®, BioNTech/Fosun Pharma), one dose (0.3mL after dilution) contains 30 micrograms of COVID-19 mRNA Vaccine embedded in lipid nanoparticles; or (2) CoronaVac inactivated vaccine (Sinovac), one dose (0.5mL in vial or syringe) contains 600SU of inactivated SARS-CoV-2 virus (CZ02 strain) as antigen and aluminum hydroxide as adjuvant.

Vaccines will be administered by staff of the vaccination centres, rather than our own study staff. Our field investigation team will not be responsible for handling or administering vaccines in this study.

Accountability
Principal investigator is responsible for supervising field work and liaising with the vaccination centre for the random assignment and administration of BNT162b2 or CoronaVac in study participants.

Return and Disposal
Our team will not be responsible for handling any vaccines directly.

Stopping rule
Given that all the products in the study are already in emergency use for COVID-19 prevention and participants will only be followed up for one year after receiving the booster dose, we do not plan to conduct any interim analyses to inform whether to continue the study.

According to two clinical studies that included 21,744 participants that have received at least one dose of BNT162b2, the most frequent adverse reactions were injection site pain (> 80%), fatigue (> 60%) and headache, and were usually mild or moderate in intensity and resolved within a few days after vaccination. Acute peripheral facial paralysis (or palsy) was rare (reported by four participants that receive the vaccine) (35). We observed during primary series, ARs following CoronaVac were of lower frequency than following BNT162b2 (unpublished data). An observational study of 346 healthcare workers who received a third dose of BNT162b2 (Pfizer/BioNTech) after primary series of 2-dose BNT162b2 showed that no SAEs were reported after the booster vaccination (10). In an open-label phase 2a trial in the US to evaluate the safety and immunogenicity of a third dose of mRNA-1273 (Moderna) or variant-modified multivalent mRNA-1273.211, based on data of 20 mostly older adults in each group, the mRNA-1273 were safe and well-tolerated with 15% or less who reported grade 3 (i.e. significant interference which prevents daily activities) solicited local or systemic ARs and no grade 4 (i.e. requires emergency room visit or hospitalization) solicited ARs after booster vaccination (11). For inactivated vaccines, a placebo-controlled, double-blind phase II trial of CoronaVac vaccination in 540 participants aged 18 – 59 years old in China, in the medium-dose (3μg) group, only grade 1 or 2 solicited local and systemic ARs were reported within 28 days after receiving the third dose. Heterologous prime-boost COVID-19 vaccination using BNT162b2 in individuals that previously received a viral vector vaccine (ChAdOx1 n Cov019, Vaxzebria AstraZeneca) showed a slight increase in some reactogenicity parameters compared to homologous vaccination. Nevertheless, reactogenicity symptoms were short lived, and no safety concern was drawn from the preliminary analysis (17).

Among our participants, we anticipate a rate of mild adverse events similar to previous studies using BNT162b2 and CoronaVac, hence we mostly expect mild and moderate reactions resolving within three days. However, if we observe any SAEs (including severe allergic reactions like anaphylaxis, GBS or death within the 7 days following vaccination) confirmed by a physician where appropriate, we will terminate participation for the participant and conduct an immediate investigation while suspending the trial until a satisfactory resolution.

10 OUTCOME MEASURES

Primary outcomes

The primary outcome measure is the vaccine (humoral) immunogenicity at 28 days after the 3rd dose of either BNT162b2 or CoronaVac, in individuals who have previously received two doses of BNT162b2 or CoronaVac, measured as geometric mean titer (GMT) of SARS-CoV-2 serum neutralizing antibodies against the vaccine strain (ancestral virus) using plaque reduction neutralization test ($PRNT_{50}$).

Secondary outcomes

The secondary outcome measures include:

1. The GMT of SARS-CoV-2 serum PRNT titers against the vaccine strain (ancestral virus) and potentially other VOCs at 182 and 365 days after the third-dose vaccination.
2. The geometric mean fold rise (GMFR) of SARS-CoV-2 serum PRNT titers against the vaccine strain (ancestral virus) and potentially other VOCs from baseline to post-vaccination timepoints (Day 28, 182 and 365).
3. Vaccine-specific IFN-γ+CD4+ and IFN-γ+CD8+ T-cell response at Day 7 and 28 after the third-dose vaccination.
4. Incidence of solicited local and systemic adverse events after the third-dose vaccination.
5. Incidence of hospitalizations during the year after the third-dose vaccination.
6. Incidence of COVID-19 infection during the year after the third-dose vaccination.

11 SAMPLE SIZE JUSTIFICATION

We propose that it is justified to enroll 400 participants into our study, including 100 in each randomized group (see **Figure 1** above). Our primary outcome is the GMT at day 28, compared between booster doses within each of the two strata (prior receipt of BNT162b2; prior receipt of CoronaVac). Based on our preliminary data, assuming $\log_{10}(GMT(PRNT_{50}$ at Day 0$))$ = log(27) = 1.4 with a standard deviation of 0.9, a sample size of 80 individuals per group will provide 80% power to detect a difference in log GMT of 0.4 or greater at the 5% significance level. The same calculation applies in each stratum. We include 100 per group to allow for the possibility of drop-outs prior to the day 28 assessment.

12 LABORATORY ANALYSIS

Laboratory methods for assessing vaccine (humoral) immunogenicity

Samples will first be screened by our in-house ELISA for the receptor binding domain (RBD) of the spike protein, with positive result subsequently confirmed by a surrogate virus neutralisation test (sVNT), and by a plaque reduction neutralisation test (PRNT).

Enzyme-linked immunosorbent assay (ELISA) for the SARS-CoV-2 spike RBD antibodies

We will use our in-house ELISA binding assay to screen sera at a 1:100 dilution for antibodies against the recombinant receptor-binding domain (RBD) of the SARS-CoV-2 spike protein and samples that reach an optical density cut off (0.5) will be considered positive by ELISA (36).

Protein expression and purification

The recombinant receptor-binding domain or RBD (residues 319–541) of the SARS-CoV-2 spike protein was cloned into a customised pFastBac vector (37), and RBD constructs were fused with an N-terminal gp67 signal peptide and a C-terminal His6 tag. Recombinant bacmid DNA was generated using the Bac-to-Bac system (Life Technologies, Thermo Fisher Scientific).

Baculovirus was generated by transfecting purified bacmid DNA into Sf9 cells using FuGENE HD (Promega, Madison, US), and subsequently used to infect suspension cultures of High Five cells (Life Technologies) at a multiplicity of infection (MOI) of 5 to 10. Infected High Five cells were incubated at 28 °C with shaking at 110 rpm for 72 h for protein expression. The supernatant was then concentrated using a Centramate cassette (10 kDa molecular weight cut-off for RBD, Pall Corporation, New York, USA). Spike RBD proteins were purified by Ni-NTA Superflow (Qiagen, Hilden, Germany), followed by size exclusion chromatography and buffer exchange to phosphate-buffered saline (PBS) (38).

ELISA binding assay
96-well ELISA plates (Nunc MaxiSorp, Thermo Fisher Scientific) are coated overnight with 100 ng per well of the purified recombinant RBD protein in PBS buffer. An additional plate is then coated overnight with PBS buffer only and used as control to subtract non-specific serum binding to the plate, i.e. serum-specific background noise (SSBN) normalisation approach (39). The plates coated with either purified recombinant protein or PBS were blocked with 100 μl of Chonblock blocking/sample dilution ELISA buffer (Chondrex Inc, Redmon, US) and incubated at room temperature for 2 h. Each serum or plasma sample was tested at a dilution of 1:100 in Chonblock blocking/sample dilution ELISA buffer and added to the ELISA wells of each plate for 2 h incubation at 37 °C. After extensive washing with PBS containing 0.1% Tween 20, horseradish peroxidase (HRP)-conjugated goat anti-human IgG (1:5,000, GE Healthcare) or HRP-conjugated goat anti-human IgM (1:5,000, GE Healthcare) are added for 1 h at 37 °C. The ELISA plates were then washed five times with PBS containing 0.1% Tween 20. Subsequently, 100 μL of HRP substrate (Ncm TMB One; New Cell and Molecular Biotech Co. Ltd, Suzhou, China) is added into each well. After 15 min incubation, the reaction is stopped by adding 50 μL of 2 M H_2SO_4 solution and analysed on a Sunrise (Tecan, Männedorf, Switzerland) absorbance microplate reader at 450 nm wavelength. Normalised results are obtained by calculating the difference between the OD of the purified recombinant protein-coated well and the PBS-coated well.

Surrogate virus neutralization test (sVNT)
SARS-CoV-2 surrogate virus neutralization test kits are obtained from GeneScript USA, Inc, New Jersey and manufacturer's instructions are followed to carry out the tests, as published previously (40). The test sera (10μL), positive and negative controls were diluted at 1:10 and mixed with an equal volume of horseradish peroxidase (HRP) conjugated to SARS-CoV-2 spike receptor binding domain (RBD) (6ng) and incubated for 30 minutes at 37°C. 100 μL of each mix is added to each well on the microtiter plate coated with ACE-2 receptor. The plate is sealed and incubated at room temperature for 15 minutes at 37°C. Plates are then washed with wash-solution, tapped dry, and 100 μL of 3,3',5,5'-Tetramethylbenzidine (TMB) solution is added to each well and incubated in the dark at room temperature for 15 minutes. Reaction is stopped by addition of 50μL of Stop Solution to each well and the absorbance read at 450 nm in an ELISA microplate reader. The assay validity was based off OD450 values for positive and negative falling in recommended values. Assuming the positive and negative controls gave the recommended OD450 values, the % inhibition of each serum is calculated as Inhibition (%) = (1 - Sample OD value/Negative Control OD value) 99 x100. Inhibition (%) of ≥20% is regarded as a positive result while inhibition of <20% is negative (41).

Plaque reduction neutralization test (PRNT)
Cell line
We maintain Vero E6 cells (ATCC CRL-1586) in Dulbecco's Modified Eagle Medium (DMEM) medium supplemented with 10% fetal bovine serum (FBS) and 100 U/mL of penicillin-

streptomycin. Sf9 cells (Spodoptera frugiperda ovarian cells, female, ATCC catalogue no. CRL-1711) and High Five cells (Trichoplusia ni ovarian cells, female; Thermo Fischer Scientific, Waltham, United States (US), catalogue number: B85502) were maintained in HyClone (GE Health Care, Chicago, US) insect cell culture medium.

Viruses and virus titration

SARS-CoV-2 virus has been isolated from a PCR confirmed COVID-19 patient from Hong Kong. The virus stock for SARS-CoV-2 is prepared in Vero E6 cell culture (ATCC CRL-1586) in minimal essential medium containing 2% fetal bovine serum, 100 units/mL penicillin and 100 μg/mL streptomycin. Virus aliquots are stored at -80 ˚C. Virus was titrated in serial half-log_{10} dilutions (from 0.5 log to 7 log) to obtain 50% tissue culture infectious dose ($TCID_{50}$) on 96-well tissue culture plates of Vero cells. The endpoint of viral dilution leading to CPE in 50% of inoculated wells was estimated by using the Reed Muench method and designated as one $TCID_{50}$.

Plaque reduction neutralisation tests (PRNT)

PRNT assays are performed in duplicate using 24-well tissue culture plates (TPP Techno Plastic Products AG, Trasadingen, Switzerland) in a biosafety level 3 facility. Serial two fold dilutions of serum samples re incubated with 30 plaque-forming units of virus for 1 h at 37 °C. The virus–serum mixtures are added onto Vero E6 cell monolayers and incubated 1 h at 37 °C in 5% CO_2 incubator. Then the plates are overlaid with 1% agarose in cell culture medium and incubated for 3 days when the plates are fixed and stained. Antibody titres are defined as the highest serum dilution that resulted in > 90% ($PRNT_{90}$) reduction in the number of plaques.

Laboratory methods for assessing cell-mediated immune responses

For assessing the cell-mediated immune responses to the vaccines, PBMCs collected from heparinized whole blood will be isolated by Ficoll-Paque density centrifugation. PBMCs will be tested by intracellular cytokine staining (ICS) to evaluate vaccine-specific $IFN\gamma^+$ $CD4^+$ and $IFN\gamma^+$ $CD8^+$ T cell responses as well as subjected to multi-parameter flow cytometry. Memory and antibody secreting B cells will be quantified using Nucleocapsid and Spike recombinant probes (42). Using overlapping structural peptide libraries for stimulation, activated and cytokine producing T cell responses, including $CD4^+$, Tfh, $CD8^+$ T cells, will be measured for Activation induced markers (AIM) and anti-viral Th1 and Th2 cytokine production by intracellular cytokine staining (ICS) (43).

Laboratory methods for assessing the effect of host genetics on vaccine-induced immune responses

If funding is available, we will also study how individuals with different host genetics backgrounds (e.g. antibody allotypes) induce different vaccine-specific antibody responses, the effect on duration of the immune responses, as well as the interaction with different COVID-19 vaccine boosters (44, 45). Human RNA will be isolated from PBMCs or other human cells and transcribed into cDNA. Subsequently, the template cDNA will be used for antibody allotyping.

Laboratory methods for systems serology

If funding is available, we will use an established multiplex bead assay (46) to perform systems serology on collected sera from a subset of participants, quantifying in parallel antibody specificity, isotype, IgG subclass and Fc binding profile, using recombinant Spike proteins that represent the ancestral Wuhan strain, VoC and relevant related viruses such as common cold viruses OC43, and SARS-CoV, and Nucleocapsid protein. The multiplex assay will be further adapted to quantify antibody mediated ACE2 inhibition for the sVNT (41, 47).

Additional laboratory testing of blood specimens

The remaining blood (whole blood, sera, plasma, or other human cells) will be de-identified, banked and stored for future testing. We plan to perform additional assays on blood specimens focused on biomarkers of immune protection and modulation of immune responses or disease severity.

13 ADVERSE EVENTS & ASSESSMENT OF SAFETY

Definitions of adverse events, adverse reaction and serious adverse reaction

Adverse event

An adverse event (AE) is any untoward medical occurrence in a clinical study subject to whom it has been administered a medicinal product, which does not necessarily have a causal relationship with the treatment.

Adverse reaction

An adverse reaction is an untoward and unintended response in a participant to an investigational medicinal product which is related to any dose administered to that participant.

All AEs must have their relationship to study intervention assessed by the investigator who examines and evaluates the participant based on temporal relationship and his/her clinical judgment. In a clinical trial, the study product must always be suspected. The degree of certainty about causality will be graded using the categories below:

Definitely Related – There is clear evidence to suggest a causal relationship, and other possible contributing factors can be ruled out. The clinical event, including an abnormal laboratory test result, occurs in a plausible time relationship to study intervention administration and cannot be explained by concurrent disease or other drugs or chemicals. The response to withdrawal of the study intervention (dechallenge) should be clinically plausible. The event must be pharmacologically or phenomenologically definitive, with use of a satisfactory rechallenge procedure if necessary.

Probably Related – There is evidence to suggest a causal relationship, and the influence of other factors is unlikely. The clinical event, including an abnormal laboratory test result, occurs within a reasonable time after administration of the study intervention, is unlikely to be attributed to concurrent disease or other drugs or chemicals, and follows a clinically reasonable response on withdrawal (dechallenge). Rechallenge information is not required to fulfill this definition.

Potentially Related – There is some evidence to suggest a causal relationship (e.g., the event occurred within a reasonable time after administration of the trial medication). However, other factors may have contributed to the event (e.g., the participant's clinical condition, other concomitant events). Although an AE may rate only as "possibly related" soon after discovery, it can be flagged as requiring more information and later be upgraded to "probably related" or "definitely related", as appropriate.

Not Related – A clinical event, including an abnormal laboratory test result, whose temporal relationship to study intervention administration makes a causal relationship improbable (e.g., the event did not occur within a reasonable time after administration of the study intervention) and in which other drugs or chemicals or underlying disease provides plausible

explanations (e.g., the participant's clinical condition, other concomitant treatments), OR the AE is completely independent of study intervention administration, and/or evidence exists that the event is definitely related to another aetiology. There must be an alternative, definitive aetiology documented by the clinician.

Solicited local adverse reactions
Severity definition of the overall impact of adverse events on daily activities are summarized in the below table:
Table 3. Severity definition of the overall impact of adverse events on daily activities.

Overall impact	Mild	Moderate	Severe
Any symptom	Symptoms were easily tolerated and did not interfere with any usual activities	Symptoms interfered with usual activities but did not prevent me from doing them	Symptoms were severe and prevented me from carrying out usual activities

Serious adverse event
An adverse event or suspected adverse reaction is considered "serious" if, in the view of either the investigator or sponsor, it results in any of the following outcomes:
- Death
- Life-threatening adverse event
- Inpatient hospitalization or prolongation of existing hospitalization
- Persistent or significant incapacity or substantial disruption of the ability to conduct normal life functions
- Congenital anomaly/birth defect.
- Important medical events that may not result in death, be life-threatening, or require hospitalization may be considered serious when, based upon appropriate medical judgment, they may jeopardize the patient or subject and may require medical or surgical intervention to prevent one of the outcomes listed above. (Examples: allergic bronchospasm requiring intensive treatment in an emergency room or at home, blood dyscrasias or convulsions that do not result in inpatient hospitalization, or the development of drug dependency or drug abuse.) [21 CFR 312.32(a)]

Assessment of adverse reaction and serious adverse events
The qualified investigators and medical nurses are responsible for assessing SAEs for causality and severity, and for final review and confirmation of accuracy of event information and assessments.

Time period and frequency for event assessment and follow-up
The occurrence of serious adverse event (SAE) may come to the attention of study personnel during study visits and interviews of a study participant presenting for medical care.

Investigators are not obligated to actively seek SAEs after study participation has concluded, however, if the investigator learns of any SAEs that did occur during participation in the study and is notified about the SAE after study participation has concluded, he/she should promptly document and report the event to DMID and Local Ethic.

All SAE will be captured on electronic database SAE tracking log. The reporting period is covered that occur after the subject first consents to participate in the study and throughout the duration of study, including post vaccination follow-up period. If the participant withdraws from the study, the last day of the reporting period would be the date of withdrawal. If participant withdraws (shortly) after the day of vaccination, participant will be followed up till end of adverse reaction monitoring, i.e. 7 days after the booster vaccination, or until the last symptom disappears, whichever the later.

After administration of vaccine, subjects will be observed for 15-30 minutes for any possible (immediate) adverse reactions due to vaccination. A consecutive 7 days follow-up period will be made refer to section 5.4 Adverse Reaction Monitoring. Apart from this, other general and emergency safety measures will also be conducted throughout the study period refer to section 5.4.

Reporting serious adverse event to local IRB
All parts of the research study are approved and/or given favourable opinion by Institutional Review Board of The University of Hong Kong / Hospital Authority Hong Kong West Cluster. It is an independent group of people to protect participants' safety, rights, wellbeing and dignity.

All SAEs are required to report local ethic accordance with local reporting timeline. Once the principal investigator (PI) or any delegated staff learns of any SAEs, they must complete SAE form and submit the report form to HKU/HA HKW IRB by internal post. Below contact ways are given:

The Secretary, HKU/HA HKW Institutional Review Board
Address: The Secretary, HKU/HA HKW Institutional Review Board Room 901, Administration Block, Queen Mary Hospital, 102, Pok Fu Lam Road,
Contact Number: 2255 3923
Enquiries E-mail: hkwirb@ha.org.hk

For fatal or life-threatening cases are required to report to local IRB with 7 calendar days from the first awareness of a SAE by the PI. Otherwise, other SAEs are to be reported to IRB within 15 calendar days from the first awareness by the PI.

Assessment of safety
Adverse reaction to vaccination
The safety profile of COVID-19 vaccines recognizes possible adverse reactions. The majority of people who receive COVID-19 vaccines, either mRNA or inactivated vaccines, do not generally experience severe reactions, and if minor problems occur, they usually subside within a few days. SAEs have been reported but are very rare. In the FDA safety assessment of BNT162b2 for emergency use authorization (EUA) based on a Phase 2/3 trial consisted of about 18800 vaccine and placebo recipients each, the proportion of participants who reported at least one serious adverse event were 0.4% in the vaccine group and 0.2% in the placebo group (48). FDA considered two events reported in the BNT162b2 group were possibly related to the vaccine, including shoulder injury possibly related to vaccine administration or to the vaccine itself, and lymphadenopathy involving axilla contralateral to the vaccine inject site. Therefore, the overall risk for participants who will receive vaccinations is low. Given the rarity of SAEs and the limited number of participants, we do not anticipate any occurrence of SAEs potentially related to vaccination within our study. For adverse reaction monitoring, refer to section 5.4 for details. For stopping rule, refer to section 7.7 for details.

Volume of blood taking

At each blood drawing session up to 40 ml of blood will be collected. This volume of blood is well within the recommended volume of blood collection, i.e. the volume of blood drawn should not exceed 1% of total blood volume at any single blood drawing session and should not exceed 3% of the total blood volume over a period 4 weeks, of which the total blood volume is determined based on the body weight of the participant assuming 80 mL blood/kg body weight (49, 50).

Discomfort from collection of blood & administration of vaccinations

As for blood sample collection, subjects may experience slight pain or minor bruising. The risks associated with blood-taking are low. Only registered phlebotomists or trained nurses will collect blood samples. Only trained nurses will administer injections, and the nurses will be trained according to the vaccination administration, handling, and storage guidelines listed in the 13th Edition Epidemiology and Prevention of Vaccine-Preventable Diseases ("The Pink Book") published by the Centers for Disease Control and Prevention (US CDC) in 2015 (51).

Protection of participants from respiratory infections

All research personnel who will be involved in study activities that require face-to-face interaction with participants will be offered influenza vaccination for the prevention of influenza virus infection and to participate in the COVID-19 vaccination programme. While on-duty during face-to-face interaction with participants, research personnel will be required to wear a face mask and advised to perform hand hygiene regularly. Research personnel will also be advised to monitor their own health and temperature on a daily basis and report to the project manager immediately if any symptoms including fever appear for follow-up actions.

Protection of research personnel from respiratory infections

All research personnel who will be involved in recruiting and contacting participants will be offered influenza vaccination for the prevention of influenza virus infection and to participate in the COVID-19 vaccination programme. All research personnel involved in blood specimen collection will be provided with suitable equipment and proper training to ensure safety and adequate infection prevention and control according to the WHO Guidelines on Drawing Blood: Best Practices in Phlebotomy (51).

During the arrangement of clinic visit appointment for blood collection or vaccination about a week prior to receipt of the booster dose, participants will be screened for travel history and other risk factors of exposure to COVID-19 in the past 14 days, and only those who deemed having a low risk of COVID-19 will be enrolled and randomized. On the day of each study visit, temperature and symptom checks will be used as the first part of the screening process before entering the premises, and participant who have a temperature above 37.5°C and/or have respiratory symptoms will be advised to return home.

To minimize the contact time between participants and research personnel in light of the COVID-19 pandemic and the risk of SARS-CoV-2 transmission, all questionnaires may be administered via telephone call or face-to-face interview, or in the form of an online survey, unless otherwise specified in the study protocol or appendices.

Other general and emergency safety measures

All trial-related activities, including enrolment and follow-up, will be conducted by our delegated research team members from the School of Public Health, the University of Hong

Kong, stationed at the HKUSPH research sites, designated ECC, EDC and designated rooms rented from private medical centres. Research staff designated for each centre will consist of at least one registered/enrolled nurse or trained phlebotomist and one research assistant for trial-related activities such as recruitment, handling and dispensing trial vaccine, collecting biological specimens from participants and follow-up. Each research team stationed there will be equipped with emergency kit with Ambu bag, adrenaline injection or EpiPen, and BP monitor in case of any acute reaction to vaccine administration. Medical doctor for the study will station at the designated centre when necessary.

14 STATISTICAL ANALYSIS

Statistical methods to be employed

For the serological immunogenicity assessment, antibody titres will be log-transformed before all statistical analysis and will be presented as geometric mean titres. The GMT of SARS-CoV-2 neutralizing antibodies and the geometric mean concentrations (GMC) of SARS-CoV-2 S1 binding IgG at day 28 will be compared between study arms using Mann-Whitney U tests. For each study arm we will compare compare ratios of baseline to different time points after boost vaccination using Wilcoxon signed rank tests. Additional secondary analyses will involve parametric and non-parametric comparisons of antibody titers at various time points.

For the cellular immunogenicity assessment, differences in cell population frequencies in the same individual will be assessed using the Wilcoxon signed rank test, correcting for false discovery rates. Mann–Whitney U-test will be used for comparisons of T-cell responses between study arms. Spearman correlation will be used for correlating T-cell responses with serological immunogenicity assessment.

Reactogenicity endpoints will be described as frequency (%) with the 95% CI of the adverse reactions or events at different time points. We will compare the proportions of the participants with adverse reactions or events between groups in the same or different time points using the chi-squared test or Fisher’s exact test.

Procedure for accounting for any missing data

If missing data are present in the online data collection tool REDCap, we will first check with the participants directly to attempt to complete those variables. In the analyses, we will use multiple imputation (52) with 10 imputed datasets to replace missing values on outcome and predictor variables. If 10 imputed datasets are not sufficient to ensure stability of estimates we will use 20 imputed datasets. Multiple imputation makes maximum use of available data and maximizes statistical power while requiring less strict theoretical assumptions than to a complete case analysis, or single imputation of mean values. We note that this is now one of the preferred (and standard) methods for analyzing clinical trials data (53).

Procedure for reporting any deviations from the original statistical plan

Any deviations from the original statistical plan will be described and justified in the final report.

Selection of participants to be included in the analyses

We will include all available data on participants who have received the booster dose. Data from individuals who consent to participate but do not receive the booster dose will not be included in analyses.

15 QUALITY CONTROL AND QUALITY ASSURANCE

Our research personnel will follow Good Clinical Practice (GCP) and our standard operating procedures to ensure proper conduct throughout the study. The trial will be conducted in strict compliance with the approved study protocol. Only registered and enrolled nurses will be allowed to administer vaccinations, and only trained phlebotomists or registered/enrolled nurses will collect blood samples. Other research personnel will also be trained and briefed to collect data, recruit participants, and answer questions from the participants or their family members.

RELEVANCE, SIGNIFICANCE AND VALUE

Our study will provide pioneer evidence into the effects of using a different vaccine platform as a booster dose to a complete homologous 2-dose regime and comparative data between homologous and heterologous booster. Moreover, it will provide insights on the potential need for vaccination boosters and the best booster vaccination regime. This information together with data collected on reactogenicity and safety could inform vaccination policy locally and internationally.

16 ETHICS

The study will be conducted in compliance with this protocol, the Declaration of Helsinki (54), Good Clinical Practice, and the applicable regulatory requirements. Written informed consent will be obtained by our research staff directly from eligible participants after thoroughly explaining the study design, purpose and necessity of randomization to each potential participant. Rights and well-being of participants will be well protected during the whole study period, participation in the study is on a purely voluntary basis and participants are free to withdraw their consent and participation at any stage of the study with no explanation and without any prejudice.

There are no major risks associated with receipt of COVID-19 vaccination, and we will monitor the expected mild transient reactions. The process of collecting blood is associated with minimal risk, a small number of participants may have local reactions with the symptom of mild transient pain and bruising, but this will not influence their health. The process of self-collection of respiratory swabs for rapid antigen tests is associated with minimal risk, a small number of participants may feel minor discomfort, but this will not influence their health. Another potential risk is loss of confidentiality due to data breach, but as vaccination status, and immune and other general physiological metrics do not carry large social stigma, these costs would be limited, and we will make every effort to control the risk. Participants in the study, as members of the whole society, will also benefit indirectly from this trial as the findings will make important contributions to our knowledge regarding the use of COVID-19 vaccines to boost the immune response in fully-vaccinated individuals that will inform evidence-based decisions about COVID-19 vaccination programs in Hong Kong, and elsewhere.

17 BIOLOGICAL SAMPLE HANDLING AND KEEPING

All biological samples including blood, sera and human cells such as PBMCs will be stored in the School of Public Health, the University of Hong Kong, and tested by the research team at the University of Hong Kong or their collaborators at other local and/or overseas academic

institutions for testing of immunity against specific respiratory viruses including genetic component of human immunity, or future research studies about other health problems. Biological samples will be stored for up to 10 years after the conclusion of the study to allow sufficient time to complete these tests.

18 DATA HANDLING AND RECORD KEEPING

REDCap (Research Electronic Data Capture) is a software application and workflow methodology designed to collect and manage data for research studies (55). REDCap is a secure, web-based application designed to support data capture for research studies, providing: 1) an intuitive interface for validated data entry; 2) audit trails for tracking data manipulation and user activity; 3) automated export procedures for data downloads to Excel, PDF, and common statistical packages (SPSS, SAS, Stata, R); and 4) procedures for importing data from external sources.

Data for the questionnaire will be entered by the research staff directly to the REDCap electronic data capture system at the time of contact with the participants, or entered by the participants directly when the questionnaires are being administered as an online survey. All other data obtained from the participants will be extracted into the secure REDCap database. This extraction will be facilitated by authorized researchers. The servers supporting the project database are secured by the University of Hong Kong in password-protected computers. All records will be entered using a personal identification code, making the records unidentifiable in REDCap. The link between the protected health information and personal identification code will be stored separately. All identifying data is anticipated to remain on the University of Hong Kong REDCap server in a locked cabinet in a locked room. Entry of personal data to the REDCap will be minimized as far as possible, and data that may be considered as identifiable will be protected in the database such that it cannot be downloaded when the other anonymized study data are downloaded for analysis. The University of Hong Kong will set up the REDCap for real-time quality control check, tracking and preliminary data cleaning. All data will be stored in the School of Public Health, The University of Hong Kong. All personal data will be kept confidential. Anonymized data containing visit and test dates will be shared, via REDCap, with collaborators at the University of Chicago and U.S. National Institutes of Health. Anonymized data in spreadsheets may also be shared with collaborators at local and international universities or research institutes. Only fully deidentified data will be shared with researchers outside the study. Data from this study may be used in presentations and papers. Any potentially identifying information such as visit dates will not be shown.

19 FINANCING AND INSURANCE

This study is financially supported by the Hong Kong Food and Health Bureau and Health and Medical Research Fund (Ref. No. COVID19F09).

Participants of this study will be covered by the Indemnity Fund for Adverse Events Following Immunization with Coronavirus Disease-2019 Vaccines (AEFI Fund) set up by the Hong Kong government. The Fund is to provide financial support to eligible individuals who have proof of suffering unexpected serious adverse events (SAEs) (including death and serious injury) associated with COVID-19 vaccine. In addition, the School of Public Health, the University of Hong Kong has also purchased clinical trials insurance that will cover treatment for any illness or injury caused by the study drug or any study procedures that the participant would not have

suffered if he/she were not participating in the study to the extent that these costs are not paid for by the participant's health insurance fund or third party.

All forms of medical diagnosis and treatment – whether routine or experimental – involve some risk of injury. In spite of all precautions, a rare number of participants might develop medical complications from participating in this study. If such complications arise, the study staff will assist the participant in obtaining appropriate medical treatment. Participants will not be charged for this treatment. If the participant suffer from a loss resulting from such an injury, he/she will be compensated. However, this will not apply if the injury results from the participant's own fault or intention.

20 PUBLICATION POLICY

The results will be published in international peer-reviewed journals.

21 SUPPLEMENTS

Appendix 1: Recruitment Screening Questionnaire
Appendix 2: Participant Information Sheet
Appendix 3: Participant Consent Form
Appendix 4: Poster
Appendix 5: Enrolment Questionnaire
Appendix 6: Post-vaccination Reaction Questionnaire
Appendix 7: Mid-year Assessment Questionnaire
Appendix 8: End-of-study Assessment Questionnaire
Appendix 9: Hospitalization Questionnaire
Appendix 10: Death Questionnaire
Appendix 11: Participant Withdrawal Form
Appendix 12: Vaccination Card
Appendix 13: Additional Informed Consent – Durability of immunity
Appendix 14: Additional Informed Consent – Active surveillance for illness
Appendix 15: Active surveillance Initiation
Appendix 16: Active surveillance Form
Appendix 17: Illness Symptom Diary
Appendix 18: Illness Recovery Questionnaire

22 REFERENCES

1. Krammer F. SARS-CoV-2 vaccines in development. Nature. 2020;586(7830):516-27.
2. Polack FP, Thomas SJ, Kitchin N, Absalon J, Gurtman A, Lockhart S, et al. Safety and Efficacy of the BNT162b2 mRNA Covid-19 Vaccine. N Engl J Med. 2020;383(27):2603-15.
3. Voysey M, Clemens SAC, Madhi SA, Weckx LY, Folegatti PM, Aley PK, et al. Safety and efficacy of the ChAdOx1 nCoV-19 vaccine (AZD1222) against SARS-CoV-2: an interim analysis of four randomised controlled trials in Brazil, South Africa, and the UK. Lancet. 2021;397(10269):99-111.
4. Zhang Y, Zeng G, Pan H, Li C, Hu Y, Chu K, et al. Safety, tolerability, and immunogenicity of an inactivated SARS-CoV-2 vaccine in healthy adults aged 18-59 years: a randomised, double-blind, placebo-controlled, phase 1/2 clinical trial. Lancet Infect Dis. 2021;21(2):181-92.

5. Tanriover MD, Doganay HL, Akova M, Guner HR, Azap A, Akhan S, et al. Efficacy and safety of an inactivated whole-virion SARS-CoV-2 vaccine (CoronaVac): interim results of a double-blind, randomised, placebo-controlled, phase 3 trial in Turkey. Lancet. 2021;398(10296):213-22.
6. Wu Z, Hu Y, Xu M, Chen Z, Yang W, Jiang Z, et al. Safety, tolerability, and immunogenicity of an inactivated SARS-CoV-2 vaccine (CoronaVac) in healthy adults aged 60 years and older: a randomised, double-blind, placebo-controlled, phase 1/2 clinical trial. Lancet Infect Dis. 2021;21(6):803-12.
7. Wan EYF, Chui CSL, Lai FTT, Chan EWY, Li X, Yan VKC, et al. Bell's palsy following vaccination with mRNA (BNT162b2) and inactivated (CoronaVac) SARS-CoV-2 vaccines: a case series and nested case-control study. Lancet Infect Dis. 2021.
8. Pottegard A, Lund LC, Karlstad O, Dahl J, Andersen M, Hallas J, et al. Arterial events, venous thromboembolism, thrombocytopenia, and bleeding after vaccination with Oxford-AstraZeneca ChAdOx1-S in Denmark and Norway: population based cohort study. BMJ. 2021;373:n1114.
9. Dodd RH, Pickles K, Nickel B, Cvejic E, Ayre J, Batcup C, et al. Concerns and motivations about COVID-19 vaccination. Lancet Infect Dis. 2021;21(2):161-3.
10. Saiag E, Goldshmidt H, Sprecher E, Ben-Ami R, Bomze D. Immunogenicity of a BNT162b2 vaccine booster in health-care workers. Lancet Microbe. 2021.
11. Choi A, Koch M, Wu K, Chu L, Ma L, Hill A, et al. Safety and immunogenicity of SARS-CoV-2 variant mRNA vaccine boosters in healthy adults: an interim analysis. Nat Med. 2021;27(11):2025-31.
12. Flaxman A, Marchevsky NG, Jenkin D, Aboagye J, Aley PK, Angus B, et al. Reactogenicity and immunogenicity after a late second dose or a third dose of ChAdOx1 nCoV-19 in the UK: a substudy of two randomised controlled trials (COV001 and COV002). Lancet. 2021.
13. Pan H, Wu Q, Zeng G, Yang J, Jiang D, Deng X, et al. Immunogenicity and safety of a third dose, and immune persistence of CoronaVac vaccine in healthy adults aged 18-59 years: interim results from a double-blind, randomized, placebo-controlled phase 2 clinical trial. medRxiv2021.
14. Werbel WA, Boyarsky BJ, Ou MT, Massie AB, Tobian AAR, Garonzik-Wang JM, et al. Safety and Immunogenicity of a Third Dose of SARS-CoV-2 Vaccine in Solid Organ Transplant Recipients: A Case Series. Ann Intern Med. 2021.
15. Benotmane I, Gautier G, Perrin P, Olagne J, Cognard N, Fafi-Kremer S, et al. Antibody Response After a Third Dose of the mRNA-1273 SARS-CoV-2 Vaccine in Kidney Transplant Recipients With Minimal Serologic Response to 2 Doses. JAMA. 2021.
16. Bar-On YM, Goldberg Y, Mandel M, Bodenheimer O, Freedman L, Kalkstein N, et al. Protection of BNT162b2 Vaccine Booster against Covid-19 in Israel. N Engl J Med. 2021;385(15):1393-400.
17. Shaw RH, Stuart A, Greenland M, Liu X, Van-Tam JSN, Snape MD, et al. Heterologous prime-boost COVID-19 vaccination: initial reactogenicity data. Lancet. 2021;397(10289):2043-6.
18. Liu X, Shaw RH, Stuart ASV, Greenland M, Dinesh T, Provstgaard-Morys S, et al. Safety and Immunogenicity Report from the Com-COV Study – a Single-Blind Randomised Non-Inferiority Trial Comparing Heterologous And Homologous Prime-Boost Schedules with An Adenoviral Vectored and mRNA COVID-19 Vaccine. SSRN Electronic Journal2021.
19. Borobia AM, Carcas AJ, Pérez Olmeda MT, Castaño L, Jesús Bertrán M, García-Pérez J, et al. Reactogenicity and Immunogenicity of BNT162b2 in Subjects Having Received a First Dose of ChAdOx1s: Initial Results of a Randomised, Adaptive, Phase 2 Trial (CombiVacS). SSRN Electronic Journal2021.

20. Hillus D, Schwarz T, Tober-Lau P, Hastor H, Thibeault C, Kasper S, et al. Safety, reactogenicity, and immunogenicity of homologous and heterologous prime-boost immunisation with ChAdOx1-nCoV19 and BNT162b2: a prospective cohort study. medRxiv2021.
21. Schmidt T, Klemis V, Schub D, Mihm J, Hielscher F, Marx S, et al. Immunogenicity and reactogenicity of a heterologous COVID-19 prime-boost vaccination compared with homologous vaccine regimens. medRxiv2021.
22. He Q, Mao Q, An C, Zhang J, Gao F, Bian L, et al. Heterologous prime-boost: breaking the protective immune response bottleneck of COVID-19 vaccine candidates. Emerg Microbes Infect. 2021;10(1):629-37.
23. Xia S, Zhang Y, Wang Y, Wang H, Yang Y, Gao GF, et al. Safety and immunogenicity of an inactivated SARS-CoV-2 vaccine, BBIBP-CorV: a randomised, double-blind, placebo-controlled, phase 1/2 trial. Lancet Infect Dis. 2021;21(1):39-51.
24. Cowling BJ, Wong IOL, Shiu EYC, Lai AYT, Cheng SMS, Chaothai S, et al. Strength and durability of antibody responses to BNT162b2 and CoronaVac. medRxiv2022.
25. Chia WN, Zhu F, Ong SWX, Young BE, Fong S-W, Le Bert N, et al. Dynamics of SARS-CoV-2 neutralising antibody responses and duration of immunity: a longitudinal study. Lancet Microbe. 2021;2(6):e240-e9.
26. Yao L, Wang GL, Shen Y, Wang ZY, Zhan BD, Duan LJ, et al. Persistence of Antibody and Cellular Immune Responses in Coronavirus Disease 2019 Patients Over Nine Months After Infection. J Infect Dis. 2021;224(4):586-94.
27. Ogbe A, Pace M, Bittaye M, Tipoe T, Adele S, Alagaratnam J, et al. Durability of ChAdOx1 nCov-19 vaccination in people living with HIV. JCI Insight. 2022.
28. Mateus J, Dan JM, Zhang Z, Rydyznski Moderbacher C, Lammers M, Goodwin B, et al. Low-dose mRNA-1273 COVID-19 vaccine generates durable memory enhanced by cross-reactive T cells. Science. 2021;374(6566).
29. Levin EG, Lustig Y, Cohen C, Fluss R, Indenbaum V, Amit S, et al. Waning Immune Humoral Response to BNT162b2 Covid-19 Vaccine over 6 Months. N Engl J Med. 2021.
30. McMenamin ME, Nealon J, Lin Y, Wong JY, Cheung JK, Lau EHY, et al. Vaccine effectiveness of two and three doses of BNT162b2 and CoronaVac against COVID-19 in Hong Kong. 2022.
31. Cowling BJ, Perera RA, Fang VJ, Chan KH, Wai W, So HC, et al. Incidence of influenza virus infections in children in Hong Kong in a 3-year randomized placebo-controlled vaccine study, 2009-2012. Clin Infect Dis. 2014;59(4):517-24.
32. Cowling BJ, Ng S, Ma ESK, Cheng CKY, Wai W, Fang VJ, et al. Protective Efficacy of Seasonal Influenza Vaccination against Seasonal and Pandemic Influenza Virus Infection during 2009 in Hong Kong. Clin Infect Dis. 2010;51(12):1370-9.
33. Cowling BJ, Ng S, Ma ES, Fang VJ, So HC, Wai W, et al. Protective efficacy against pandemic influenza of seasonal influenza vaccination in children in Hong Kong: a randomized controlled trial. Clin Infect Dis. 2012;55(5):695-702.
34. Klick B, Durrani S, Chan KH, Ip DK, Chou ES, Kwok HK, et al. Live attenuated seasonal and pandemic influenza vaccine in school-age children: a randomized controlled trial. Vaccine. 2013;31(15):1937-43.
35. Advisory Panel on COVID-19 Vaccines, The Government of the Hong Kong Special Administrative Region. Report on Evaluation of Safety, Efficacy and Quality of Comirnaty COVID-19 mRNA Vaccine (BNT162b2) Concentrate for Dispersion for Injection. 2021 January 22, 2021.
36. Perera RA, Mok CK, Tsang OT, Lv H, Ko RL, Wu NC, et al. Serological assays for severe acute respiratory syndrome coronavirus 2 (SARS-CoV-2), March 2020. Euro Surveill. 2020;25(16).

37. Ekiert DC, Friesen RH, Bhabha G, Kwaks T, Jongeneelen M, Yu W, et al. A highly conserved neutralizing epitope on group 2 influenza A viruses. Science. 2011;333(6044):843-50.
38. Yuan M, Wu NC, Zhu X, Lee CD, So RTY, Lv H, et al. A highly conserved cryptic epitope in the receptor-binding domains of SARS-CoV-2 and SARS-CoV. Science. 2020.
39. Moritz CP, Tholance Y, Lassabliere F, Camdessanche JP, Antoine JC. Reducing the risk of misdiagnosis of indirect ELISA by normalizing serum-specific background noise: The example of detecting anti-FGFR3 autoantibodies. J Immunol Methods. 2019;466:52-6.
40. Perera R, Ko R, Tsang OTY, Hui DSC, Kwan MYM, Brackman CJ, et al. Evaluation of a SARS-CoV-2 Surrogate Virus Neutralization Test for Detection of Antibody in Human, Canine, Cat, and Hamster Sera. J Clin Microbiol. 2021;59(2).
41. Tan CW, Chia WN, Qin X, Liu P, Chen MI, Tiu C, et al. A SARS-CoV-2 surrogate virus neutralization test based on antibody-mediated blockage of ACE2-spike protein-protein interaction. Nat Biotechnol. 2020;38(9):1073-8.
42. Juno JA, Tan H-X, Lee WS, Reynaldi A, Kelly HG, Wragg K, et al. Humoral and circulating follicular helper T cell responses in recovered patients with COVID-19. Nat Med. 2020;26(9):1428-34.
43. Cohen CA, Li APY, Hachim A, Hui DSC, Kwan MYW, Tsang OTY, et al. SARS-CoV-2 specific T cell responses are lower in children and increase with age and time after infection. Nat Commun. 2021;12(1).
44. Kratochvil S, McKay PF, Chung AW, Kent SJ, Gilmour J, Shattock RJ. Immunoglobulin G1 Allotype Influences Antibody Subclass Distribution in Response to HIV gp140 Vaccination. Front Immunol. 2017;8.
45. Lemke MM, McLean MR, Lee CY, Lopez E, Bozich ER, Rerks-Ngarm S, et al. A systems approach to elucidate personalized mechanistic complexities of antibody-Fc receptor activation post-vaccination. Cell Reports Medicine. 2021;2(9):100386.
46. McLean MR, Madhavi V, Wines BD, Hogarth PM, Chung AW, Kent SJ. Dimeric Fcγ Receptor Enzyme-Linked Immunosorbent Assay To Study HIV-Specific Antibodies: A New Look into Breadth of Fcγ Receptor Antibodies Induced by the RV144 Vaccine Trial. The Journal of Immunology. 2017;199(2):816-26.
47. Tan CW, Chia WN, Young BE, Zhu F, Lim BL, Sia WR, et al. Pan-Sarbecovirus Neutralizing Antibodies in BNT162b2-Immunized SARS-CoV-1 Survivors. N Engl J Med. 2021;385(15):1401-6.
48. U.S. Food and Drug Administration. EUA for an Unapproved Product – Review Memorandum for the Pfizer-BioNTech COVID-19 vaccine. 2020.
49. Howie SR. Blood sample volumes in child health research: review of safe limits. Bull World Health Organ. 2011;89(1):46-53.
50. European Commission. Ethical considerations for clinical trials on medicinal products conducted with minors. 2017 September 18, 2017.
51. Centers for Disease Control and Prevention. Epidemiology and Prevention of Vaccine-Preventable Diseases. Washington D.C. : Public Health Foundation; 2015.
52. Schafer JL. Multiple imputation: a primer. Stat Methods Med Res. 1999;8(1):3-15.
53. Molenberghs G, Kenward MG. Missing data in clinical studies. Hoboken: John Wiley & Sons Inc.; 2007.
54. World Health Organization. Practical Guidelines for Infection Control in Health Care Facilities 2004 [Available from: http://www.wpro.who.int/publications/docs/practical_guidelines_infection_control.pdf.
55. Harris PA. Research Electronic Data Capture (REDCap) - planning, collecting and managing data for clinical and translational research. BMC Bioinformatics. 2012;13(12):A15.
